# Therapeutic Equivalence of Biosimilar and Reference Biologic Drugs in Rheumatoid Arthritis: A Systematic Review and Meta-analysis

**DOI:** 10.1001/jamanetworkopen.2023.15872

**Published:** 2023-05-26

**Authors:** Bruna de Oliveira Ascef, Matheus Oliveira Almeida, Ana Cristina de Medeiros-Ribeiro, Danieli Castro Oliveira de Andrade, Haliton Alves de Oliveira Junior, Patrícia Coelho de Soárez

**Affiliations:** 1 Departamento de Medicina Preventiva, Faculdade de Medicina, Universidade de Sao Paulo, Sao Paulo, Brazil; 2 Programa de Pos-Graduaçao em Fisioterapia, Universidade Ibirapuera, Sao Paulo, Brazil; 3 Disciplina de Reumatologia do Hospital das Clinicas da Faculdade de Medicina da Universidade de Sao Paulo, Sao Paulo, Brazil; 4 Disciplina de Reumatologia do Hospital das Clinicas da Faculdade de Medicina da Universidade de Sao Paulo, Sao Paulo, Brazil; 5 Health Technology Assessment Unit, Hospital Alemao Oswaldo Cruz, Sao Paulo, Brazil; 6 Departamento de Medicina Preventiva, Faculdade de Medicina, Universidade de Sao Paulo, Sao Paulo, Brazil

## Abstract

**Importance:**

Biosimilar drugs are potentially lower-cost versions of biologics that may improve access to therapy. However, there is a lack of adequate systematic reviews demonstrating equivalence between these drugs for the treatment of rheumatoid arthritis (RA).

**Objectives:**

To assess the efficacy, safety, and immunogenicity associated with biosimilars of adalimumab, etanercept, and infliximab compared with their reference biologics in patients with RA.

**Data Sources:**

MEDLINE via PubMed, Embase, Cochrane Central Register of Controlled Trials, and LILACS databases were searched from inception to September 2021.

**Study Selection:**

Head-to-head randomized clinical trials (RCTs) of biosimilars of adalimumab, etanercept, and infliximab and their biologic reference drugs for RA were assessed.

**Data Extraction and Synthesis:**

Two authors independently abstracted all data. Meta-analysis was conducted with bayesian random effects using relative risks (RRs) for binary outcomes and standardized mean differences (SMDs) for continuous outcomes, with 95% credible intervals (CrIs) and trial sequential analysis. Specific domains were assessed for the risk of bias in equivalence and noninferiority trials. This study was conducted according to the Preferred Reporting Items for Systematic Reviews and Meta-analyses (PRISMA) guideline.

**Main Outcomes and Measures:**

Equivalence was tested using prespecified margins for the American College of Rheumatology criteria, with at least 20% improvement in the core set measures (ACR20) (ie, RR, 0.94 to 1.06), and for the Health Assessment Questionnaire–Disability Index (HAQ-DI) (ie, SMD, −0.22 to 0.22). Secondary outcomes included 14 items measuring safety and immunogenicity.

**Results:**

A total of 25 head-to-head trials provided data on 10 642 randomized patients with moderate to severe RA. Biosimilars met equivalence with reference biologics in terms of ACR20 response (24 RCTs with 10 259 patients; RR, 1.01; 95% CrI, 0.98 to 1.04; τ^2^ = 0.000) and change of HAQ-DI scores (14 RCTs with 5579 patients; SMD, −0.04; 95% CrI, −0.11 to 0.02; τ^2^ = 0.002) considering prespecified margins of equivalence. Trial sequential analysis found evidence for equivalence for ACR20 since 2017 and HAQ-DI since 2016. Overall, biosimilars were associated with similar safety and immunogenicity profiles compared with reference biologics.

**Conclusion and Relevance:**

In this systematic review and meta-analysis, biosimilars of adalimumab, infliximab, and etanercept were associated with clinically equivalent treatment effects compared with their reference biologics for the treatment of RA.

## Key Points

**Question:**

Are biosimilars of adalimumab, etanercept, and infliximab associated with equivalent treatment compared with their reference biologic drugs for the management of rheumatoid arthritis?

**Findings:**

In this systematic review and meta-analysis of 25 randomized clinical trials that included data on 10 642 patients with rheumatoid arthritis, patients using biosimilars had equivalent clinical responses and functional capacity compared with patients using reference biologic drugs.

**Meaning:**

These findings suggest that biosimilars may yield therapeutically equivalent outcomes compared with reference biologic drugs for the management of rheumatoid arthritis.

## Teaser

This systematic review and meta-analysis assesses the efficacy, safety, and immunogenicity associated with biosimilars of adalimumab, etanercept, and infliximab compared with their reference biologics for patients with rheumatoid arthritis.

## Introduction

Rheumatoid arthritis (RA) is a debilitating inflammatory condition that primarily affects the joints, reducing physical function and quality of life.^1^ Globally, approximately 20 million people have RA.^2^ Tumor necrosis factor-α inhibitors (TNFIs), such as infliximab, adalimumab, and etanercept, are biologic disease-modifying antirheumatic drugs used worldwide to treat RA.^3,4,5,6^ While robust evidence has demonstrated the efficacy and safety of TNFIs for managing RA, the high costs of TNFIs can limit treatment access to these drugs worldwide.^6,7^

Biosimilar drugs are potentially lower-cost versions of TNFIs that have been used in RA treatment and have the potential to improve access to therapy.^6,8,9,10,11,12^ International guidelines and consensus recommend biosimilars for RA management.^5,6,8,13,14,15,16,17,18^ Nonetheless, these recommendations have relied mainly on single trials or expert consensus, with only 2 guidelines using meta-analysis.^17,18^

Previous systematic reviews^18,19,20,21,22,23,24,25,26,27,28,29^ attempted to compile evidence on similar efficacy and safety between biosimilars and reference products for RA. Nonetheless, most of these reviews^18,19,21,23,24,27^ provided only qualitative summaries, which are insufficient for decision-making. The few reviews^20,22,25,26,28,29^ that attempted quantitative analyses did not use appropriate equivalence testing methods, resulting in imprecise conclusions.^30^

In this study, we conducted a systematic review and meta-analysis of randomized clinical trials (RCTs) comparing biosimilar drugs and reference products of the most prescribed TNFIs (infliximab, adalimumab, and etanercept) for RA.^31,32,33,34,35,36^ We addressed previous reviews’ limitations by using equivalence testing via prespecified margins and bayesian meta-analyses.

## Methods

For this systematic review and meta-analysis, a multidisciplinary panel developed the protocol (PROSPERO identification: CRD42019137152) and published it elsewhere.^37^ Changes to the protocol are presented in eAppendix 1 in Supplement 1. We followed the Preferred Reporting Items for Systematic Reviews and Meta-analyses (PRISMA) reporting guideline^38^ and a specific guidance for equivalence and noninferiority systematic reviews.^39^

### Eligibility Criteria

We included RCTs investigating patients of both sexes with RA. No limitations regarding sociodemographic characteristics or disease severity or duration were imposed. Interventions of interest were biosimilars of adalimumab, etanercept, and infliximab. Comparators of interest were the reference biologic drugs (ie, adalimumab, etanercept, and infliximab originals). No restrictions were imposed on trial design, dosages, treatment schedules, or cotreatments. The full eligibility criteria are given in eAppendix 2 in Supplement 1.

### Evidence Sources, Search Strategy, and Selection Process

A detailed search strategy description is available in our published protocol^37^ and reproduced in eAppendix 3 in Supplement 1. We conducted a comprehensive literature search in MEDLINE (via PubMed), Embase, Cochrane Central Register of Controlled Trials, and LILACS from database inception to September 7, 2021. We also searched for unpublished or ongoing trials in 4 trial registry databases and performed citation searches of all included studies. No language limitation was imposed. Two investigators (B.O.A. and M.O.A.) independently assessed titles, abstracts, and full-length articles against the eligibility criteria (eAppendix 4 in Supplement 1).

### Data Extraction

Whenever available, we collected the population per-protocol (PP) data (eAppendix 5 in Supplement 1). Two investigators (B.O.A. and M.O.A.) extracted all data independently, and discrepancies were solved via a consensus or consultation with a third reviewer (P.C.S.). The complete list of variables extracted for each trial is shown in eAppendix 5 in Supplement 1.

### Outcomes

#### Primary Outcomes: Efficacy

The prespecified primary efficacy end point was the treatment success at 6 months, according to the American College of Rheumatology 20% response criteria (ACR20), which requires at least a 20% improvement in the core set measures for a patient to reach improvement. ACR20 was summarized as relative risk (RR), with an RR greater 1.0, indicating a higher response probability with biosimilar drugs compared with reference biologics (eAppendix 6 in Supplement 1).

We also prespecified the Health Assessment Questionnaire–Disability Index (HAQ-DI) at 6 months of follow-up as a primary outcome, which measures disability and is patient-reported. HAD-DI was presented as a standardized mean difference (SMD), Cohen effect size and an SMD less than 0 indicate a better outcome for biosimilar drugs than in reference biologics (eAppendix 6 in Supplement 1).

#### Secondary Outcomes

##### Efficacy

We included the ACR 50% response criteria (ACR50) and ACR 70% response criteria (ACR70) as secondary efficacy outcomes. Due to the volume of data, additional secondary outcomes of efficacy prespecified in the protocol will be shared in future publications (eAppendix 7 in Supplement 1).

##### Safety and Immunogenicity

The prespecified safety and immunogenicity outcomes included treatment-emergent adverse events (TEAEs), serious adverse events, special adverse events, mortality, discontinuation rates, positive antidrug antibodies (ADAs) formation, and positive neutralizing antibodies (NAbs) (eAppendix 8 in Supplement 1).

### Assessment of Risk of Bias

We used the Cochrane Risk of Bias tool (version 1.0)^40^ to assess the risk of bias in each trial, using the ratings of 2 independent reviewers (B.O.A. and M.O.A.). Additionally, we addressed specific domains of equivalence or noninferiority trials^39^ (eAppendix 9 in Supplement 1).

### Statistical Analysis

#### Data Synthesis

The approaches to approximate means and SDs from the reported statistics are shown in eAppendix 10 in Supplement 1. Binary outcomes were summarized using the RR as a metric, whereas continuous outcomes were summarized as SMDs. We combined results across trials using random-effects models, which allows for between-trial variability.^41^ However, we replaced the prespecified frequentist model with fully bayesian random-effects models (eAppendix 1 in Supplement 1).

For binary outcomes, we used binomial likelihood and modeled the log RR directly. For continuous outcomes, we used the normal likelihood and the identity link. We assumed noninformative but biologically plausible priors for treatment effects. Details on the models, model diagnostics, and estimation methods are presented in eAppendix 11 in Supplement 1.

Summary treatment effect estimates and between-trial variance were derived from the median and 95% credibility intervals (CrIs) from the 2.5th and 97.5th percentile of the posterior distribution.

We conducted prespecified subgroup analyses for primary outcomes based on patient and drug characteristics and sensitive analyses based on the study’s methodological characteristics (eAppendix 12 in Supplement 1). We used prespecified frequentist fixed-effects meta-analysis models as a sensitivity analysis. For continuous outcomes, we used the inverse-variance model. For safety or immunogenicity outcomes, we used the Mantel-Haenszel method.

We investigated the association between trial size and treatment effects in contour-enhanced funnel plots for primary outcomes in the context of equivalence testing (eAppendix 13 and eFigure 1 in Supplement 1) and traditional funnel plots for the remaining outcomes (<10 studies). We also used Egger test for continuous outcomes and Harbord test for binary outcomes. Results with a *P* < .10 were considered statistically significant for Egger and Harbord tests.

We conducted nonprespecified trial sequential analyses based on large, randomized trials only to assess whether the combined number of analyzed participants was sufficient to draw definitive conclusions about the equivalence associated with biosimilars and reference biologics or whether more trials are still needed (eAppendix 14 in Supplement 1).^42^ We defined a large trial as a study that randomized at least 500 participants.^43^ We used this cutoff because large trials are less likely to be affected by small-study effects or publication bias.^43^ All analyses were conducted in MultiBUGS version 2.0 and Stata version 16 (StataCorp). *P* values were 2-sided for primary outcomes and 1-sided for secondary outcomes. Statistical significance was set at *P* < .05.

#### Margins of Equivalence

We considered equivalence as when the 95% CrI of summary estimates fell completely within the lower and upper prespecified equivalence margins.^37^ The rationale for the choice of the equivalence margins is described elsewhere.^37^ For the ACR20 outcome, we assumed an equivalence margin for RRs ranging from 0.94 to 1.06. For the HAQ-DI outcome, the equivalence margin in SMD units was −0.22 to 0.22. Thus, the probability of equivalence is the proportion of Markov Chain Monte Carlo simulations in which the random-effects summary estimate was within the equivalence margins. Importantly, the term *equivalence* used in this study refers to the statistical and clinical comparability of clinical responses between biosimilars and reference biologics and does not have the same value as the regulatory terms *biosimilarity*, *bioequivalence*, or *interchangeability*, provided by regulatory agencies, such as the US Food and Drug Administration.^44,45^

#### Assessment of Certainty of Evidence

We assessed the overall certainty of the evidence using the GRADE system^46^ (eAppendix 15 in Supplement 1). Data were analyzed from January 2022 to April 2023.

## Results

### Search Results

eFigure 2 in the Supplement summarizes the study selection process. Of 2023 references assessed, 25 RCTs^47,48,49,50,51,52,53,54,55,56,57,58,59,60,61,62,63,64,65,66,67,68,69,70,71,72,73,74,75,76,77,78,79,80,81,82,83,84,85,86,87,88,89,90,91,92,93,94^ met the eligible criteria.

### RCT Characteristics

The Table summarizes the characteristics of the 25 included trials, which included a total of 10 649 randomized participants, with a median (IQR) sample size of 426 (108 to 596) patients. The median (IQR) baseline age of the participants was 53 (51 to 54) years, and the median (IQR) proportion of females was 81% (80% to 84%).

**Table.** Main Characteristics of Included Trials and Patients at Baseline

Source	Biosimilar drug	Reference drug	Study design (efficacy phase)	Follow-up, wk	Randomized patients, No.	Age, mean (y)	Female patients, No. (%)
Jani et al, 2015^47^	ZRC-3197	ADA	Equivalence	12	120	45.0	99 (82.5)
Alten et al, 2017^48,49,50,51,52,53,54^	FKB327	ADA	Equivalence	24	730	53.3	565 (77.6)
Cohen et al, 2017^55,56^	ABP-501	ADA	Equivalence	26	526	55.9	426 (81.0)
Jamshidi et al, 2017^57^	CinnoRA	ADA	Noninferiority	24	136	47.9	118 (86.8)
Fleishmann et al, 2018^58,59,60^	PF-06410293	ADA	Equivalence	26	597	52.5	470 (78.7)
Cohen et al, 2018^61,62^	BI-695501	ADA	Equivalence	24	645	53.7	536 (83.1)
Weinblatt et al, 2018^63,64^	SB5	ADA	Equivalence	24	544	51.2	441 (81.1)
Edwards et al, 2019^65^	MSB11022	ADA	Superiority	52	288	54.0	227 (78.8)
Willand et al, 2019^66,67^	GP2017	ADA	Equivalence	24	353	53.3	295 (83.5)
Matsuno et al, 2021^68^^a^	LBAL	ADA	Equivalence	24	383	NA	NA
Kay et al, 2021^69,70^	CT-P17	ADA	Equivalence	24	648	53.8	514 (79.3)
Emery et al, 2015^71,72,73^	SB4	ETN	Equivalence	52	596	51.8	502 (84.2)
Bae et al, 2016^74^	HD203	ETN	Equivalence	48	294	51.2	202 (86.7)
Odell et al, 2016^75,76^^a^	CHS-0214	ETN	Equivalence	24	647	NA	514 (79.8)
Matsuno et al, 2017^77,78^	LBEC0101	ETN	Equivalence	54	374	54.1	316 (84.9)
Matucci-Cerinic et al, 2018^79,80^	GP2015	ETN	Equivalence	24	376	54.2	308 (81.9)
Yamanaka et al, 2020^81^	YLB113	ETN	Equivalence	56	528	52.3	409 (78.0)
Strusberg et al, 2021^82^	Enerceptan	ETN	Noninferiority	32	150	48.3	127 (85.2)
Yoo et al, 2013^83,84,85^	CT-P13	IFX	Equivalence	54	606	50.0	501 (82.7)
Kay et al, 2014^86,87^^a^	BOW015	IFX	Equivalence	16	189	NA	NA
Choe et al, 2015^88,89,90^	SB2	IFX	Equivalence	54	584	52.1	468 (80.1)
Takeuchi et al, 2015^91^	CT-P13	IFX	Equivalence	54	108	54.2	81 (80.2)
Matsuno et al, 2018^92^	NI071	IFX	Equivalence	30	242	53.9	204 (84.3)
Lila et al, 2019^93^	BCD-055	IFX	Equivalence	54	426	53.0	337 (80.6)
Genovese et al, 2020^94^	ABP710	IFX	Equivalence	50	558	54.9	437 (78.3)
Total	25	3	22 Equivalence, 2 noninferiority, 1 superiority	26 (24-52)^b^	426 (288-596)^b^	53.1 (51.3-54.0)^b^	81.1 (79.5-83.8)^b^

Abbreviations: ADA, adalimumab; ETN, etanercept; IFX, infliximab; NA, not available.

^a^ Unpublished trials in peer-reviewed journals (abstract congress).

^b^ Expressed as median (IQR).

All trials^47,48,49,50,51,52,53,54,55,56,57,58,59,60,61,62,63,64,65,66,67,68,69,70,71,72,73,74,75,76,77,78,79,80,81,82,83,84,85,86,87,88,89,90,91,92,93,94^ included patients with moderate to severe RA and experience with methotrexate. Sixteen (64%) trials^48,49,50,51,52,53,54,55,56,57,58,59,60,61,62,69,70,71,72,73,74,75,76,77,78,79,80,81,83,84,85,88,89,90,91,93^ reported the use of concomitant methotrexate in both treatment groups, while in 9 trials^47,63,64,65,66,67,68,82,86,87,92,94^ (36%), it was unclear. eTable 1 and eTable 2 in Supplement 1 provide additional information on the included trials. Overall, the most investigated biosimilars were those of adalimumab (11 trials^47,48,49,50,51,52,53,54,55,56,57,58,59,60,61,62,63,64,65,66,67,68,69,70^ [44%]), followed by biosimilars of etanercept^71,72,73,74,75,76,77,78,79,80,81,82^ and infliximab^83,84,85,86,87,88,89,90,91,92,93,94^ biosimilars, with 7 trials each (28%) (Table).

All 25 trials^47,48,49,50,51,52,53,54,55,56,57,58,59,60,61,62,63,64,65,66,67,68,69,70,71,72,73,74,75,76,77,78,79,80,81,82,83,84,85,86,87,88,89,90,91,92,93,94^ were industry-sponsored studies, and 3 trials^68,75,86^ (12%) were unpublished investigations. Most trials (22 trials^47,48,49,50,51,52,53,54,55,56,58,59,60,61,62,63,64,66,67,68,69,70,71,72,73,74,75,76,77,78,79,80,81,83,84,85,86,87,88,89,90,91,92,93,94^ [88%]) were equivalence trials, 2 trials^57,82^(8%) were noninferiority trials, and 1 trial^65^ (4%) was a superiority trial. The median (IQR) follow-up was 26 (24-52) weeks (Table). Additional methodological characteristics of the included trials are provided in eTable 3 and eTable 4 in Supplement 1.

### Risk of Bias

eFigure 3 in Supplement 1 shows the risk of bias in 25 trials.^47,48,49,50,51,52,53,54,55,56,57,58,59,60,61,62,63,64,65,66,67,68,69,70,71,72,73,74,75,76,77,78,79,80,81,82,83,84,85,86,87,88,89,90,91,92,93,94^ Fourteen trials^47,48,49,50,51,52,53,54,55,56,61,62,63,64,65,69,70,71,72,73,74,79,80,81,82,88,89,90,93^ (56%) had a low risk of bias for random sequence generation, 16 trials^48,49,50,51,52,53,54,55,56,57,59,60,61,62,63,64,65,66,67,68,69,70,71,72,73,74,77,78,79,80,81,82,88,89,90,92,93^ (64%) had a low risk of bias for allocation concealment, 19 trials^47,48,49,50,51,52,53,54,55,56,57,58,59,60,61,62,63,64,65,66,67,69,70,71,72,73,74,77,78,81,82,83,84,85,88,89,90,91,94^ (76%) had a low risk of bias for inconsistent application criteria, 12 trials^47,48,49,50,51,52,53,54,57,63,64,69,70,71,72,73,74,77,78,79,80,88,89,90,91,93^ (48%) had a low risk of bias for blinding of patients and investigators, 19 trials^47,48,49,50,51,52,53,54,55,56,57,58,59,60,61,62,63,64,65,69,70,74,77,78,79,80,81,82,83,84,85,88,89,90,91,92,94^ (76%) had a low risk of bias for patient’s behavior changes, 10 trials^63,64,69,70,71,72,73,74,77,78,79,80,82,88,89,90,91,93^ (40%) had a low risk of bias for blinding of outcome assessors, 22 trials^47,48,49,50,51,52,53,54,55,56,57,58,59,60,61,62,63,64,65,66,67,69,70,71,72,73,74,77,78,79,80,81,82,83,84,85,88,89,90,91,92,93,94^ (88%) had a low risk of bias for outcomes measures, and 8 trials^47,57,58,59,60,61,62,63,64,65,69,70,81^ (32%) had a low risk of bias for incomplete outcome data. Details on the risk of bias are provided in eTables 5-8 in Supplement 1.

### Primary Efficacy Outcomes

#### ACR20 After 6 Months of Treatment

**Figure 1.**
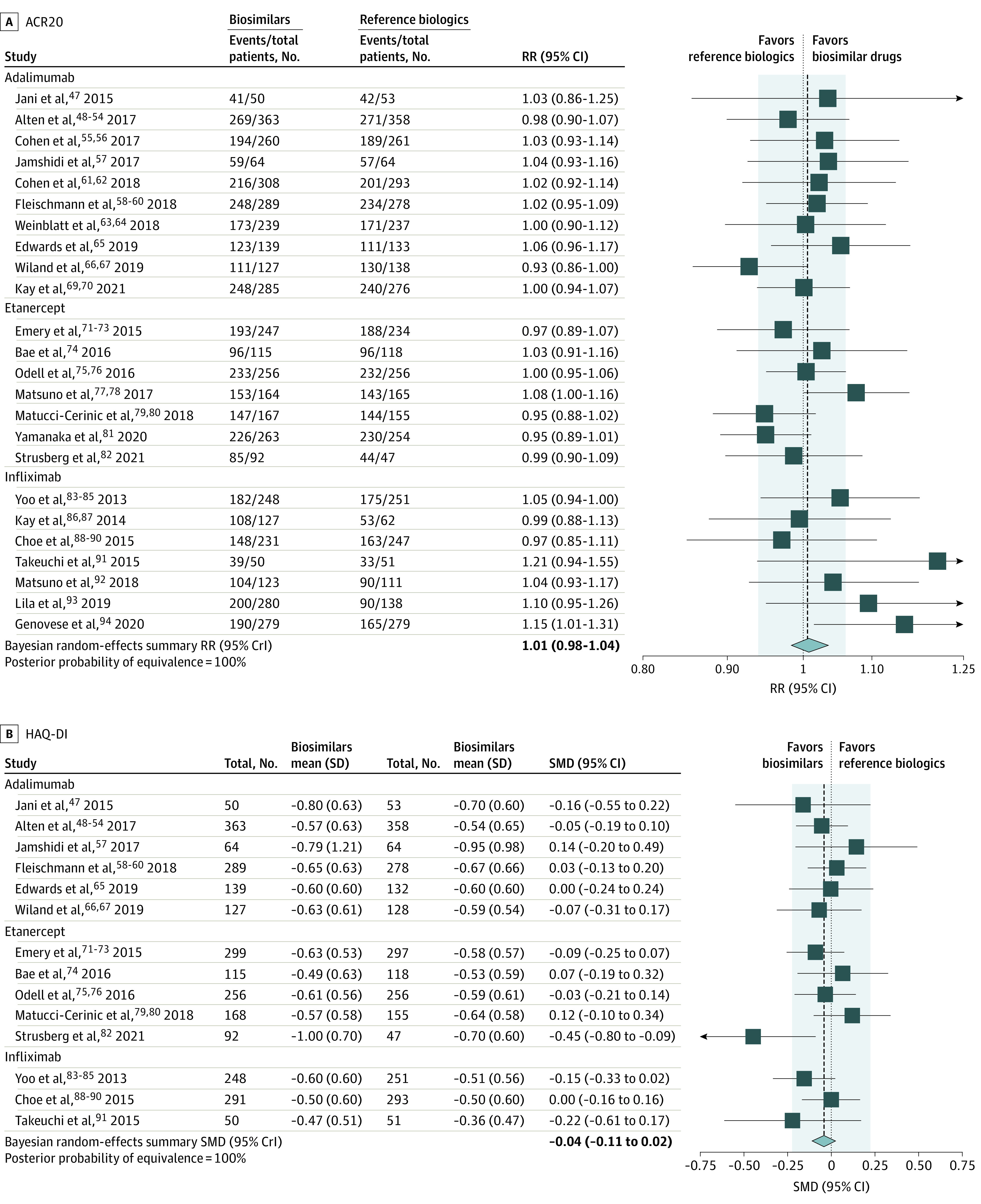
Forest Plots for American College of Rheumatology 20% Response Criteria (ACR20) and Health Assessment Questionnaire–Disability Index (HAQ-DI) at 6 Months After Treatment A, Meta-analysis of ACR20, including 24 trials comparing biosimilars vs reference biologic drugs (10 259 randomized patients). The shaded area denotes the margins of equivalence (relative risk [RR], 0.94 to 1.06). The estimate of the between-trial variance, τ^2^, was 0.000 (low heterogeneity). B, Meta-analysis of HAQ-DI, including 14 trials of biosimilars and reference biologic drugs (5579 randomized patients). The shaded area denotes the margins of equivalence (standardized mean difference [SMD], −0.22 to 0.22). The estimate between-trial variance, τ^2^, was 0.002 (low heterogeneity). Summary results are based on a bayesian random-effects model. Point estimates for primary studies are displayed with a 95% CI. Meta-analysis estimates are shown with a 95% credible interval (CrI). The number of participants analyzed may be smaller than the number of randomized participants.

A total of 24 trials^47,48,49,50,51,52,53,54,55,56,57,58,59,60,61,62,63,64,65,66,67,69,70,71,72,73,74,75,76,77,78,79,80,81,82,83,84,85,86,87,88,89,90,91,92,93,94^ involving 10 259 randomized patients contributed data for the primary outcome of ACR20 response at 6 months. As shown in Figure 1A, when data were combined, the bayesian random-effects summary was tiny (RR, 1.01; 95% Crl, 0.98 to 1.04), with no evidence of heterogeneity (τ^2^ = 0.000). The 95% bayesian CrI was entirely contained within the prespecified equivalence margin of 0.94 to 1.06 and met our prespecified definition of equivalence. The posterior probability of equivalence was 100% (eTable 9 in Supplement 1). Overall, 79% of patients receiving biosimilars and 78% of patients receiving reference biologics experienced an ACR20 response after 6 months of treatment. The prespecified summary estimate using a frequentist fixed-effect model indicated similar conclusions (eTable 10 in Supplement 1). Nonprespecified exploratory analyses that focused only on studies reporting intention-to-treat (ITT) analyses (eFigure 4 in Supplement 1) or PP analyses (eFigure 5 in Supplement 1) found identical conclusions compared with the main analysis.

#### HAQ-DI After 6 Months of Treatment

A total of 14 trials^47,48,49,50,51,52,53,54,57,58,59,60,65,66,67,71,72,73,74,75,76,79,80,82,83,84,85,88,89,90,91^ with 5579 randomized participants contributed data for HAQ-DI at 6 months. The bayesian random-effects summary SMD was −0.04 (95% CrI, −0.11 to 0.02), with low statistical heterogeneity (τ^2^ = 0.002) (Figure 1B), which falls entirely within the prespecified margins of equivalence of −0.22 to 0.22 (eTable 11 in Supplement 1). The summary SMD corresponds to a difference of −0.03 units (95% CrI, −0.08 to 0.01) on the HAQ-DI scale (range, 0-3). The prespecified sensitivity analysis using a frequentist fixed-effect model indicated analogous results (eTable 12 in Supplement 1). Exploratory analyses based on ITT studies only (eFigure 6 in Supplement 1) and PP analyses only (eFigure 7 in Supplement 1) found identical conclusions as the main analysis.

#### Subgroup Analysis for ACR20 and HAQ-DI

**Figure 2.**
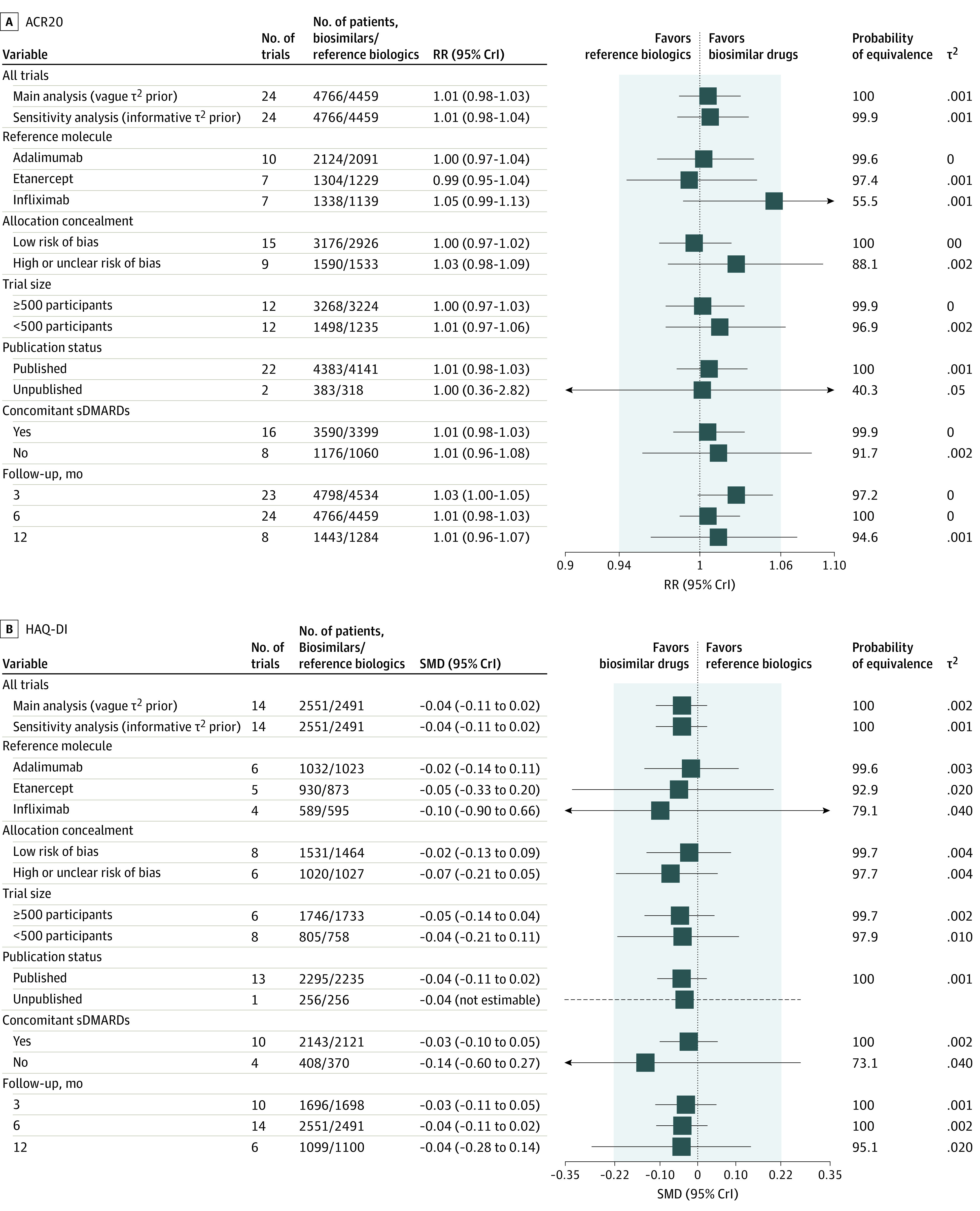
Subgroup Analysis for American College of Rheumatology 20% Response Criteria (ACR20) and Health Assessment Questionnaire–Disability Index (HAQ-DI) at 6 Months After Treatments A, Results are based on 24 trials comparing biosimilars vs reference biologic drugs (10 259 randomized patients). The shaded area denotes the margins of equivalence (relative risk [RR], 0.94 to 1.06). B, Results are based on 14 trials (5579 randomized patients). The shaded area denotes the margins of equivalence (standardized mean difference, [SMD], −0.22 to 0.22). Dashed horizontal lines represent credible intervals (CrI) that were not reliable (unrealistic large). sDMARDs indicates synthetic disease-modifying antirheumatic drugs.

The ACR20 response by subgroups yielded similar conclusions compared with the main analysis. In 8 of the 14 subgroups in Figure 2A, the 95% CrIs of the Bayesian summary RRs fell entirely within the predefined equivalence range, with low heterogeneity (τ^2^ ranging from 0 to 0.05). The subgroup analyses for HAQ-DI followed the same patterns, with the 95% CrIs of most subgroups within the equivalence margins, with posterior probabilities of equivalence between 73% to 100% (Figure 2B).

#### Trial Sequential Analysis Based on Large Trials: ACR20 and HAQ-DI

**Figure 3.**
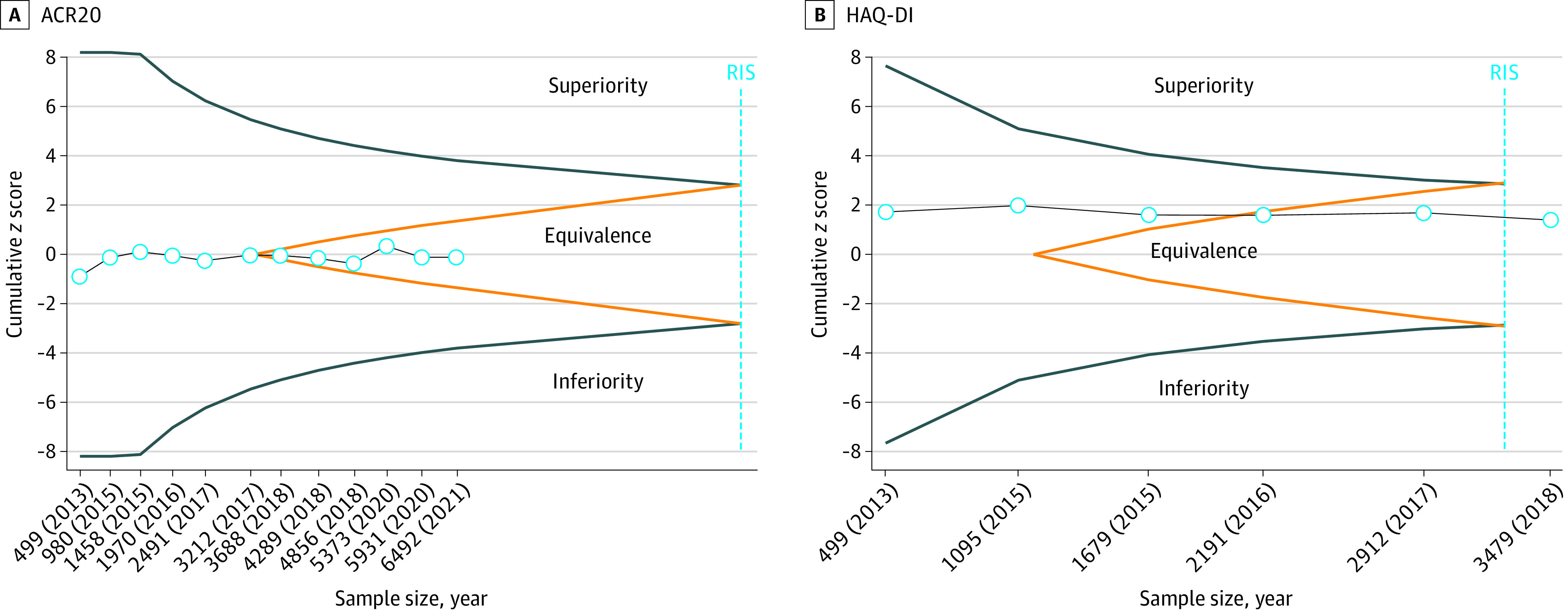
Trial Sequential Analyses for American College of Rheumatology 20% Response Criteria (ACR20) and Health Assessment Questionnaire–Disability Index (HAQ-DI) A, Results are based on 12 large, head-to-head trials of biosimilars and reference biologic drugs (7207 randomized participants). RIS indicates required information size (vertical line) to show equivalence considering the prespecified margins (relative risk [RR], 0.94 to 1.06]) with 90% of power at an α = .005. B, Results based on 6 large, head-to-head trials of biosimilars and reference biologic drugs for HAQ-DI (3758 randomized participants). The RIS was calculated as the sample size that gives a trial with 90% power to show equivalence (standardized mean difference [SMD] −0.22 to 0.22) with 2-sided α = .05. For A and B, cumulative *z* scores were estimated using a random-effects model with the restricted maximum likelihood estimator. The blue lines represent the O’Brien-Fleming monitoring boundaries. The orange characterizes the inner wedges (ie, futility boundaries), indicating the limits to the equivalence region. Circles indicate the cumulative *z* score for each additional trial added to the analysis. We assumed a between-trial variation using diversity (D2) index-adjusted sample sizes of 50%. The number of participants analyzed was shown by year and may be smaller than the number of randomized participants.

For the primary outcome of ACR20, trial sequential analysis based on 12 large trials^48,49,50,51,52,53,54,55,56,58,59,60,61,62,63,64,69,70,71,72,73,75,76,81,83,84,85,88,89,90,94^ with 7207 randomized participants demonstrated that the random-effects cumulative *Z *score crossed the boundary for equivalence in 2017, before the required information size was reached (Figure 3A). Additional trials after that year did not change the results, suggesting that the results are definitive. For the HAQ-DI, trial sequential analyses based on 6 large trials^48,49,50,51,52,53,54,58,59,60,71,72,73,75,76,83,84,85,88,89,90^ with 3758 randomized participants found that the accumulated number of participants reached the required information (eg, additional trials will not change the results). The random-effects *Z* score crossed the boundary of equivalence in 2016, indicating conclusive results (Figure 3B).

### Secondary Outcomes

#### Efficacy

Results for ACR50 and ACR70 responses are presented eTable 13 and eTable 14 in Supplement 1. Similar conclusions regarding the equivalence of biosimilars and reference biologics were obtained for these outcomes, consistent with the primary outcome.

#### Safety and Immunogenicity

Of 14 prespecified safety outcomes, 8 had sufficient data for meta-analysis. The assessments of all-cause mortality, mortality related to treatment, serious infections, active tuberculosis, and malignant neoplasms were broadly uninformative because of sparse data (eTable 15 in Supplement 1).

Overall, biosimilar drugs were associated with similar rates of serious adverse events, discontinuation, hypersensitivity, and NAbs compared with reference molecules (eTable 16 and eTable 17 in Supplement 1). However, the risks of TEAEs, injection site reactions (ISRs), and the formation of ADAs were lower in patients who received biosimilar drugs than those treated with reference biologic drugs. Overall, 35.9% of patients using biosimilars and 39.6% of patients using reference drugs experienced TEAEs, and 6.2% of patients using biosimilars and 19.9% of patients using reference drugs experienced ISRs. Regarding the immunogenicity profile, 30% of patients receiving biosimilars and 33.5% receiving reference biologics had test results positive for ADAs.

### Publication Bias and Small-Study Effects

**Figure 4.**
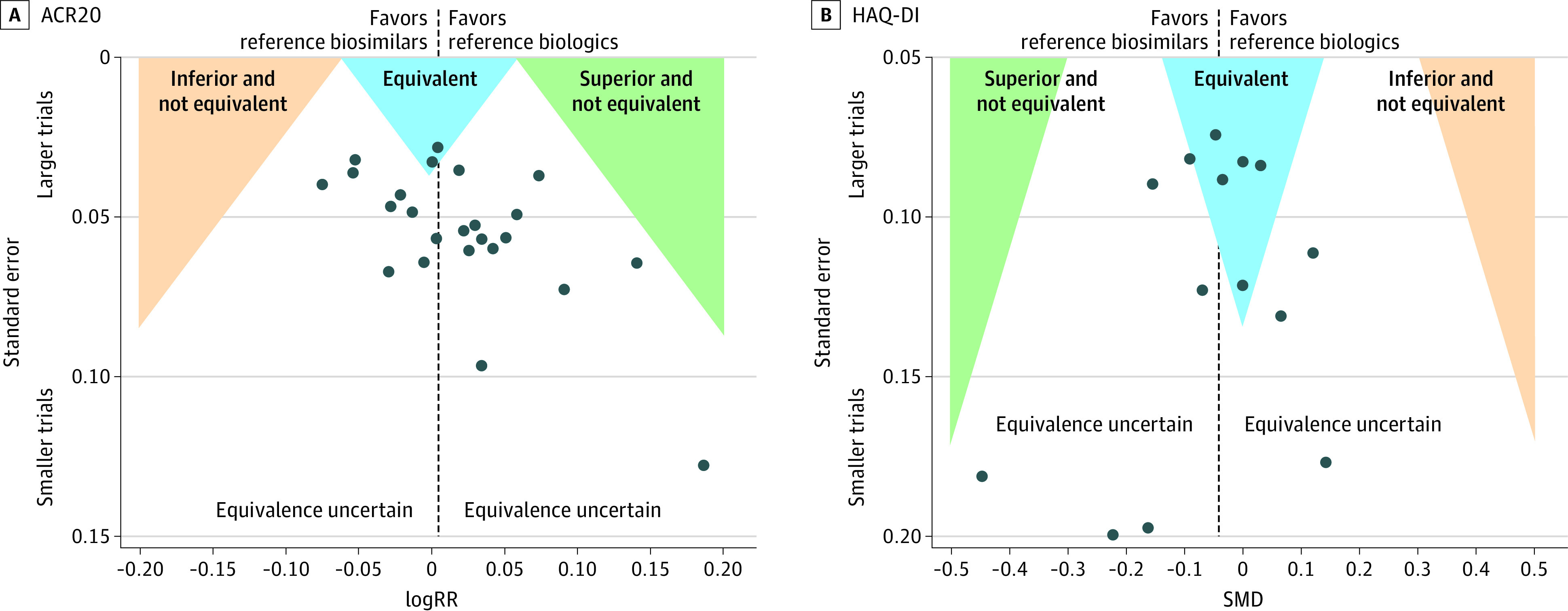
Contour-Enhanced Funnel Plots for American College of Rheumatology 20% Response Criteria (ACR20) and Health Assessment Questionnaire–Disability Index (HAQ-DI) A, Results based on 24 trials with 10 259 randomized participants. The dashed line indicates the log of summary estimates (relative risk [RR]) using a frequentist fixed-effects model (Mantel-Haenszel method). The enhanced areas show the 4 major statistical regions pertinent to equivalence testing: equivalence uncertain, inferior and not equivalent, equivalent, and superior and not equivalent. Harbord test was statistically significant (*P* = .02). B, Results based on 14 trials with 5579 randomized patients. The dashed line represents the summary estimates (standardized mean difference [SMD]) using a frequentist fixed-effects model. The enhanced areas show the 4 major statistical regions pertinent to equivalence testing: equivalence uncertain, inferior and not equivalent, equivalent, and superior and not equivalent. The Egger test was not statistically significant (*P* = .39). Smaller trials indicates trials with fewer than 500 participants randomized; larger trials, at least 500 participants randomized.

Figure 4A shows that the funnel plot for ACR20 (24 trials^47,48,49,50,51,52,53,54,55,56,57,58,59,60,61,62,63,64,65,66,67,69,70,71,72,73,74,75,76,77,78,79,80,81,82,83,84,85,86,87,88,89,90,91,92,93,94^) was slightly asymmetric (Harbord test: *P* = .02), indicating the possibility of small-study bias toward the suppression of small trials with inconclusive equivalence results. No evidence of funnel plot asymmetry was observed for HAQ-DI (14 trials^47,48,49,50,51,52,53,54,57,58,59,60,65,66,67,71,72,73,74,75,76,79,80,82,83,84,85,88,89,90,91^) (Egger test: *P* = .39) (Figure 4B). Because of funnel plot asymmetry, we conducted a nonprespecified analysis restricted to large trials (≥500 randomized participants) to mitigate the possibility of small-study bias. Results based on 12 large trials^48,49,50,51,52,53,54,55,56,58,59,60,61,62,63,64,69,70,71,72,73,75,76,81,83,84,85,88,89,90,94^ (7207 participants) provided virtually identical conclusions on ACR20 (RR, 1.00; 95% Crl, 0.98 to 1.03; τ^2^ = 0) with a posterior probability of equivalence of 100%. Results of a nonprespecified analysis based on 6 large trials^48,49,50,51,52,53,54,58,59,60,71,72,73,75,76,83,84,85,88,89,90^ (3758 participants) provided virtually identical conclusions on HAQ-DI scores (SMD, −0.05; 95% Crl, −0.14 to 0.04; τ^2^ = 0.002) with a posterior probability of equivalence of 99.8%. The visual inspection of the funnel plot for the risk of ISRs and ADAs also revealed suspected asymmetry of the funnel plot and was confirmed by Harbord tests (eFigures 8-15 in Supplement 1).

### Overall Certainty of the Evidence

eTable 18 in the Supplement summarizes the certainty of the evidence of each outcome as the reasons for downgrading the evidence. The evidence suggested that biosimilars were not associated with differences in ACR20 response, HAQ-DI, ACR50 response, or ACR70 response (moderate certainty). There was moderate certainty of evidence that biosimilars likely resulted in little to no difference for safety outcomes assessed, except for TEAE, ISRs, and ADAs and NAbs. For these, the evidence was rated as low certainty.

## Discussion

This systematic review and meta-analysis identified 25 head-to-head trials^47,48,49,50,51,52,53,54,55,56,57,58,59,60,61,62,63,64,65,66,67,68,69,70,71,72,73,74,75,76,77,78,79,80,81,82,83,84,85,86,87,88,89,90,91,92,93,94^ (including 10 642 randomized participants) that compared the effects of biosimilars of adalimumab, etanercept, and infliximab vs their reference biologic drugs in patients with RA. Summary estimates met the prespecified criteria for equivalence based on 24 trials^47,48,49,50,51,52,53,54,55,56,57,58,59,60,61,62,63,64,65,66,67,69,70,71,72,73,74,75,76,77,78,79,80,81,82,83,84,85,86,87,88,89,90,91,92,93,94^ for ACR20 and 14 trials^47,48,49,50,51,52,53,54,57,58,59,60,65,66,67,71,72,73,74,75,76,79,80,82,83,84,85,88,89,90,91^ for HAQ-DI. The robustness of the results was confirmed through subgroup analyses and trial sequential analyses. Moreover, biosimilars were associated with similar rates of adverse events, study discontinuation, and immunogenicity responses compared with reference biologics.

To our knowledge, this is the largest systematic review to adequately examine the equivalence of biosimilars and reference biologics in RA.^30^ Only 4 systematic reviews^20,25,28,29^ of head-to-head trials comparing biosimilars and reference biologics in patients with RA have been published. As these reviews ignored the equivalence or noninferiority design of the primary studies, aiming to determine whether biosimilar drugs were superior to their reference biologics, our results are not directly comparable. As opposed to previous reviews, our review shows up-to-date conclusive evidence on the equivalence between biosimilars and their reference biologics via bayesian meta-analysis using prespecified margins of equivalence and trial sequential analysis. Overall, our results are in line with the conclusions that both biosimilars and reference biologics are equally valuable for RA treatment.

### Limitations

Our review has important limitations. First, we did not assess biosimilars of all reference biologics currently available for RA treatment on the market. Thus, our findings may have limited generalizability beyond the 3 reference biologics that were investigated. Future initiatives addressing the equivalence of biosimilars and different reference biologics are needed. Second, our prespecified subgroup analyses, especially those by reference biologics, had few available trials, with some yielding point estimates with considerable uncertainty. Therefore, our findings characterize the equivalence between biosimilars and reference biologics as a combined group (adalimumab, etanercept, and infliximab together). Additional meta-analyses considering each reference molecule separately are warranted as more trials are performed and published, especially for etanercept biosimilars. Third, safety data remain sparse for some outcomes, and more primary investigations with large sample sizes are still required. Fourth, our results regarding the equivalence between biosimilars and reference biologics are valid for treatments without switching (ie, the same intervention is given from the beginning to the end of follow-up). The association of switching with clinical response and safety outcomes should be a topic of additional systematic reviews.^37^ Fifth, we detected funnel plot asymmetry for ACR20 and implemented secondary analyses based on large trials to address potential biases related to small-study effects. Based on a previous empirical investigation, we defined a large trial as having more than 500 randomized participants.^43^ We recognize that this criterion may not directly apply to RA, a relatively rare condition. If anything, our approach is conservative, because trials of this size are more likely to be published, regardless of their results. Sixth, assessing publication and small-study biases is not straightforward in a meta-analysis of equivalence trials. Traditional methods of funnel plot assessment rely on superiority testing,^95,96^ which may not be optimal for equivalence testing. While our approach of constructing contour-enhanced funnel plots is one option, assuming a common equivalence margin for all trials may not be ideal. Further research is warranted to investigate the best strategies to address publication and small-study bias in meta-analyses involving equivalence or noninferiority trials.

## Conclusions

This systematic review and meta-analysis found that there was compelling evidence of equivalence between adalimumab, infliximab, and etanercept biosimilars and their originators, based on ACR20 (a clinician-assessed outcome) and HAQ-DI (a patient-reported outcome). The findings support the rational use of these biosimilars for RA treatment.

## Notes

Supplement 1. **eAppendix 1.** Changes to the Protocol with Reasons **eAppendix 2.** Detailed Description of the Eligibility Criteria and Study Selection **eAppendix 3.** Evidence Sources and Search Strategy **eAppendix 4.** Screening and Selection Process **eAppendix 5.** Data Collection Process **eAppendix 6.** Prespecified Coprimary Outcomes of Efficacy **eAppendix 7.** Prespecified Secondary Outcomes of Efficacy **eAppendix 8.** Prespecified Outcomes of Safety and Immunogenicity **eAppendix 9.** Risk of Bias Assessment **eAppendix 10.** Approximate Bayesian Computation Model and Other Approximations **eAppendix 11.** Bayesian Model Fitting, Model Diagnostics, and Estimation Methods **eAppendix 12.** Subgroup and Sensitive Analysis **eAppendix 13.** Contour-Enhanced Plots for Coprimary Outcomes **eFigure 1.** Possible Scenarios Within an Equivalence Testing **eAppendix 14.** Trial Sequential Analysis Based on Large, Randomized Trials **eAppendix 15.** Certainty of Evidence Assessment: GRADE Approach **eFigure 2.** Flow Diagram of Evidence, and Main Reasons for Exclusions **eTable 1.** Demographic and Clinical Characteristics of Participants at Baseline Per Study Arm **eTable 2.** Main and Adjuvant Therapy Schemes of Reference Biologics and Biosimilars From Included Trials **eTable 3.** Study Characteristics of Included Randomized Trials **eTable 4.** Margins of Equivalence, Noninferiority, or Superiority in Trials of Biosimilars and Reference Biologic Drugs, and Other Statistical Information for their Calculation **eFigure 3.** The Risk of Bias of Included Studies **eTable 5.** Risk of Bias Assessment Domains: Random Sequence Generation, Allocation Concealment, and Inconsistent Application of Eligibility Criteria **eTable 6.** Risk of Bias Assessment Domains: Blinding of Participants and Investigator, and Participant’s Behaviour Changes **eTable 7.** Risk of Bias Assessment Domains: Blinding of Outcome Assessors, and Outcomes Measures **eTable 8.** Risk of Bias Assessment Domain: Incomplete Outcome Data **eTable 9.** Effects of Biosimilars and Biologics on ACR20 Response: Bayesian Random-Effects Meta-analysis **eTable 10.** Effects of Biosimilars and Biologics on ACR20 Response: Frequentist Fixed-Effects Meta-analysis **eFigure 4.** Nonprespecified and Exploratory Analyses of ACR20 Including Only Studies Reporting Modified Intention-to-Treat or Intention-to-Treat Analyses **eFigure 5.** Nonprespecified and Exploratory Analyses of ACR20 Including Only Studies Reporting Per-Protocol Analyses **eTable 11.** Effects of Biosimilars and Biologics on HAQ-DI: Bayesian Random-Effects Meta-analysis **eTable 12.** Effects of Biosimilars and Biologics on HAQ-DI: Frequentist Fixed-Effects Meta-analysis **eFigure 6.** Nonprespecified and Exploratory Analyses of HAQ-DI Including Only Studies Reporting Modified Intention-to-Treat or Intention-to-Treat Analyses **eFigure 7.** Nonprespecified and Exploratory Analyses of ACR20 Including Only Studies Reporting Per-Protocol Analyses **eTable 13.** Effects of Biosimilars and Biologics on ACR50 and ACR70: Bayesian Random-Effects Meta-analysis **eTable 14.** Effects of Biosimilars and Biologics on ACR50 and ACR70: Frequentist Fixed-Effects Meta-analysis **eTable 15.** Frequency of Safety Outcomes With Uncommon Events (Meta-analysis Not Performed) **eTable 16.** Effects of Biosimilars and Reference Biologics on Safety and Immunogenicity Outcomes (Bayesian Random-Effects Meta-analysis) **eTable 17.** Effects of Biosimilars and Reference Biologics on Safety and Immunogenicity Outcomes (Frequentist Fixed-Effects Meta-analysis) **eFigure 8.** Funnel Plot for the Effects of Biosimilars Group vs References Group on ACR50 at 6 Months of Follow-up **eFigure 9.** Funnel Plot for the Effects of Biosimilars Group vs References Group on ACR70 at 6 Months of Follow-up **eFigure 10.** Funnel Plot for the Effects of Biosimilars Group vs References Group on the Risk of Overall Treatment-Emergent Adverse Effects **eFigure 11.** Funnel Plot for the Effects of Biosimilars Group vs References Group on the Risk of Serious Treatment-Emergent Adverse Effects **eFigure 12.** Funnel Plot for the Effects of Biosimilars Group vs References Group on the Risk of Injection Site Reactions **eFigure 13.** Funnel Plot for the Effects of Biosimilars Group vs References Group on the Risk of Overall Discontinuation Rates **eFigure 14.** Funnel Plot for the Effects of Biosimilars Group vs References Group on the Risk of Positive Antidrug Antibodies **eFigure 15.** Funnel Plot for the Effects of Biosimilars Group vs References Group on the Risk of Positive Neutralizing Antibodies **eTable 18.** Certainty of Evidence Assessment of Trials Comparing Biosimilars vs Their Reference Biologic Drugs in Patients With Arthritis Rheumatoid (GRADE Evidence Profile)
eReferences.

Supplement 2.
Data Sharing Statement

## References

[1] Aletaha D, Smolen JS. Diagnosis and management of rheumatoid arthritis: a review. JAMA. 2018;320(13):1360-1372. doi:10.1001/jama.2018.13103 30285183

[2] Safiri S, Kolahi AA, Hoy D, . Global, regional and national burden of rheumatoid arthritis 1990-2017: a systematic analysis of the Global Burden of Disease study 2017. Ann Rheum Dis. 2019;78(11):1463-1471. doi:10.1136/annrheumdis-2019-215920 31511227

[3] Stevenson M, Archer R, Tosh J, . Adalimumab, etanercept, infliximab, certolizumab pegol, golimumab, tocilizumab and abatacept for the treatment of rheumatoid arthritis not previously treated with disease-modifying antirheumatic drugs and after the failure of conventional disease-modifying antirheumatic drugs only: systematic review and economic evaluation. Health Technol Assess. 2016;20(35):1-610. doi:10.3310/hta20350 27140438 PMC4867425

[4] Janke K, Biester K, Krause D, . Comparative effectiveness of biological medicines in rheumatoid arthritis: systematic review and network meta-analysis including aggregate results from reanalysed individual patient data. BMJ. 2020;370:m2288. doi:10.1136/bmj.m2288 32636183 PMC7338922

[5] Fraenkel L, Bathon JM, England BR, . 2021 American College of Rheumatology guideline for the treatment of rheumatoid arthritis. Arthritis Rheumatol. 2021;73(7):1108-1123. doi:10.1002/art.41752 34101376

[6] Smolen JS, Landewé RBM, Bergstra SA, . EULAR recommendations for the management of rheumatoid arthritis with synthetic and biological disease-modifying antirheumatic drugs: 2022 update. Ann Rheum Dis. 2023;82(1):3-18. doi:10.1136/ard-2022-223356 36357155

[7] World Health Organization. WHO guideline on country pharmaceutical pricing policies, second edition. Accessed November 10, 2022. https://apps.who.int/iris/bitstream/handle/10665/335692/9789240011878-eng.pdf

[8] Kay J, Schoels MM, Dörner T, ; Task Force on the Use of Biosimilars to Treat Rheumatological Diseases. Consensus-based recommendations for the use of biosimilars to treat rheumatological diseases. Ann Rheum Dis. 2018;77(2):165-174. doi:10.1136/annrheumdis-2017-211937 28866648

[9] Hsieh PH, Wu O, Geue C, McIntosh E, McInnes IB, Siebert S. Economic burden of rheumatoid arthritis: a systematic review of literature in biologic era. Ann Rheum Dis. 2020;79(6):771-777. doi:10.1136/annrheumdis-2019-216243 32245893

[10] Kim H, Alten R, Avedano L, . The future of biosimilars: maximizing benefits across immune-mediated inflammatory diseases. Drugs. 2020;80(2):99-113. doi:10.1007/s40265-020-01256-5 32002851 PMC7007415

[11] Vogler S, Schneider P, Zuba M, Busse R, Panteli D. Policies to encourage the use of biosimilars in european countries and their potential impact on pharmaceutical expenditure. Front Pharmacol. 2021;12:625296. doi:10.3389/fphar.2021.625296 34248615 PMC8267415

[12] Kvien TK, Patel K, Strand V. The cost savings of biosimilars can help increase patient access and lift the financial burden of health care systems. Semin Arthritis Rheum. 2022;52:151939. doi:10.1016/j.semarthrit.2021.11.009 35027243

[13] Bridges SL Jr, White DW, Worthing AB, ; American College of Rheumatology. The science behind biosimilars: entering a new era of biologic therapy. Arthritis Rheumatol. 2018;70(3):334-344. doi:10.1002/art.40388 29411547

[14] Mota LMHD, Kakehasi AM, Gomides APM, . 2017 Recommendations of the Brazilian Society of Rheumatology for the pharmacological treatment of rheumatoid arthritis. Adv Rheumatol. 2018;58(1):2. doi:10.1186/s42358-018-0005-0 30657071

[15] Ho CTK, Mok CC, Cheung TT, Kwok KY, Yip RML; Hong Kong Society of Rheumatology. Management of rheumatoid arthritis: 2019 updated consensus recommendations from the Hong Kong Society of Rheumatology. Clin Rheumatol. 2019;38(12):3331-3350. doi:10.1007/s10067-019-04761-5 31485846

[16] Parisi S, Bortoluzzi A, Sebastiani GD, . The Italian Society for Rheumatology clinical practice guidelines for rheumatoid arthritis. Reumatismo. 2019;71(S1):22-49. doi:10.4081/reumatismo.2019.1202 31948192

[17] Kawahito Y, Morinobu A, Kaneko Y, . Drug treatment algorithm and recommendations from the 2020 update of the Japan College of Rheumatology clinical practice guidelines for the management of rheumatoid arthritis-secondary publication. Mod Rheumatol. 2023;33(1):21-35. doi:10.1093/mr/roac017 35297492

[18] Kerschbaumer A, Sepriano A, Bergstra SA, . Efficacy of synthetic and biological DMARDs: a systematic literature review informing the 2022 update of the EULAR recommendations for the management of rheumatoid arthritis. Ann Rheum Dis. 2023;82(1):95-106. doi:10.1136/ard-2022-223365 36368906

[19] Chingcuanco F, Segal JB, Kim SC, Alexander GC. Bioequivalence of biosimilar tumor necrosis factor-α inhibitors compared with their reference biologics: a systematic review. Ann Intern Med. 2016;165(8):565-574. doi:10.7326/M16-0428 27479870

[20] Komaki Y, Yamada A, Komaki F, . Efficacy, safety and pharmacokinetics of biosimilars of anti-tumor necrosis factor-α agents in rheumatic diseases: a systematic review and meta-analysis. J Autoimmun. 2017;79:4-16. doi:10.1016/j.jaut.2017.02.003 28209290

[21] Moots R, Azevedo V, Coindreau JL, . Switching between reference biologics and biosimilars for the treatment of rheumatology, gastroenterology, and dermatology inflammatory conditions: considerations for the clinician. Curr Rheumatol Rep. 2017;19(6):37. doi:10.1007/s11926-017-0658-4 28623625 PMC5486595

[22] Graudal N, Kaas-Hansen BS, Guski L, Hubeck-Graudal T, Welton NJ, Jürgens G. Different original and biosimilar TNF inhibitors similarly reduce joint destruction in rheumatoid arthritis—a network meta-analysis of 36 randomized controlled trials. Int J Mol Sci. 2019;20(18):4350. doi:10.3390/ijms20184350 31491879 PMC6770755

[23] Huizinga TW, Torii Y, Muniz R. Adalimumab biosimilars in the treatment of rheumatoid arthritis: a systematic review of the evidence for biosimilarity. Rheumatol Ther. 2021;8(1):41-61. doi:10.1007/s40744-020-00259-8 33263165 PMC7991039

[24] Kerschbaumer A, Sepriano A, Smolen JS, . Efficacy of pharmacological treatment in rheumatoid arthritis: a systematic literature research informing the 2019 update of the EULAR recommendations for management of rheumatoid arthritis. Ann Rheum Dis. 2020;79(6):744-759. doi:10.1136/annrheumdis-2019-216656 32033937 PMC7286044

[25] Ho Lee Y, Gyu Song G. Comparative efficacy and safety of tumor necrosis factor inhibitors and their biosimilars in patients with rheumatoid arthritis having an insufficient response to methotrexate: a network meta-analysis. Z Rheumatol. 2023;82(3):248-255. doi:10.1007/s00393-021-01041-z 34223982

[26] Hanrahan C, Lee T. Network meta-analysis of infliximab biosimilars for the treatment of rheumatoid arthritis. Am J Health Syst Pharm. 2021;78(8):697-704. doi:10.1093/ajhp/zxab042 33599738 PMC7929383

[27] Horta-Baas G. Patient-reported outcomes in rheumatoid arthritis: a key consideration for evaluating biosimilar uptake? Patient Relat Outcome Meas. 2022;13:79-95. doi:10.2147/PROM.S256715 35388274 PMC8977480

[28] Tanaka E, Kawahito Y, Kohno M, . Systematic review and meta-analysis of biosimilar for the treatment of rheumatoid arthritis informing the 2020 update of the Japan College of Rheumatology clinical practice guidelines for the management of rheumatoid arthritis. Mod Rheumatol. 2022;32(1):74-86. doi:10.1080/14397595.2021.1899591 33706664

[29] Hu R, Yuan T, Wang H, . Efficacy, safety and immunogenicity of etanercept biosimilars *versus* reference biologics in patients with rheumatoid arthritis: A meta-analysis. Front Pharmacol. 2023;14:1089272. doi:10.3389/fphar.2023.1089272 36874005 PMC9979087

[30] Acuna SA, Dossa F, Baxter N. Meta-analysis of noninferiority and equivalence trials: ignoring trial design leads to differing and possibly misleading conclusions. J Clin Epidemiol. 2020;127:134-141. doi:10.1016/j.jclinepi.2020.05.034 32540386

[31] Fisher A, Bassett K, Wright JM, Brookhart MA, Freeman H, Dormuth CR. Comparative persistence of the TNF antagonists in rheumatoid arthritis–a population-based cohort study. PLoS One. 2014;9(8):e105193. doi:10.1371/journal.pone.0105193 25141123 PMC4139324

[32] Silvagni E, Bortoluzzi A, Carrara G, Zanetti A, Govoni M, Scirè CA. Comparative effectiveness of first-line biological monotherapy use in rheumatoid arthritis: a retrospective analysis of the Record-Linkage on Rheumatic Diseases study on health care administrative databases. BMJ Open. 2018;8(9):e021447. doi:10.1136/bmjopen-2017-021447 30206082 PMC6144331

[33] Kearsley-Fleet L, Davies R, De Cock D, ; BSRBR-RA Contributors Group. Biologic refractory disease in rheumatoid arthritis: results from the British Society for Rheumatology Biologics Register for Rheumatoid Arthritis. Ann Rheum Dis. 2018;77(10):1405-1412. doi:10.1136/annrheumdis-2018-213378 29980575 PMC6161665

[34] Sullivan E, Kershaw J, Blackburn S, Mahajan P, Boklage SH. Biologic disease-modifying antirheumatic drug prescription patterns among rheumatologists in Europe and Japan. Rheumatol Ther. 2020;7(3):517-535. doi:10.1007/s40744-020-00211-w 32440826 PMC7410899

[35] Sullivan E, Kershaw J, Blackburn S, Choi J, Curtis JR, Boklage S. Biologic disease-modifying antirheumatic drug prescription patterns for rheumatoid arthritis among United States physicians. Rheumatol Ther. 2020;7(2):383-400. doi:10.1007/s40744-020-00203-w 32318979 PMC7211222

[36] Finckh A, Tellenbach C, Herzog L, ; physicians and patients of the SCQM. Comparative effectiveness of antitumour necrosis factor agents, biologics with an alternative mode of action and tofacitinib in an observational cohort of patients with rheumatoid arthritis in Switzerland. RMD Open. 2020;6(1):e001174. doi:10.1136/rmdopen-2020-001174 32385143 PMC7299517

[37] Ascef BO, Almeida MO, de Medeiros Ribeiro AC, . Equivalence and switching between biosimilars and reference molecules in rheumatoid arthritis: protocol for a systematic review and meta-analysis. Syst Rev. 2021;10(1):205. doi:10.1186/s13643-021-01754-x 34274019 PMC8286602

[38] Page MJ, McKenzie JE, Bossuyt PM, . The PRISMA 2020 statement: an updated guideline for reporting systematic reviews. BMJ. 2021;372(160):n71. doi:10.1136/bmj.n71 33782057 PMC8005924

[39] Treadwell JR, Uhl S, Tipton K, . Assessing equivalence and noninferiority. J Clin Epidemiol. 2012;65(11):1144-1149. doi:10.1016/j.jclinepi.2012.05.001 22732455

[40] Higgins JPT, Green S, eds. Cochrane Handbook for Systematic Reviews of Interventions Version 5.1.0. Accessed March 23, 2023. https://training.cochrane.org/handbook/archive/v5.1/

[41] Borenstein M, Hedges LV, Higgins JP, Rothstein HR. A basic introduction to fixed-effect and random-effects models for meta-analysis. Res Synth Methods. 2010;1(2):97-111. doi:10.1002/jrsm.12 26061376

[42] Wetterslev J, Thorlund K, Brok J, Gluud C. Trial sequential analysis may establish when firm evidence is reached in cumulative meta-analysis. J Clin Epidemiol. 2008;61(1):64-75. doi:10.1016/j.jclinepi.2007.03.013 18083463

[43] Jones CW, Handler L, Crowell KE, Keil LG, Weaver MA, Platts-Mills TF. Non-publication of large randomized clinical trials: cross sectional analysis. BMJ. 2013;347:f6104. doi:10.1136/bmj.f6104 24169943 PMC3812466

[44] US Food and Drug Administration. New and revised draft Q&As on biosimilar development and the BPCI Act (Revision 3). Accessed March 23, 2023. https://www.fda.gov/media/119278/download

[45] McKinnon RA, Cook M, Liauw W, . Biosimilarity and interchangeability: principles and evidence: a systematic review. BioDrugs. 2018;32(1):27-52. doi:10.1007/s40259-017-0256-z 29344876 PMC5814534

[46] Schünemann H, Brożek J, Guyatt G, Oxman A, eds. GRADE handbook for grading quality of evidence and strength of recommendations: the GRADE Working Group. Accessed March 23, 2023. https://gdt.gradepro.org/app/handbook/handbook.html#h.1i2bwkm8zpjo

[47] Jani RH, Gupta R, Bhatia G, . A prospective, randomized, double-blind, multicentre, parallel-group, active controlled study to compare efficacy and safety of biosimilar adalimumab (Exemptia; ZRC-3197) and adalimumab (Humira) in patients with rheumatoid arthritis. Int J Rheum Dis. 2016;19(11):1157-1168. doi:10.1111/1756-185X.12711 26176644 PMC5215647

[48] Alten R, Glover J, Matsunaga N, Chisholm D, Genovese M. OP0021: efficacy and safety results of a phase iii study comparing fkb327, an adalimumab biosimilar, with the adalimumab reference product in patients with active rheumatoid arthritis. Ann Rheum Dis. 2017;76:59. doi:10.1136/annrheumdis-2017-eular.2220

[49] Genovese MC, Glover J, Matsunaga N, Chisholm D, Alten R. 2799 Efficacy, safety and immunogenicity in randomized, double-blind (DB) and openlabel extension (OLE) studies comparing FKB327, an adalimumab biosimilar, with the adalimumab reference product (Humira; RP) in Patients (pts) with active rheumatoid arthritis (RA). Arthritis Rheumatol. 2017;69(suppl 10).

[50] Genovese MC, Kellner H, Arai Y, Muniz R, Alten R. Long-term safety, immunogenicity and efficacy comparing FKB327 with the adalimumab reference product in patients with active rheumatoid arthritis: data from randomised double-blind and open-label extension studies. RMD Open. 2020;6(1):e000987. doi:10.1136/rmdopen-2019-000987 32371430 PMC7299509

[51] Alten R, Markland C, Kawakami K, Boyce M CF, Muniz R, Genovese MC. P422 immunogenicity of a proposed adalimumab biosimilar, FKB327, and the reference product in patients with rheumatoid arthritis. J Crohn Colitis. 2019;13(Supplement 1):S320. doi:10.1093/ecco-jcc/jjy222.546 PMC7754138 32852139

[52] Genovese MC, Glover J, Greenwald M, . FKB327, an adalimumab biosimilar, versus the reference product: results of a randomized, Phase III, double-blind study, and its open-label extension. Arthritis Res Ther. 2019;21(1):281. doi:10.1186/s13075-019-2046-0 31831079 PMC6909638

[53] Alten R, Markland C, Boyce M, Kawakami K, Muniz R, Genovese MC. Immunogenicity of an adalimumab biosimilar, FKB327, and its reference product in patients with rheumatoid arthritis. Int J Rheum Dis. 2020;23(11):1514-1525. doi:10.1111/1756-185X.13951 32852139 PMC7754138

[54] Genovese MC, Kellner H, Arai Y, Muniz R, Alten R. Long-term safety, immunogenicity and efficacy comparing FKB327 with the adalimumab reference product in patients with active rheumatoid arthritis: data from randomised double-blind and open-label extension studies. RMD Open. 2020;6(1):e000987. doi:10.1136/rmdopen-2019-000987 32371430 PMC7299509

[55] Cohen S, Genovese MC, Choy E, . Efficacy and safety of the biosimilar ABP 501 compared with adalimumab in patients with moderate to severe rheumatoid arthritis: a randomised, double-blind, phase III equivalence study. Ann Rheum Dis. 2017;76(10):1679-1687. doi:10.1136/annrheumdis-2016-210459 28584187 PMC5629940

[56] Cohen S, Pablos JL, Pavelka K, . An open-label extension study to demonstrate long-term safety and efficacy of ABP 501 in patients with rheumatoid arthritis. Arthritis Res Ther. 2019;21(1):84. doi:10.1186/s13075-019-1857-3 30922373 PMC6440148

[57] Jamshidi A, Gharibdoost F, Vojdanian M, . A phase III, randomized, two-armed, double-blind, parallel, active controlled, and non-inferiority clinical trial to compare efficacy and safety of biosimilar adalimumab (CinnoRA) to the reference product (Humira) in patients with active rheumatoid arthritis. Arthritis Res Ther. 2017;19(1):168. doi:10.1186/s13075-017-1371-4 28728599 PMC5520357

[58] Fleischmann R, Alvarez D, Bock A, A randomized, double-blind phase 3 study comparing the efficacy, safety and immunogenicity of PF-06410293 (Abrilada), an adalimumab (ADL) biosimilar, and reference ADL (Humira) in patients with moderate to severe active RA: results from weeks 52-92. Arthritis Rheumatol. 2020; 72(suppl 10).

[59] Fleischmann RM, Alten R, Pileckyte M, . A comparative clinical study of PF-06410293, a candidate adalimumab biosimilar, and adalimumab reference product (Humira) in the treatment of active rheumatoid arthritis. Arthritis Res Ther. 2018;20(1):178. doi:10.1186/s13075-018-1676-y 30111357 PMC6094896

[60] Fleischmann RM, Alvarez DF, Bock AE, . Randomised study of PF-06410293, an adalimumab (ADL) biosimilar, compared with reference ADL for the treatment of active rheumatoid arthritis: results from weeks 26-52, including a treatment switch from reference ADL to PF-06410293. RMD Open. 2021;7(2):e001578. doi:10.1136/rmdopen-2021-001578 33883254 PMC8061859

[61] Cohen SB, Alonso-Ruiz A, Klimiuk PA, . Similar efficacy, safety and immunogenicity of adalimumab biosimilar BI 695501 and Humira reference product in patients with moderately to severely active rheumatoid arthritis: results from the phase III randomised VOLTAIRE-RA equivalence study. Ann Rheum Dis. 2018;77(6):914-921. doi:10.1136/annrheumdis-2017-212245 29514803 PMC5965346

[62] Cohen SB, Czeloth N, Lee E, Klimiuk PA, Peter N, Jayadeva G. Long-term safety, efficacy, and immunogenicity of adalimumab biosimilar BI 695501 and adalimumab reference product in patients with moderately-to-severely active rheumatoid arthritis: results from a phase 3b extension study (VOLTAIRE-RAext). Expert Opin Biol Ther. 2019;19(10):1097-1105. doi:10.1080/14712598.2019.1645114 31387417

[63] Weinblatt ME, Baranauskaite A, Dokoupilova E, . Switching from reference adalimumab to SB5 (adalimumab biosimilar) in patients with rheumatoid arthritis: fifty-two-week phase III randomized study results. Arthritis Rheumatol. 2018;70(6):832-840. doi:10.1002/art.40444 29439289 PMC6001519

[64] Weinblatt ME, Baranauskaite A, Niebrzydowski J, . Phase III randomized study of SB5, an adalimumab biosimilar, versus reference adalimumab in patients with moderate-to-severe rheumatoid arthritis. Arthritis Rheumatol. 2018;70(1):40-48. doi:10.1002/art.40336 28950421 PMC5765475

[65] Edwards CJ, Monnet J, Ullmann M, Vlachos P, Chyrok V, Ghori V. Safety of adalimumab biosimilar MSB11022 (acetate-buffered formulation) in patients with moderately-to-severely active rheumatoid arthritis. Clin Rheumatol. 2019;38(12):3381-3390. doi:10.1007/s10067-019-04679-y 31396834

[66] Wiland P, Jeka S, Dokoupilová E, . Switching to biosimilar SDZ-ADL in patients with moderate-to-severe active rheumatoid arthritis: 48-week efficacy, safety and immunogenicity results from the phase III, randomized, double-blind ADMYRA study. BioDrugs. 2020;34(6):809-823. doi:10.1007/s40259-020-00447-6 33119861 PMC7669771

[67] Wiland P, Jeka S, Dokoupilova E, . Fri0087: efficacy, safety, and immunogenicity results of the switch from reference adalimumab (Refadl) to Sandoz biosimilar adalimumab (Gp2017, SDZ-ADL) from ADMYRA phase 3 study in patients with moderate-to-severe rheumatoid arthritis (RA). Ann Rheum Dis. 2019;78:706-707. doi:10.1136/annrheumdis-2019-eular.960

[68] Matsuno H, Kang YM, Okada M, . Comparison of the efficacy and safety of LBAL, a candidate adalimumab biosimilar, and adalimumab reference product in patients with active rheumatoid arthritis inadequately responding to methotrexate: a 52-week phase III randomised study. Clin Exp Rheumatol. 2022;40(5):1025-1033. doi:10.55563/clinexprheumatol/cyudn8 34251303

[69] Kay J, Jaworski J, Wojciechowski R, . Efficacy and safety of biosimilar CT-P17 versus reference adalimumab in subjects with rheumatoid arthritis: 24-week results from a randomized study. Arthritis Res Ther. 2021;23(1):51. doi:10.1186/s13075-020-02394-7 33546755 PMC7863328

[70] Furst DE, Jaworski J, Wojciechowski R, . Efficacy and safety of switching from reference adalimumab to CT-P17 (100 mg/mL): 52-week randomized, double-blind study in rheumatoid arthritis. Rheumatology (Oxford). 2022;61(4):1385-1395. doi:10.1093/rheumatology/keab460 34142111 PMC8996790

[71] Emery P, Vencovský J, Sylwestrzak A, . A phase III randomised, double-blind, parallel-group study comparing SB4 with etanercept reference product in patients with active rheumatoid arthritis despite methotrexate therapy. Ann Rheum Dis. 2017;76(1):51-57. doi:10.1136/annrheumdis-2015-207588 26150601 PMC5264222

[72] Emery P, Vencovský J, Sylwestrzak A, . 52-Week results of the phase 3 randomized study comparing SB4 with reference etanercept in patients with active rheumatoid arthritis. Rheumatology (Oxford). 2017;56(12):2093-2101. doi:10.1093/rheumatology/kex269 28968793 PMC5850652

[73] Emery P, Vencovský J, Sylwestrzak A, . Long-term efficacy and safety in patients with rheumatoid arthritis continuing on SB4 or switching from reference etanercept to SB4. Ann Rheum Dis. 2017;76(12):1986-1991. doi:10.1136/annrheumdis-2017-211591 28794078 PMC5705842

[74] Bae SC, Kim J, Choe JY, ; HERA Study Investigators. A phase III, multicentre, randomised, double-blind, active-controlled, parallel-group trial comparing safety and efficacy of HD203, with innovator etanercept, in combination with methotrexate, in patients with rheumatoid arthritis: the HERA study. Ann Rheum Dis. 2017;76(1):65-71. doi:10.1136/annrheumdis-2015-207613 26905864

[75] O’Dell J, Takeuchi T, Tanaka Y, . OP0226 randomized, double-blind study comparing CHS-0214 with etanercept in patients with active rheumatoid arthritis (RA) despite methotrexate (MTX) therapy. Ann Rheum Dis. 2016;75:143. doi:10.1136/annrheumdis-2016-eular.1800

[76] O’Dell J, Kivitz A, Takeuchi T, . SAT0162: switching from etanercept to CHS-0214: a one year, randomized, double-blind study in patients with rheumatoid arthritis. Ann Rheum Dis. 2017;76:831. doi:10.1136/annrheumdis-2017-eular.248028087506

[77] Matsuno H, Tomomitsu M, Hagino A, Shin S, Lee J, Song YW. Phase III, multicentre, double-blind, randomised, parallel-group study to evaluate the similarities between LBEC0101 and etanercept reference product in terms of efficacy and safety in patients with active rheumatoid arthritis inadequately responding to methotrexate. Ann Rheum Dis. 2018;77(4):488-494. doi:10.1136/annrheumdis-2017-212172 29259050 PMC5890628

[78] Park MC, Matsuno H, Kim J, . Long-term efficacy, safety and immunogenicity in patients with rheumatoid arthritis continuing on an etanercept biosimilar (LBEC0101) or switching from reference etanercept to LBEC0101: an open-label extension of a phase III multicentre, randomised, double-blind, parallel-group study. Arthritis Res Ther. 2019;21(1):122. doi:10.1186/s13075-019-1910-2 31113455 PMC6528252

[79] Matucci-Cerinic M, Allanore Y, Kavanaugh A, . Efficacy, safety and immunogenicity of GP2015, an etanercept biosimilar, compared with the reference etanercept in patients with moderate-to-severe rheumatoid arthritis: 24-week results from the comparative phase III, randomised, double-blind EQUIRA study. RMD Open. 2018;4(2):e000757. doi:10.1136/rmdopen-2018-000757 30487998 PMC6242015

[80] Jaworski J, Matucci-Cerinic M, Schulze-Koops H, . Switch from reference etanercept to SDZ ETN, an etanercept biosimilar, does not impact efficacy, safety, and immunogenicity of etanercept in patients with moderate-to-severe rheumatoid arthritis: 48-week results from the phase III, randomized, double-blind EQUIRA study. Arthritis Res Ther. 2019;21(1):130. doi:10.1186/s13075-019-1907-x 31138316 PMC6540397

[81] Yamanaka H, Kamatani N, Tanaka Y, . A comparative study to assess the efficacy, safety, and immunogenicity of YLB113 and the etanercept reference product for the treatment of patients with rheumatoid arthritis. Rheumatol Ther. 2020;7(1):149-163. doi:10.1007/s40744-019-00186-3 31833011 PMC7021908

[82] Strusberg I, Mysler E, Citera G, . Efficacy, safety, and immunogenicity of biosimilar etanercept (Enerceptan) versus its original form in combination with methotrexate in patients with rheumatoid arthritis: a randomized, multicenter, evaluator-blinded, noninferiority study. J Clin Rheumatol. 2021;27(6S):S173-S179. doi:10.1097/RHU.0000000000001616 33337815

[83] Yoo DH, Hrycaj P, Miranda P, . A randomised, double-blind, parallel-group study to demonstrate equivalence in efficacy and safety of CT-P13 compared with innovator infliximab when coadministered with methotrexate in patients with active rheumatoid arthritis: the PLANETRA study. Ann Rheum Dis. 2013;72(10):1613-1620. doi:10.1136/annrheumdis-2012-203090 23687260 PMC3786641

[84] Yoo DH, Prodanovic N, Jaworski J, . Efficacy and safety of CT-P13 (biosimilar infliximab) in patients with rheumatoid arthritis: comparison between switching from reference infliximab to CT-P13 and continuing CT-P13 in the PLANETRA extension study. Ann Rheum Dis. 2017;76(2):355-363. doi:10.1136/annrheumdis-2015-208786 27130908 PMC5284338

[85] Yoo DH, Racewicz A, Brzezicki J, . A phase III randomized study to evaluate the efficacy and safety of CT-P13 compared with reference infliximab in patients with active rheumatoid arthritis: 54-week results from the PLANETRA study. Arthritis Res Ther. 2016;18:82. doi:10.1186/s13075-016-0981-6 27038608 PMC4818886

[86] Kay J, Chopra A, Chandrashekara S, . OP0012: a phase 3, randomized, double-blind, active comparator study of the efficacy and safety of BOW015, a biosimilar infliximab, in patients with active rheumatoid arthritis on stable methotrexate doses. Ann Rheum Dis. 2014;73:64. doi:10.1136/annrheumdis-2014-eular.1595

[87] Taylor P, Wyand M, Knight A, Costantino C, Lassen C. FRI0163: efficacy of the biosimilar BOW015, compared to originator infliximab, initiated at moderate and severe disease activity thresholds in rheumatoid arthritis. Ann Rheum Dis. 2016;75:488-489. doi:10.1136/annrheumdis-2016-eular.4143

[88] Choe JY, Prodanovic N, Niebrzydowski J, . A randomised, double-blind, phase III study comparing SB2, an infliximab biosimilar, to the infliximab reference product Remicade in patients with moderate to severe rheumatoid arthritis despite methotrexate therapy. Ann Rheum Dis. 2017;76(1):58-64. doi:10.1136/annrheumdis-2015-207764 26318384 PMC5264229

[89] Smolen JS, Choe JY, Prodanovic N, . Comparing biosimilar SB2 with reference infliximab after 54 weeks of a double-blind trial: clinical, structural and safety results. Rheumatology (Oxford). 2017;56(10):1771-1779. doi:10.1093/rheumatology/kex254 28957563 PMC5850768

[90] Smolen JS, Choe JY, Prodanovic N, . Safety, immunogenicity and efficacy after switching from reference infliximab to biosimilar SB2 compared with continuing reference infliximab and SB2 in patients with rheumatoid arthritis: results of a randomised, double-blind, phase III transition study. Ann Rheum Dis. 2018;77(2):234-240. doi:10.1136/annrheumdis-2017-211741 29042358 PMC5867419

[91] Takeuchi T, Yamanaka H, Tanaka Y, . Evaluation of the pharmacokinetic equivalence and 54-week efficacy and safety of CT-P13 and innovator infliximab in Japanese patients with rheumatoid arthritis. Mod Rheumatol. 2015;25(6):817-824. doi:10.3109/14397595.2015.1022297 25736355 PMC4732515

[92] Matsuno H, Matsubara T. A randomized double-blind parallel-group phase III study to compare the efficacy and safety of NI-071 and infliximab reference product in Japanese patients with active rheumatoid arthritis refractory to methotrexate. Mod Rheumatol. 2019;29(6):919-927. doi:10.1080/14397595.2018.1533063 30289287

[93] Lila AM, Mazurov VI, Denisov LN, . A phase III study of BCD-055 compared with innovator infliximab in patients with active rheumatoid arthritis: 54-week results from the LIRA study. Rheumatol Int. 2019;39(9):1537-1546. doi:10.1007/s00296-019-04359-9 31292709

[94] Genovese MC, Sanchez-Burson J, Oh M, . Comparative clinical efficacy and safety of the proposed biosimilar ABP 710 with infliximab reference product in patients with rheumatoid arthritis. Arthritis Res Ther. 2020;22(1):60. doi:10.1186/s13075-020-2142-1 32216829 PMC7098142

[95] Peters JL, Sutton AJ, Jones DR, Abrams KR, Rushton L. Contour-enhanced meta-analysis funnel plots help distinguish publication bias from other causes of asymmetry. J Clin Epidemiol. 2008;61(10):991-996. doi:10.1016/j.jclinepi.2007.11.010 18538991

[96] Murad MH, Chu H, Lin L, Wang Z. The effect of publication bias magnitude and direction on the certainty in evidence. BMJ Evid Based Med. 2018;23(3):84-86. doi:10.1136/bmjebm-2018-110891 29650725 PMC5969367

