# Supplementary material for: Therapeutic Equivalence of Biosimilar and Reference Biologic Drugs in Rheumatoid Arthritis: A Systematic Review and Meta-analysis
Source: JAMA Netw Open. 2023 May 26;6(5):e2315872. doi: 10.1001/jamanetworkopen.2023.15872 (PMC10220520; doi:10.1001/jamanetworkopen.2023.15872)
Supplement: Supplement 1. — eAppendix 1. Changes to the Protocol with Reasons eAppendix 2. Detailed Description of the Eligibility Criteria and Study Selection eAppendix 3. Evidence Sources and Search Strategy eAppendix 4. Screening and Selection Process eAppendix 5. Data Collection Process eAppendix 6. Prespecified Coprimary Outcomes of Efficacy eAppendix 7. Prespecified Secondary Outcomes of Efficacy eAppendix 8. Prespecified Outcomes of Safety and Immunogenicity eAppendix 9. Risk of Bias Assessment eAppendix 10. Approximate Bayesian Computation Model and Other Approximations eAppendix 11. Bayesian Model Fitting, Model Diagnostics, and Estimation Methods eAppendix 12. Subgroup and Sensitive Analysis eAppendix 13. Contour-Enhanced Plots for Coprimary Outcomes eFigure 1. Possible Scenarios Within an Equivalence Testing eAppendix 14. Trial Sequential Analysis Based on Large, Randomized Trials eAppendix 15. Certainty of Evidence Assessment: GRADE Approach eFigure 2. Flow Diagram of Evidence, and Main Reasons for Exclusions eTable 1. Demographic and Clinical Characteristics of Participants at Baseline Per Study Arm eTable 2. Main and Adjuvant Therapy Schemes of Reference Biologics and Biosimilars From Included Trials eTable 3. Study Characteristics of Included Randomized Trials eTable 4. Margins of Equivalence, Noninferiority, or Superiority in Trials of Biosimilars and Reference Biologic Drugs, and Other Statistical Information for their Calculation eFigure 3. The Risk of Bias of Included Studies eTable 5. Risk of Bias Assessment Domains: Random Sequence Generation, Allocation Concealment, and Inconsistent Application of Eligibility Criteria eTable 6. Risk of Bias Assessment Domains: Blinding of Participants and Investigator, and Participant’s Behaviour Changes eTable 7. Risk of Bias Assessment Domains: Blinding of Outcome Assessors, and Outcomes Measures eTable 8. Risk of Bias Assessment Domain: Incomplete Outcome Data eTable 9. Effects of Biosimilars and Biologics on ACR20 Response: Bayesian R [file jamanetwopen-e2315872-s001.pdf]

Supplemental Online Content

Ascef BO, Almeida MO, Medeiros-Ribeiro AC, Oliveira de Andrade DC, de Oliveira Junior HA, de Soárez PC. Therapeutic equivalence of biosimilars and reference biologic drugs in rheumatoid arthritis: a systematic review and meta-analysis. *JAMA Netw Open*. 2023;6(5):e2315872. doi:10.1001/jamanetworkopen.2023.15872

eAppendix 1. Changes to the Protocol with Reasons
eAppendix 2. Detailed Description of the Eligibility Criteria and Study Selection
eAppendix 3. Evidence Sources and Search Strategy
eAppendix 4. Screening and Selection Process
eAppendix 5. Data Collection Process
eAppendix 6. Prespecified Coprimary Outcomes of Efficacy
eAppendix 7. Prespecified Secondary Outcomes of Efficacy
eAppendix 8. Prespecified Outcomes of Safety and Immunogenicity
eAppendix 9. Risk of Bias Assessment
eAppendix 10. Approximate Bayesian Computation Model and Other Approximations
eAppendix 11. Bayesian Model Fitting, Model Diagnostics, and Estimation Methods
eAppendix 12. Subgroup and Sensitive Analysis
eAppendix 13. Contour-Enhanced Plots for Coprimary Outcomes
eFigure 1. Possible Scenarios Within an Equivalence Testing
eAppendix 14. Trial Sequential Analysis Based on Large, Randomized Trials
eAppendix 15. Certainty of Evidence Assessment: GRADE Approach
eFigure 2. Flow Diagram of Evidence, and Main Reasons for Exclusions
eTable 1. Demographic and Clinical Characteristics of Participants at Baseline Per Study Arm
eTable 2. Main and Adjuvant Therapy Schemes of Reference Biologics and Biosimilars From Included Trials
eTable 3. Study Characteristics of Included Randomized Trials
eTable 4. Margins of Equivalence, Noninferiority, or Superiority in Trials of Biosimilars and Reference Biologics Drugs, and Other Statistical Information for their Calculation
eFigure 3. The Risk of Bias of Included Studies
eTable 5. Risk of Bias Assessment Domains: Random Sequence Generation, Allocation Concealment, and Inconsistent Application of Eligibility Criteria
eTable 6. Risk of Bias Assessment Domains: Blinding of Participants and Investigator, and Participant’s Behaviour Changes
eTable 7. Risk of Bias Assessment Domains: Blinding of Outcome Assessors, and Outcomes Measures
eTable 8. Risk of Bias Assessment Domain: Incomplete Outcome Data
eTable 9. Effects of Biosimilars and Biologics on ACR20 Response: Bayesian Random-Effects Meta-analysis
eTable 10. Effects of Biosimilars and Biologics on ACR20 Response: Frequentist Fixed-Effects Meta-analysis
eFigure 4. Nonprespecified and Exploratory Analyses of ACR20 Including Only Studies Reporting Modified Intention-to-Treat or Intention-to-Treat Analyses
eFigure 5. Nonprespecified and Exploratory Analyses of ACR20 Including Only Studies Reporting Per-Protocol Analyses

© 2023 de Oliveira Ascef B et al. *JAMA Network Open.*

eTable 11. Effects of Biosimilars and Biologics on HAQ-DI: Bayesian Random-Effects Meta-analysis
eTable 12. Effects of Biosimilars and Biologics on HAQ-DI: Frequentist Fixed-Effects Meta-analysis
eFigure 6. Nonprespecified and Exploratory Analyses of HAQ-DI Including Only Studies Reporting Modified Intention-to-Treat or Intention-to-Treat Analyses
eFigure 7. Nonprespecified and Exploratory Analyses of ACR20 Including Only Studies Reporting Per-Protocol Analyses
eTable 13. Effects of Biosimilars and Biologics on ACR50 and ACR70: Bayesian Random-Effects Meta-analysis
eTable 14. Effects of Biosimilars and Biologics on ACR50 and ACR70: Frequentist Fixed-Effects Meta-analysis
eTable 15. Frequency of Safety Outcomes With Uncommon Events (Meta-analysis Not Performed)
eTable 16. Effects of Biosimilars and Reference Biologics on Safety and Immunogenicity Outcomes (Bayesian Random-Effects Meta-analysis)
eTable 17. Effects of Biosimilars and Reference Biologics on Safety and Immunogenicity Outcomes (Frequentist Fixed-Effects Meta-analysis)
eFigure 8. Funnel Plot for the Effects of Biosimilars Group vs References Group on ACR50 at 6 Months of Follow-up
eFigure 9. Funnel Plot for the Effects of Biosimilars Group vs References Group on ACR70 at 6 Months of Follow-up
eFigure 10. Funnel Plot for the Effects of Biosimilars Group vs References Group on the Risk of Overall Treatment-Emergent Adverse Effects
eFigure 11. Funnel Plot for the Effects of Biosimilars Group vs References Group on the Risk of Serious Treatment-Emergent Adverse Effects
eFigure 12. Funnel Plot for the Effects of Biosimilars Group vs References Group on the Risk of Injection Site Reactions
eFigure 13. Funnel Plot for the Effects of Biosimilars Group vs References Group on the Risk of Overall Discontinuation Rates
eFigure 14. Funnel Plot for the Effects of Biosimilars Group vs References Group on the Risk of Positive Antidrug Antibodies
eFigure 15. Funnel Plot for the Effects of Biosimilars Group vs References Group on the Risk of Positive Neutralizing Antibodies
eTable 18. Certainty of Evidence Assessment of Trials Comparing Biosimilars vs Their Reference Biologic Drugs in Patients With Arthritis Rheumatoid (GRADE Evidence Profile)
eReferences.

This supplemental material has been provided by the authors to give readers additional information about their work.

© 2023 de Oliveira Ascef B et al. *JAMA Network Open.*

eMETHODS

eAppendix 1. Changes to the protocol with reasons.

Number	Protocol (original analysis/ approach)	Change	Reason
1	Frequentist model	Bayesian model	We adopted the Bayesian framework because of its flexibility in terms of statistical inference.[1] Bayesian modelling facilitates clinical interpretability by readily calculating the posterior probability of equivalence (i.e., the probability that the posterior summary estimate lies within the prespecified equivalence margins).
2	Not previously considered	Trial Sequential Analysis (TSA)	Meta-analyses may report spurious significant results (type I errors) which should have been "nonsignificant." Such spurious results may be because of systematic errors (bias) or random errors due to repeated significance testing when updating meta-analyses with new trials.[2] TSA provides several advantages such as reducing the risk of detecting a difference where no difference exists without substantially increasing the risk of overlooking a clinically and statistically relevant significance. Additionally, TSA estimates the need and size of future trials, helping to conclude if the evidence is sufficient to conclude.[2]
3	Subgroup analysis (Sample size [average of > 100 patients per group vs. < 100 patients per group])	Subgroup analysis (Sample size [average of ≥ 500 patients per group – large trials vs. < 500 patients per group- small trials])	We used this non-prespecified cut-off because large trials are less likely to be affected by small-study effects or publication bias.[3] A "large trial" compromises a randomised study that randomised ≥500 participants and a "small trial" refer to a randomised study that randomised <500 participants.[3]
4	Subgroup analysis for primary outcomes (Previous drug use; funding and publication status)	Not perform	There was no need to analyse per subgroup because all included trials were sponsored by industry.
5	Contour-enhanced funnel plots traditional	Contour-enhanced funnel plots aimed at equivalence and Funnel plots	Contour-enhanced funnel plots help to identify the areas of the funnel plot that correspond to different levels of statistical significance, and it is useful for assessing publication bias in trials testing superiority.[4] In the context of equivalence and non-inferiority, it is relevant to enhance the statistical testing areas of equivalence to help to understand how small-study effects

© 2023 de Oliveira Ascef B et al. *JAMA Network Open.*

Number	Protocol (original analysis/ approach)	Change	Reason
			act in this context. For all other outcomes, traditional funnel plots with a pseudo 95% confidence interval were presented.
6	Not previously considered	Bayesian summary effects estimates of ACR20 and HAQ-DI of large trials	We conducted a non-prespecified analysis restricted to large trials (500 or more randomised participants) to mitigate the possibility of small-study bias.
7	Not previously considered	Bayesian summary effects estimates of ACR20 and HAQ-DI by type of analyses set (only intention-to-treat or Per-protocol analyses)	We conducted a non-prespecified analysis restricted to studies reporting intention-to-treat or Per-protocol analyses to mitigate the possibility of incomplete outcome bias.

© 2023 de Oliveira Ascef B et al. *JAMA Network Open.*

eAppendix 2. A detailed description of the eligibility criteria and study selection.

Eligibility criteria

Participants: patients with rheumatoid arthritis (RA) that had been diagnosed with validated and established international criteria. No limitations were imposed based on age, baseline RA severity, sex, lines of treatment (e.g., treatment-naïve patients or second line of treatment), or any other major demographic characteristics.

Interventions and comparators: any biosimilars of adalimumab, etanercept, and infliximab. Comparators of interest were the reference biologic drugs (i.e., adalimumab, etanercept, and infliximab originals). No restrictions were imposed on dosages, treatment schedules, co-treatment, or combined therapies. We chose these three main biologics because they belong to the same drug class of TNFi with comparative safety and effectiveness in the management of RA[6] Also, these three DMARDS are the most prescribed first-line biologic therapy and have the highest numbers of approved biosimilars for RA in the market.[7, 8] No restrictions were imposed on dosages, treatment schedules, co-treatment, or combined therapies.

Type of study: eligible trials were randomized controlled trials or quasi-randomized controlled trials of equivalence, non-inferiority, and superiority. We included all trials comparing biosimilars to reference biologic drugs irrespective of the type of statistical design (superiority, equivalence, or non-inferiority). A quasi-randomized trial was defined as a prospective interventional study whose allocation sequence was not truly random (e.g., consecutive order, day of the week, date of birth, etc.). For trials with a 2-part study design, we considered results from the first period (biosimilarity) only to avoid carry-over effects.

© 2023 de Oliveira Ascef B et al. *JAMA Network Open.*

eAppendix 3. Evidence sources and search strategy.

In our protocol, a detailed description of evidence sources was provided[9]. The search strategy was initially run from database inception until 07 September 2021 using the following electronic databases: MEDLINE via PubMed, EMBASE, Cochrane Central Register of Controlled Trials (CENTRAL), and Latin American and Caribbean Health Science (LILACS). We searched for non-published or ongoing trials in the main register databases of clinical trials, the EU Clinical Trial Register (https://www.clinicaltrialsregister.eu), International Clinical Trials Registry Platform-World Health Organization (http://apps.who.int/trialsearch/) and Clinicaltrials (https://clinicaltrials.gov/).

Also, we manually screen the references of all included trials as well as previous systematic reviews. A citation search was done in Google Scholar and Epistemonikos (https://www.epistemonikos.org/) to retrieve relevant reports citing all relevant included articles. No language limitation was imposed.

Previously, we published the search strategy in our protocol[9], and we reproduced it here as follow:

Database (molecule)	Search Strategy
PubMed - etanercept	((((((((("Arthritis, Rheumatoid"[Mesh]) OR Rheumatoid Arthritis)) OR ((((("Arthritis, Juvenile"[Mesh]) OR "Rheumatoid Arthritis, Systemic Juvenile" [Supplementary Concept]) OR Juvenile Arthritis) OR Arthritis, Juvenile Chronic) OR Arthritis, Juvenile Idiopathic)))))) AND (((((((((("Biosimilar Pharmaceuticals"[Mesh]) OR Pharmaceuticals, Biosimilar) OR Follow-on Biologics) OR Biologics, Follow-on) OR Follow on Biologics) OR Subsequent Entry Biologics) OR Biologics, Subsequent Entry) OR Biosimilars)))) AND (((((((((("Etanercept"[Mesh]) OR TNFR-Fc Fusion Protein) OR Recombinant Human Dimeric TNF Receptor Type II IgG Fusion Protein) OR TNF Receptor Type II IgG Fusion Protein) OR Erelzi) OR ("GP2015" [Supplementary Concept] OR "LBEC0101" [Supplementary Concept]))) OR benepali) OR etanercept szzs)))
PubMed - infliximab	((((((((((("Arthritis, Rheumatoid"[Mesh]) OR Rheumatoid Arthritis)) OR ((((("Arthritis, Juvenile"[Mesh]) OR "Rheumatoid Arthritis, Systemic Juvenile" [Supplementary Concept]) OR Juvenile Arthritis) OR Arthritis, Juvenile Chronic) OR Arthritis, Juvenile Idiopathic))))))))) AND (((((((((((((("Biosimilar Pharmaceuticals"[Mesh]) OR Pharmaceuticals, Biosimilar) OR Follow-on Biologics) OR Biologics, Follow-on) OR Follow on Biologics) OR Subsequent Entry Biologics) OR Biologics, Subsequent Entry) OR Biosimilars))))))) AND (((((((("Infliximab"[Mesh])) OR "SB2 infliximab" [Supplementary Concept]) OR "GP1111" [Supplementary Concept])) OR "CT-P13" [Supplementary Concept])) OR (Infliximab-abda[Text Word]) OR Infliximab-dyyb[Text Word]) OR Infliximab-qbtx[Text Word]) OR Infliximab-axxq[Text Word]) OR Inflectra[Text Word]) OR Renflexis[Text Word]) OR Ixifi[Text Word]) OR Zessly[Text Word]) OR Flixabi) OR Remsima[Text Word])))
PubMed - adalimumab	((((((((((("Arthritis, Rheumatoid"[Mesh]) OR Rheumatoid Arthritis)) OR ((((("Arthritis, Juvenile"[Mesh]) OR "Rheumatoid Arthritis, Systemic Juvenile" [Supplementary Concept]) OR Juvenile Arthritis) OR Arthritis, Juvenile Chronic) OR Arthritis, Juvenile Idiopathic))))))))) AND (((((((((((((("Biosimilar Pharmaceuticals"[Mesh]) OR Pharmaceuticals, Biosimilar) OR Follow-on Biologics) OR Biologics, Follow-on) OR Follow on Biologics) OR Subsequent Entry Biologics) OR Biologics, Subsequent Entry) OR Biosimilars))))))) AND (((((((((((((((((((("Adalimumab"[Mesh]) OR D2E7 Antibody) OR Antibody, D2E7) OR Adalimumab-adbm) OR Adalimumab-atto) OR Adalimumab-adaz[Text Word]) OR Adalimumab-bwwd[Text Word]) OR Adalimumab-afzb[Text Word]) OR Amjevita[Text Word]) OR Hadlima[Text Word]) OR Cyltezo[Text Word]) OR Hyrimoz[Text Word]) OR Abrilada[Text Word]) OR Halimatoz[Text Word]) OR Hefiya[Text Word]) OR Imraldi[Text Word]) OR Hulio[Text Word]) OR Kromeya[Text Word]) OR Idacio[Text Word])) OR (((("ABP 501" [Supplementary Concept]) OR "GP2017" [Supplementary Concept]) OR "BI 695501" [Supplementary Concept]) OR "PF-06410293" [Supplementary Concept]))

© 2023 de Oliveira Ascef B et al. *JAMA Network Open.*

Database (molecule)	Search Strategy
EMBASE - etanercept	('rheumatoid arthritis'/exp OR 'rheumatoid arthritis' OR 'juvenile rheumatoid arthritis'/exp OR 'juvenile rheumatoid arthritis') AND ('biosimilar agent'/exp OR 'biosimilar agent' OR (('biosimilar' OR 'biosimilar'/exp OR biosimilar) AND pharmaceuticals) OR ('follow on' AND ('biologics' OR 'biologics'/exp OR biologics)) OR (subsequent AND entry AND ('biologics' OR 'biologics'/exp OR biologics))) AND ('etanercept'/exp OR 'etanercept' OR 'etanercept szzs'/exp OR 'etanercept szzs' OR 'erelzi' OR 'erelzi'/exp OR erelzi OR 'benepali' OR 'benepali'/exp OR benepali OR sb4 OR 'gp2015'/exp OR gp2015)
EMBASE - infliximab	('rheumatoid arthritis'/exp OR 'rheumatoid arthritis' OR 'juvenile rheumatoid arthritis'/exp OR 'juvenile rheumatoid arthritis') AND ('biosimilar agent'/exp OR 'biosimilar agent' OR (('biosimilar' OR 'biosimilar'/exp OR biosimilar) AND pharmaceuticals) OR ('follow on' AND ('biologics' OR 'biologics'/exp OR biologics)) OR (subsequent AND entry AND ('biologics' OR 'biologics'/exp OR biologics))) AND ('infliximab'/exp OR 'infliximab' OR 'inflectra' OR 'inflectra'/exp OR inflectra OR 'ixifi' OR 'ixifi'/exp OR ixifi OR 'renflexis' OR avsola OR 'zessly'/exp OR zessly OR 'remsima'/exp OR remsima OR 'renflexis'/exp OR renflexis OR 'infliximab dyyb'/exp OR 'infliximab dyyb' OR 'infliximab qbtx'/exp OR 'infliximab qbtx' OR 'infliximab axxq' OR 'infliximab abda'/exp OR 'infliximab abda' OR ctp13 OR sb2 OR 'abp710'/exp OR abp710 OR 'gp1111'/exp OR gp1111 OR 'pf06438179'/exp OR pf06438179) AND [embase]/lim
EMBASE - adalimumab	(('rheumatoid arthritis'/exp OR 'rheumatoid arthritis' OR 'juvenile rheumatoid arthritis'/exp OR 'juvenile rheumatoid arthritis') AND ('biosimilar agent'/exp OR 'biosimilar agent' OR (('biosimilar' OR 'biosimilar'/exp OR biosimilar) AND pharmaceuticals) OR ('follow on' AND ('biologics' OR 'biologics'/exp OR biologics)) OR (subsequent AND entry AND ('biologics' OR 'biologics'/exp OR biologics))) AND ('adalimumab'/exp OR 'adalimumab' OR 'amjevita' OR 'amjevita'/exp OR amjevita OR 'cyltezo' OR 'cyltezo'/exp OR cyltezo OR hadlima OR 'hyrimoz' OR 'hyrimoz'/exp OR hyrimoz OR 'adalimumab atto'/exp OR hefiya OR imraldi OR hulio OR kromeya OR idacio OR abrilada OR 'adalimumab atto' OR 'adalimumab adbm'/exp OR 'adalimumab adbm' OR 'adalimumab adaz'/exp OR 'adalimumab adaz') OR 'adalimumab afzb' OR abp501 OR gp2017 OR sb5 OR fkb327 OR msb11022 OR pf06410293) AND [embase]/lim
CENTRAL - etanercept	#1 MeSH descriptor: [Biosimilar Pharmaceuticals] explode all trees<br>#2 MeSH descriptor: [Etanercept] explode all trees<br>#3 #1 AND #2
CENTRAL - infliximab	#1 MeSH descriptor: [Biosimilar Pharmaceuticals] explode all trees<br>#2 MeSH descriptor: [Infliximab] explode all trees<br>#3 #1 AND #2
CENTRAL - adalimumab	#1 MeSH descriptor: [Biosimilar Pharmaceuticals] explode all trees<br>#2 MeSH descriptor: [Adalimumab] explode all trees<br>#3 #1 AND #2
LILACS – etarnercept	biosimilar [Palavras] and etanercept [Palavras]
LILACS - infliximab	biosimilar [Palavras] and infliximab [Palavras]
LILACS - adalimumab	biosimilar [Palavras] and adalimumab [Palavras]
EU Clinical Trial Register- etanercept	biosimilar AND etanercept AND rheumatoid arthritis
EU Clinical Trial Register- infliximab	biosimilar AND infliximab AND rheumatoid arthritis
EU Clinical Trial Register- adalimumab	biosimilar AND adalimumab AND rheumatoid arthritis
Clinical Trial NIH- etanercept	BIOSIMILAR | Etanercept | rheumatoid arthritis

© 2023 de Oliveira Ascef B et al. *JAMA Network Open.*

**Database (molecule)**	**Search Strategy**
Clinical Trial NIH- infliximab	BIOSIMILAR | Infliximab | rheumatoid arthritis
Clinical Trial NIH- adalimumab	BIOSIMILAR | Adalimumab | rheumatoid arthritis
ICTRP- etanercept	biosimilar AND etanercept AND rheumatoid arthritis
ICTRP- infliximab	biosimilar AND infliximab AND rheumatoid arthritis
ICTRP- adalimumab	biosimilar AND adalimumab AND rheumatoid arthritis

© 2023 de Oliveira Ascef B et al. *JAMA Network Open.*

eAppendix 4. Screening and selection process.

We used a customized web platform for data extraction and curation using Ragic (www.ragic.com). This database was carefully designed to simultaneously allow for study screening, selection, and data extraction for the systematic review. Two independent investigators conducted fully independently all steps. Specifically, during the screening phase, two reviewers evaluated titles and abstracts, and disagreements were solved by a consensus. After that, for each study selected, full-length articles were downloaded, and then the eligibility criteria of pre-selected trials were re-assessed by the two reviewers and a third reviewer was consulted for discordances. The main reasons to exclude were categorized and reported.

© 2023 de Oliveira Ascef B et al. *JAMA Network Open.*

eAppendix 5. Data collection process.

We collected preferentially the population per-protocol (PP) data for our analysis because this approach frequently results in narrower confidence intervals than the intention-to-treat analysis and can be more conservative for equivalence testing.[10-12] Two investigators extracted all data independently, and discrepancies sound was solved via a consensus or consultation with a third reviewer. Further details on data extraction management were given in our protocol.[9]

The following categories of information were obtained from the included trials: (i) Study details; (ii) Baseline characteristics of participants; and (iii) Study outcomes variable (continuous and binary).

(i) Study details characteristics: year of publication, sample size, trial design characteristics, trial duration (time from randomization until end of follow-up) per phase of the study, i.e., efficacy and switching phase, study registration details, statistical information regarding the calculation of margins of equivalence or non-inferiority for the study primary outcome.

(ii) Baseline characteristics of participants: demographic characteristics (age, sex), clinical characteristics (RA severity, rheumatoid factor positive, etc), and characteristics of treatments (dosage, frequency of administration, and adjuvant therapy).

(iii) Outcomes:

All outcomes were prespecified in the registered PROSPERO synopses and were categorized into three types: efficacy (encompassing outcomes related to disease activity, functional capacity, quality of life, and structural damage progression), safety, and immunogenicity.

For efficacy outcomes, we extracted data at the following time points: 1 month (± 2 weeks), 3 months (± 4 weeks), 6 months (± 4 weeks), 8 months (± 4 weeks), 12 months (± 4 weeks). For safety and immunogenicity outcomes, we collected data from the longest follow-up available.

For continuous outcomes, we extracted: the mean, standard deviations, standard error, median, interval interquartile 25% and 75%, minimum and maximum, n total participants, confidence intervals, and *P* values. These values were extracted from baseline, follow-up, and the change from baseline when it is possible.

For binary outcomes, the number of patients with the event and the number total of participants were extracted.

© 2023 de Oliveira Ascef B et al. *JAMA Network Open.*

eAppendix 6. Prespecified primary and co-primary outcomes of efficacy.

The prespecified primary outcome of efficacy was the treatment success at 6 months according to the American College of Rheumatology 20% response criteria (ACR20), which is a clinician-reported outcome, and broadly measured in RA trials.[13] We also prespecified The Health Assessment Questionnaire - Disability Index (HAQ-DI) change from baseline to 6 months as the co-primary outcome, which is a patient-reported outcome. [14] The decision to have a primary and co-primary outcome was based on the need to address different response domains to therapy. [9] While ACR20 captures the disease severity change over time (from a clinical perspective), HAQ-DI measures the self-perceived disability, directly incorporating the patient's perceived benefits from the treatment. There is compelling evidence indicating that clinical responses and patient-perceived benefits of therapy should be considered jointly when inferring the efficacy of biological therapies in patients with RA.[15-17]

The ACR20 requires at least a 20% improvement in the core set measures for a patient to reach improvement.[13] Specifically, a participant was a responder if the following 3 criteria for improvement from Baseline were met: ≥ 20% improvement in TJC; ≥ 20% improvement in SJC; and ≥ 20% improvement in at least 3 of the 5 following parameters: Patient's assessment of pain (VAS 0 a 100 mm); Patient's global assessment of disease activity (Likert scale from 0 to 10); Physician's global assessment of disease activity (Likert scale from 0 to 10); Patient's self-assessment of physical function (HAQ-DI); CRP level. ACR20 was summarised as relative risk (RR) with an RR greater than 1.0, indicating a higher response probability with biosimilar drugs than in reference biologics.

The co-primary outcome HAQ-DI assesses the functional status of patients through the evaluation of eight domains of daily life activities. The highest score reported for any component question in each domain determines the final score for that domain. By convention, the overall disability index is expressed on a 0 to 3 scale, representing an average score across the domains. A HAQ-DI of 0 indicates no functional disability, whereas a HAQ-DI of 3 denotes severe functional disability.[14] HAQ-DI was presented as a standardised mean difference (SMD, Cohen's effect size), and an SMD smaller than 0 indicates a better outcome for biosimilar drugs than in reference biologics.

© 2023 de Oliveira Ascef B et al. *JAMA Network Open.*

eAppendix 7. Prespecified secondary outcomes of efficacy.

The following prespecified secondary outcomes of efficacy were assessed at 6 months of follow-up:

Measures of clinical response:

a) Clinical outcomes (binary): the American College of Rheumatology criteria with 50% (ACR50) and 70% (ACR70) responses. These outcomes were summarised as RR and with an RR greater than 1.0, indicating a higher response probability with biosimilar drugs than in reference biologics.

© 2023 de Oliveira Ascef B et al. *JAMA Network Open.*

eAppendix 8. Prespecified outcomes of safety and immunogenicity.

The prespecified safety outcomes included: the proportion of patients with treatment-emergent adverse events (TEAEs), serious TEAEs, all-cause mortality, treatment-related mortality, and special adverse events of interest including infusion-related reactions (IRRs), injection site reactions (ISRs), hypersensitivity, malignancies, active tuberculosis, and serious infections. We also assessed the overall discontinuation rates (dropouts). Those outcomes were calculated as RR and a RR greater than 1.0 indicates that the risk of the outcome is increased by the biosimilar drugs compared to reference biologics.

For immunogenicity outcomes, we assessed the proportion of patients with positive anti-drug antibodies (ADAs), and the proportion of patients with ADAs who developed positive neutralizing antibodies (NAbs). Both outcomes were summarised as RR and a RR greater than 1.0 indicates that the risk of the outcome is increased by the biosimilar drugs compared to reference biologics.

© 2023 de Oliveira Ascef B et al. *JAMA Network Open.*

eAppendix 9. Risk of bias assessment.

Two review authors independently assessed the risk of bias in the included studies. We used the Cochrane Risk of bias tool (1.0) to evaluate the following domains: random sequence generation, allocation concealment, blinding of participants and investigators, blinding of outcome assessors, and incomplete outcome data (PP and ITT population analysis).[18] We referred to the recommendations by the US Agency for Healthcare Research and Quality, and we addressed specifically domains of equivalence or non-inferiority trials[11] including the inconsistent application of inclusion/exclusion criteria, patients selected for anticipated nonresponse or good response in one arm, patient behaviour changes, inadequate outcome measurement techniques, and incomplete outcome data (PP and ITT population analysis: only ITT population analysis may underestimate the treatment effect in equivalence/non-inferiority trials).

We rated each domain of risk of bias as being at a low, unclear, or high risk of bias. The trials were considered a high, unclear, or low risk of bias study if:

- If the trial is judged to be at high risk of bias in one or more domains, we considered it a high-risk-of-bias study.
- If the trial is judged to be at unclear risk of bias in one domain but not to be at high risk of bias for any domain, we considered it as an unclear risk of bias study.
- If the trial is judged to be a low risk of bias in all domains, we considered it a low-risk-of-bias study.

Detailed information about the criteria of the judgement of each domain is available in our published protocol and reproduced here. [9] We use *robvis* tool to create risk-of-bias plots.[19]

© 2023 de Oliveira Ascef B et al. *JAMA Network Open.*

eAppendix 10. Approximate Bayesian computation model and other approximations.

We used an Approximate Bayesian Computation (ABC) model [20] to estimate mean and standard deviations based on the median, interquartile range, and/or min-max available.

© 2023 de Oliveira Ascef B et al. *JAMA Network Open.*

eAppendix 11. Bayesian model fitting, model diagnostics, and estimation methods.

Model parameters were estimated by Markov chain Monte Carlo simulations (Gibbs sampling). Models were fitted with three chains, a burn-in period of 250,000 simulations, and 166,667 additional simulations (totalizing 500,000 iterations). We checked convergence by examining trace plots and the Gelman–Rubin statistic. Auto-correlation plots were used to check auto-correlation.

Bayesian model for binary outcomes (relative risk)

```
mode {
for (i in 1:k)	{
	rc[i] ~ dbin(pic[i],nc[i])
	rt[i] ~ dbin(pit[i],nt[i])
	mu[i] <- log(pic[i])
	deltaU[i] <- min(delta[i],-log(pic[i])/1.0000001 )
	log(pit[i]) <- mu[i] + deltaU[i]
	delta[i] ~ dnorm(delt,precision.tau)
	pic[i] ~ dunif(0,1)
	}
delt ~ dnorm(0,0.00001)
precision.tau <- 1/tau.squared
tau.squared <- tau*tau
tau ~ dunif(0,2)
RR <- exp(delt)
}
```

Bayesian model for continuous outcomes (standardized mean differences)

```
model {
	for (i in 1:k) {
	P[i] <- 1/V[i]
	es[i] ~ dnorm(delta[i], P[i])
	delta[i] ~ dnorm(g, precision.tau)
	}
g ~ dnorm(0, 0.00001)
tau~dunif(0,5)
tau.squared <-tau*tau
precision.tau <-1/(tau.squared)
}
```


© 2023 de Oliveira Ascef B et al. *JAMA Network Open.*

eAppendix 12. Subgroup and sensitive analysis.

Subgroup analyses for primary and co-primary were performed as prespecified in the protocol[9] and included:

- Type of molecule (infliximab vs. etanercept vs. adalimumab)
- Allocation concealment (low risk vs. high risk/unclear risk)
- Sample size (average of ≥ 500 patients per group vs. < 500 patients per group) (see the justification for changes in **eAppendix 1)**
- Publication status (published vs. unpublished)
- Concomitant use of synthetic disease-modifying antirheumatic drugs (yes or no)
- Trial duration (3 vs. 6 vs. 12 months)

We also performed exploratory subgroup analyses by type of reference molecule for all primary outcomes and secondary outcomes of efficacy. Also, exploratory analyses based on trial duration (1 vs. 3 vs. 6 vs. 12 months) were presented for primary and secondary outcomes of efficacy.

© 2023 de Oliveira Ascef B et al. *JAMA Network Open.*

eAppendix 13. Contour-enhanced plots for primary and co-primary outcomes.

For simplicity and ease of explanation, we assume that the treatment effect is captured in the relative risk (RR) metric, with an RR > 1 favouring biosimilar drugs compared to reference biologics (e.g., higher probability of an ACR20 response).

To build the contour-enhanced plots aimed at equivalence testing, we considered that equivalence trials frequently employ the two one-sided tests (TOST) procedure[21] and establish equivalence with a (1–2α) × 100% confidence interval. Thus, for a type-I error of 5%, most trials employ 90% confidence intervals. [21, 22]

Unlike traditional contour-enhanced plots, which present the areas of statistical significance for superiority testing, we built contour-enhanced plots that show four major regions pertinent to equivalence testing. [23]

- Superiority (not equivalent) (green area); regions in which the point estimate and 90% CI obtained in a randomised trial are entirely above the upper equivalence margin. These regions refer to areas where biosimilar drugs are considered superior and not equivalent to reference biologics.
- Inferiority (not equivalence) (red area); regions where the point estimate and 90% CI are entirely below the lower equivalence margin. These are the regions where biosimilar drugs are considered inferior and not equivalent to reference biologics.
- Equivalent (blue area); regions in which the point estimate and 90% CI are completely included within the equivalence margin.
- Inconclusive (white area); any other scenario where equivalence is uncertain.

These four regions capture eight potential scenarios (eFigure 1)**:**

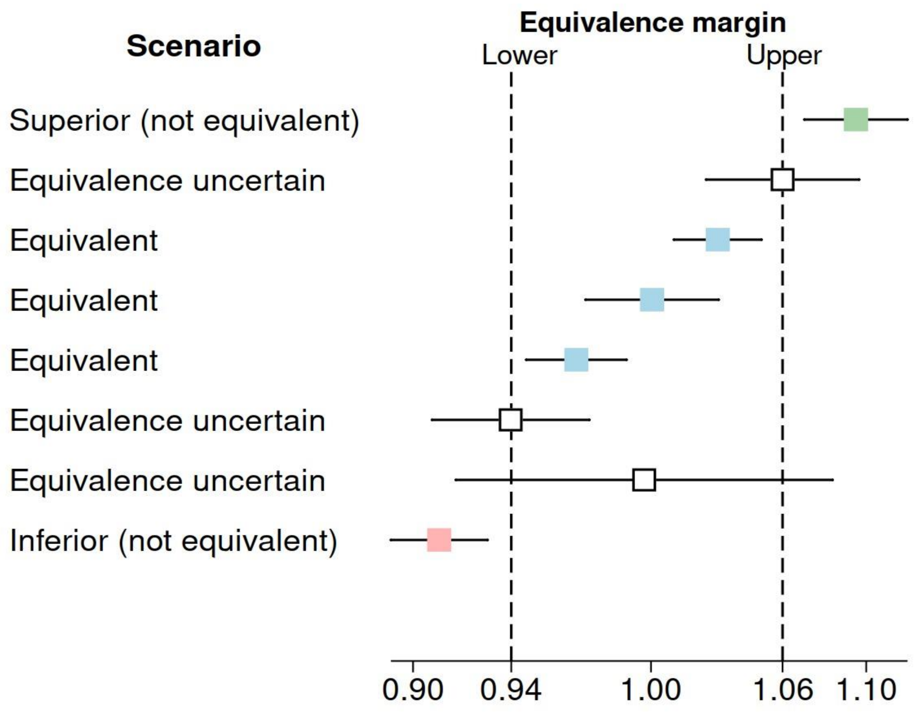

eFigure 1. Possible scenarios within an equivalence testing.
This figure shows the eight potential scenarios captured within four major regions (inferiority, superiority equivalence, and inconclusive) pertinent to equivalence testing in the contour-enhanced plot.

The plots assume that all trials used the same equivalence margin. Contour-enhanced plots were built with the same equivalence margins as the primary outcome (RR; [0.94,1.06] and co-primary outcome (SMD; [-0.22,0.22]).

© 2023 de Oliveira Ascef B et al. *JAMA Network Open.*

eAppendix 14. Trial sequential analysis based on large, randomized trials.

We conducted trial sequential analyses (non-prespecified).[24] These analyses aimed at determining whether the accrued number of participants across all large trials offered sufficient statistical power to produce definitive conclusions about the equivalence between biosimilar drugs and their reference biologics. Z-scores were calculated with the inverse-variance random-effects model, as per our protocol.[9] We used the restricted maximum-likelihood estimator of the between-trial variance.

We used the alpha spending approach (O'Brien-Fleming method)[25] to build monitoring boundaries. We also used "inner wedge" boundaries (also known as "futility boundaries"), which represent the adjusted non-superiority and non-inferiority boundaries. When the Z-score of the summary meta-analysis estimates crosses the "inner wedge" line, we have conclusive evidence that the two interventions did not differ more than the pre-specified treatment effect (i.e., they are equivalent). [26]

We calculated the required information size (RIS) considering a more stringent α level and statistical power. The RIS defines the minimum sample size needed to obtain reliable conclusions about the relative treatment effects of two interventions. For the ACR20 outcome, we calculated the RIS for a two-arm equivalence trial assuming 90% power and two-sided $\alpha = 0.005$. We considered the proportion of ACR20 responses to be 80% in the reference biologics group. We assumed that a difference in ACR20 response of ±4.8% would still allow biosimilar drugs to be equivalent (i.e., the relative risk would lie within the pre-specified [0.94,1.06] equivalence margins).

For the HAQ-DI outcome, we calculated the required information size as the sample size that provides a single trial of 90% power at a two-sided $\alpha=0.005$ to detect equivalence assuming limits of equivalence at 0.22 SD units (i.e., the difference between biosimilar drugs and reference biologics could be up to 0.22 SMD in either direction).

We accounted for between-trial variability using diversity (D2) index-adjusted sample sizes. We use a D2 of 50% in both RIS calculations.[26]

© 2023 de Oliveira Ascef B et al. *JAMA Network Open.*

eAppendix 15. Certainty of evidence assessment: GRADE approach.

We followed the recommendations of the GRADE Workgroup to assess the overall certainty of evidence.[27] Additionally, we applied recommendations for the context of systematic review and meta-analysis of equivalence and non-inferiority trials.[11] We considered specific factors of equivalence and non-inferiority trials to assess the risk of bias as previously reported, and we used clinician-oriented, prespecified margins of equivalence in a Bayesian framework. Of note, this is the first time that the certainty of the evidence is fully assessed in a systematic review of equivalence between biosimilars and reference biologics including patients with rheumatoid arthritis.[11, 28]

We assessed the five domains to determine evidence certainty. Specifically, our judgement was based on the width of the Bayesian CrI around the effect estimate, the magnitude of the effect estimate, and the overlap of the %95 credible intervals (CrI), sample size, and the number of events. Here, we described all judgement criteria applied to assess the overall certainty of evidence.

1. GRADE: factors determining the certainty of the evidence

According to the GRADE approach, the quality of evidence rating begins with the study design (trials or observational studies) and then addresses five reasons to possibly rate down the quality of evidence. [27] As all meta-analyses data in this study were provided by randomized controlled trials, we have rated down the evidence if:

a) Study limitations (Risk of bias)

We downgraded the evidence of each outcome based on the judgment of risk of bias considering all trials included in the meta-analyses. In eAppendix 10 and our published protocol [9], we have described in detail our judgment criteria for risk of bias.

b) Inconsistency

We downgraded if:

- wide variance of point estimates across studies
- minimal or no overlap of CrI.
- I^2 was higher than 50% and analysis of between-study variance indicated possibly moderate to high heterogeneity.

c) Indirectness:

We downgraded if:

- there were differences regarding the study population or when the time of the outcome assessments differed.
- subgroup analysis detected important differences in the direction of effect estimates.

d) Imprecision:

In our published systematic review protocol [9], a detailed rationale for estimating the equivalence margins for ACR20 (primary outcome) and HAQ-DI (co-primary) was given.

We downgraded if:

- when the CrI crossed both inferior and superior limits of the equivalence margins (primary and co-primary outcomes)
- the CrI of effects estimates were wide, and the number of events is less than 400.
    - ACR20: [RR 0.94, 1.04]
    - HAQ-DI: [-0.15, 0.15 units of original scale] or [SMD -0.22, 0.22]
    - Safety outcomes: the number of events was less than 400, and [RR 0.75, 1.25]
    - Immunogenicity outcomes: the number of events was less than 400, and [RR 0.75, 1.25]

e) Publication bias

We downgraded if:

Asymmetry of funnel plot and small-study biases tests statistically significant (p>0.1).

© 2023 de Oliveira Ascef B et al. *JAMA Network Open.*

eRESULTS

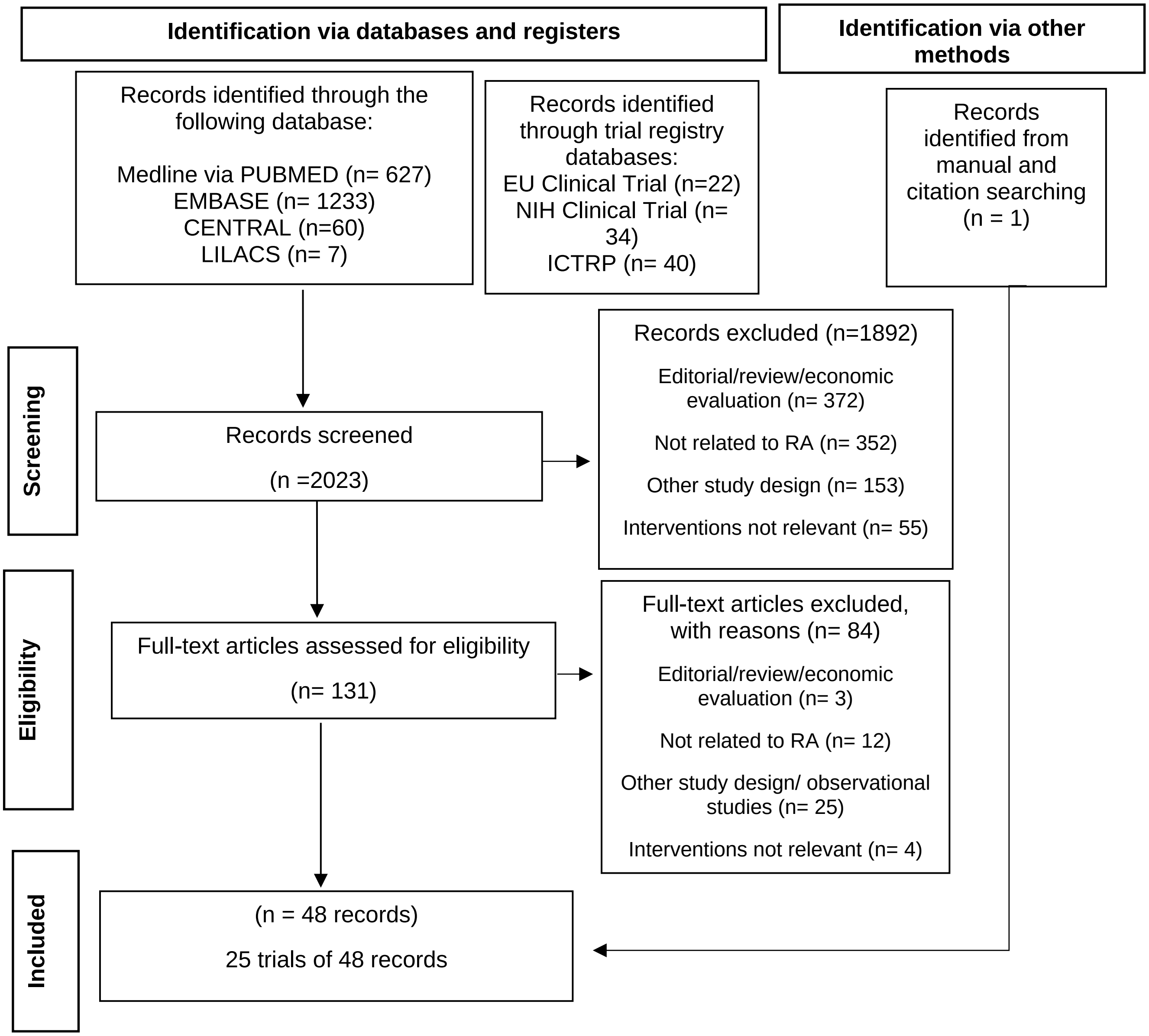

eFigure 2. Flow diagram of evidence, and main reasons for exclusions.

Source: Page et al. 2021.[5]
Abbreviations: CENTRAL, Cochrane Central Register of Controlled Trials; LILACS, Latin American and Caribbean Health Science; EU, European; NIH, National Institutes of Health; ICTRP, International Clinical Trials Registry Platform-World Health Organization; RA, rheumatoid arthritis.

© 2023 de Oliveira Ascef B et al. *JAMA Network Open.*

eTable 1. Demographic and clinical characteristics of participants at baseline per study arm.

Author. year	Arm	No. randomized patients	No. analyzed patients	Age, mean (years)	No. (%) Female	BMI, mean (kg/m^2)	RA duration, mean (years)	No. (%) Anti-CPP positive	No. (%) RF positive	CRP, mean (mg/dL)	MTX dose, mean (mg/weeks)
Jani et al, 2015 [29]	ZRC-3197	60	60	45.0	51 (85)	23.2	3.3	57 (95)	57 (95)	NA	NA
	ADA	60	60	45.0	48 (80)	23.2	4.0	55 (92)	60 (100)	NA	NA
Alten et al, 2017 [30-36]	FKB327	366	366	53.0	281 (77)	NA	8.6	--	277 (76)	2.5	15.8
	ADA	362	362	53.6	284 (78)	NA	8.3	--	277 (77)	2.7	15.8
Cohen et al, 2017 [37, 38]	ABP-501	264	264	55.4	214 (81)	NA	9.4	212 (80)	243 (92)	1.4	16.9
	ADA	262	262	56.3	212 (81)	NA	9.4	230 (88)	240 (92)	1.5	16.6
Jamshidi et al, 2017 [39]	CinnoRA	68	68	48.3	58 (85)	NA	NA	NA	NA	2.1	NA
	ADA	68	68	47.6	60 (88)	NA	NA	NA	NA	1.9	NA
Fleishmann et al, 2018 [40-42]	PF-06410293	297	297	51.5	241 (81)	27.5	6.8	NA	NA	2.1	15.2
	ADA	300	300	53.5	229 (76)	28.1	6.8	NA	NA	2.3	15.2
Cohen et al, 2018 [43, 44]	BI-695501	324	324	53.7	267 (82)	27.0	7.3	218 (67)	281 (87)	--	16.3
	ADA	321	321	53.6	269 (84)	27.9	7.0	237 (74)	281 (88)	--	16.8
Weinblatt et al, 2018 [45, 46]	SB5	271	271	49.8	217 (80)	26.2	5.4	NA	203 (75)	1.1	15.1
	ADA	273	273	52.5	224 (82)	27.0	5.5	NA	185 (68)	1.3	15.4
Edwards et al, 2019 [47]	MSB11022	143	143	53.9	108 (76)	27.3	NA	NA	NA	NA	NA
	ADA	145	145	54.0	119 (82)	27.0	NA	NA	NA	NA	NA
Willand et al, 2019 [48, 49]	GP2017	177	177	52.8	153 (86)	28.6	8.1	112 (63)	140 (79)		17.1
	ADA	176	176	53.8	142 (81)	28.0	7.4	102 (58)	135 (77)		17.6
Matsuno et al, 2021 [50]	LBAL	191	191	NA	NA	NA	NA	NA	NA	NA	NA
	ADA	190	190	NA	NA	NA	NA	NA	NA	NA	NA
Kay et al, 2021 [51, 52]	CT-P17	324	324	53.5	249 (77)	NA	6.8	NA	NA	NA	NA
	ADA	324	324	54.0	265 (82)	NA	6.6	NA	NA	NA	NA
Emery et al, 2015 [53-55]	SB4	299	299	52.1	249 (83)	26.8	6.0	NA	237 (79)	1.5	15.6
	ETN	297	297	51.6	253 (85)	26.3	6.2	NA	231 (78)	1.3	15.5
Bae et al, 2016 [56]	HD203	147	115	51.0	101 (88)	22.5	7.2	100 (87)	94 (82)	2.1	NA
	ETN	147	118	51.3	101 (86)	22.8	8.0	103 (87)	108 (92)	1.6	NA

© 2023 de Oliveira Ascef B et al. *JAMA Network Open.*

Author. year	Arm	No. randomized patients	No. analyzed patients	Age, mean (years)	No. (%) Female	BMI, mean (kg/m²)	RA duration, mean (years)	No. (%) Anti-CPP positive	No. (%) RF positive	CRP, mean (mg/dL)	MTX dose, mean (mg/weeks)
Odell et al, 2016 [57, 58]	CHS-0214	324	324	NA	260 (80)	NA	NA	NA	NA	NA	NA
	ETN	323	320	NA	254 (79)	NA	NA	NA	NA	NA	NA
Matsuno et al, 2017 [59, 60]	LBEC0101	187	185	52.8	150 (81)	NA	7.6	NA	144 (78)	1.6	11.3
	ETN	187	187	55.5	166 (89)	NA	7.8	NA	144 (77)	1.7	11.1
Matucci-Cerinic et al, 2018 [61, 62]	GP2015	186	186	55.2	158 (85)	NA	8.8	144 (77)	137 (74)	1.2	16.0
	ETN	190	190	53.1	150 (79)	NA	8.2	140 (74)	140 (74)	1.1	17.1
Yamanaka et al, 2020 [63]	YLB113	266	264	52.0	201 (76)	24.9	NA	198 (75)	189 (72)	1.3	11.4
	ETN	262	260	52.6	208 (80)	25.0	NA	187 (72)	179 (69)	1.0	11.9
Strusberg et al, 2021 [64]	Enerceptan	99	99	50.2	85 (86)	28.4	10.6	95 (96)	NA	NA	18.1
	ETN	51	50	46.3	42 (84)	27.9	10.5	48 (96)	NA	NA	17.5
Yoo et al, 2013 [65-67]	CT-P13	302	302	50.0	245 (81)	26.3	NA	205 (68)	NA	1.9	15.6
	IFX	304	304	50.0	256 (84)	25.4	NA	213 (70)	NA	1.9	15.6
Kay et al, 2014 [68, 69]	BOW015	127	127	NA	NA	NA	NA	NA	NA	NA	NA
	IFX	62	62	NA	NA	NA	NA	NA	NA	NA	NA
Choe et al, 2015 [70-72]	SB2	291	291	51.6	232 (80)	26.6	6.3	NA	215 (74)	1.2	14.7
	IFX	293	293	52.6	236 (81)	26.5	6.6	NA	208 (71)	1.4	14.7
Takeuchi, et al 2015 [73]	CT-P13	51	50	54.5	40 (80)	NA	7.1	42 (84)	43 (86)	2.1	9.7
	IFX	53	51	53.8	41 (80)	NA	8.0	48 (94)	45 (88)	2.3	9.5
Matsuno et al, 2018 [74]	NI071	126	126	54.0	108 (86)	NA	NA	43 (34)	51 (40)	1.9	9.4
	IFX	116	116	53.7	96 (83)	NA	NA	36 (31)	44 (38)	1.8	9.9
Lila et al, 2019 [75]	BCD-055	284	280	53.0	227 (81)	NA	3.8	213 (76)	NA	2.7	15.0
	IFX	142	138	53.0	110 (80)	NA	4.4	113 (82)	NA	2.3	15.0
Genovese et al, 2020 [76]	ABP710	279	279	55.0	214 (77)	NA	8.7	253 (91)	244 (87)	1.4	17.5
	IFX	279	279	54.8	223 (80)	NA	8.3	253 (91)	251 (90)	1.5	17.2
Total	**Min**	51	50	45.0	76%	22.5	3.3	31%	38%	1.0.	9.4
	**Max**	366	366	56.3	89%	28.6	10.6	96%	88%	2.7	18.1

Abbreviations: RA, rheumatoid arthritis; ADA, adalimumab; ETN, etanercept; IFX, Infliximab; BMI, body mass index; CCP, cyclic citrullinated peptide; RF, Rheumatoid factor; CRP, C reactive protein; Min, minimum values; Max, maximum values; NA, not available.

© 2023 de Oliveira Ascef B et al. *JAMA Network Open.*

eTable 2. Main and adjuvant therapy schemes of reference biologics and biosimilars from included trials.

Author, year	Arms	Drug ADM form	Dose (mg)	Therapy frequency (weeks)	Adjuvant therapy
Jani et al, 2015 [29]	ZRC-3197 and ADA	Sc	40	Every other week for 12 weeks	NA
Alten et al, 2017 [30-36]	FKB327 and ADA	Sc	40	Every 2 weeks	Concomitant MTX and folate treatment
Cohen et al, 2017 [37, 38]	ABP-501 and ADA	Sc	40	On day 1 and then every 2 weeks until week 22	Patients were required to receive a stable dose of MTX for the study duration, as prescribed by the treating physician
Jamshidi et al, 2017 [39]	CinnoRA and ADA	Sc	40	Every other week for over 24 weeks	MTX (15 mg/week), folic acid (1 mg/day), and prednisolone (7.5 mg/day)
Fleishmann et al, 2018 [40-42]	PF-06410293 and ADA	Sc	40	Every other week	Dose of oral or intramuscular MTX (10–25 mg/week) and oral folic/folinic acid;
Cohen et al, 2018 [43, 44]	BI-695501 and ADA	Sc	40	Once every 2 weeks for 24 weeks	MTX therapy at a dose of 15 to 25 mg per week and oral folic acid (at least 5 mg/week or as per local practice) or folinic acid (at least 1 mg/week or as per local practice)
Weinblatt et al, 2018 [45, 46]	SB5 and ADA	Sc	40	Every other week	NA
Edwards et al, 2019 [47]	MSB11022 and ADA	Sc	40	Every other week starting at baseline, up to, and including week 48	NA
Willand et al, 2019 [48, 49]	GP2017 and ADA	Sc	40	Every other week from Week 0 to 22	NA
Matsuno et al, 2021 [50]	LBAL and ADA	Sc	40	Every two weeks for 24 weeks	NA
Kay et al, 2021 [51, 52]	CT-P17 and ADA	Sc	40	Every 2 weeks until week 24	Subjects also received treatment with MTX (12.5–25 mg/week or 10 mg/week if intolerant to a higher dose, oral or parenteral [intramuscular or Sc] dose), and folic acid (≥ 5 mg/week, oral)
Emery et al, 2015 [53-55]	SB4 and ETN	Sc	50	Once weekly for up to 52 weeks	MTX (10–25 mg/week) and folic acid (5–10 mg/week)
Bae et al, 2016 [56]	HD203 and ETN	Sc	25	Twice weekly for 48 weeks	MTX (7.5−25 mg/week orally, intramuscularly, or SC)
Odell et al, 2016 [57, 58]	CHS_0214 and ETN	Sc	50	Every week for 24 weeks	Stable dose of MTX

© 2023 de Oliveira Ascef B et al. *JAMA Network Open.*

Author, year	Arms	Drug ADM form	Dose (mg)	Therapy frequency (weeks)	Adjuvant therapy
Matsuno et al, 2017 [59, 60]	LBEC0101 and ETN	Sc	50	Once a week for 52 weeks	MTX was co-administered to all patients on a stable dose (7.5–15mg/week and 6–16mg/week)
Matucci-Cerinic et al, 2018 [61, 62]	GP2015 and ETN	Sc	50	Once weekly, for 24 weeks	MTX at a stable dose (10–25 mg/week), and folic acid (≥ 5 mg/week) until the end of the study
Yamanaka et al, 2020 [63]	YLB113 and ETN	Sc	50	Once weekly for 24 weeks	Stable MTX treatment within the range of 6 mg to 25 mg per week
Strusberg et al, 2021 [64]	Eterneceptan and ETN	Sc	50	Weekly for 32 weeks	NA
Yoo et al, 2013 [65-67]	CT-P13 and IFX	IV	3	2h of infusion at weeks 0, 2, and 6 and then 8 weeks up to week 30	Patients were premedicated with antihistamine (chlorpheniramine 2–4 mg or dose of equivalent antihistamine) 30–60 min before the start of infusion at the investigator's discretion. Weekly MTX (12.5–25 mg/week, oral or parenteral dose) and folic acid (≥5 mg/week, oral dose)
Kay et al, 2014 [68, 69]	BOW015 and IFX	IV	3	Weeks 0, 2, 6, and 14	NA
Choe et al, 2015 [70-72]	SB2 and IFX	IV	3	Week 0, week 2, week 6, week 14, week 22, week 30, week 38, and week 46. Dose increases could occur from week 30 by 1.5 mg/kg per visit, up to a total of 7.5 mg/kg	MTX was given as an oral or parenteral weekly dose of 10–25 mg/week with folic acid of 5–10 mg/week
Takeuchi et al, 2015 [73]	CT-P13 and IFX	IV	3	Weeks 0, 2, and 6, and each 8 weeks afterward up to week 54	MTX (stable dose of 6 – 16 mg/week administered 4 weeks before study enrollment should be maintained; oral dose) and folic acid (5 mg/week; oral dose)
Matsuno et al, 2018 [74]	NI071 and IFX	IV	3	Day 1 (first dose) and at Weeks 2 and 6 at a fixed dose of 3 mg/kg; If the efficacy was insufficient at Week 14 (based on the investigator's discretion), a stepwise dose increases and/or shortened dose interval was allowed thereafter up to 10	NA

© 2023 de Oliveira Ascef B et al. *JAMA Network Open.*

Author, year	Arms	Drug ADM form	Dose (mg)	Therapy frequency (weeks)	Adjuvant therapy
				mg/kg at an 8-week interval or up to 6 mg/kg at a shortened 4-week interval	
Lila et al, 2019 [75]	BCD-055 and IFX	IV	3	At weeks 0, 2, 6, and then every 8 weeks until week 54 inclusively	Stable doses of MTX (10–25 mg/week) and folic acid (5 mg/week)
Genovese et al, 2020 [76]	ABP710 and IFX	IV	3	On day 1 (week 0), at weeks 2 and 6, and every 8 weeks thereafter until week 22.	NA

Abbreviations: ADA, adalimumab; ETN, etanercept; IFX, Infliximab; ADM, administration; Sc, subcutaneous; IV, intravenous; MTX, methotrexate; NA, not available.

© 2023 de Oliveira Ascef B et al. *JAMA Network Open.*

eTable 3. Study characteristics of included randomized trials.

Author, year	Bios	Ref	Trial register numbers (Sponsor name)	Financial study support from commercial bodies	Settings	No of investigator centres
Jani et al, 2015 [29]	ZRC-3197	ADA	NA	Cadila Healthcare Limited, the Zydus Group Company	India	11
Alten et al, 2017 [30-36]	FKB327	ADA	NCT02260791 and NCT02405780<br><br>(ARABESC)	Yoshindo Lupin Biologics Ltd.	United States, Bulgaria, Canada, Chile, Czechia, Germany, Peru, Poland, Romania, Russian Federation, Spain, and Ukraine	105
Cohen et al, 2017 [37, 38]	ABP-501	ADA	NCT01970475 and NCT02114931	CELLTRION, Inc	Poland, Czech Republic, Hungary, Bulgaria, Romania, Russian Federation, Germany, Spain, United Kingdom, United States, Canada, and Mexico	100
Jamshidi et al, 2017 [39]	CinnoRA	ADA	IRCT2015030321315N1	LG Chem (formerly LG Life Sciences), Mochida Pharmaceutical and Korea Health Industry Development Institute	Iran	10
Fleishmann et al, 2018 [40-42]	PF-06410293	ADA	NCT02167139	Samsung Bioepis	United States, Australia, Brazil, Bulgaria, Colombia, Czechia, Estonia, Georgia, Germany, Hungary, Japan, Republic of Korea, Lithuania, Mexico, New Zealand, Peru, Poland, Russian Federation, Serbia, South Africa, Spain, Taiwan, Ukraine, and the United Kingdom	173
Cohen et al, 2018 [43, 44]	BI-695501	ADA	NCT02137226 and NCT02640612<br><br>(VOLTAIRE-RA)	Fujifilm Kyowa Kirin Biologics Co.	United States, Bulgaria, Chile, Estonia, Germany, Hungary, Republic of Korea, Malaysia, New Zealand, Poland, Russian Federation, Serbia, Spain, Thailand, and Ukraine	115
Weinblatt et al, 2018 [45, 46]	SB5	ADA	NCT02480153 and NCT02480153	Celltrion, Inc. / Nippon Kayaku Co., Ltd.	Bosnia and Herzegovina, Bulgaria, Czech Republic,	51

© 2023 de Oliveira Ascef B et al. *JAMA Network Open.*

Author, year	Bios	Ref	Trial register numbers (Sponsor name)	Financial study support from commercial bodies	Settings	No of investigator centres
			(REFLECTIONS)		Lithuania, Poland, Republic of Korea, and Ukraine	
Edwards et al, 2019 [47]	MSB1102 2	ADA	NCT03052322<br><br>(AURIEL-RA study)	Hexal AG, a Sandoz company	United Kingdom, Bulgaria, Czech Republic, Germany, Hungary, and Poland	47
Willand et al, 2019 [48, 49]	GP2017	ADA	NCT02744755	Sandoz	United States, Czechia, Germany, Hungary, Italy, Malaysia, Mexico, Poland, Romania, Russian Federation, Serbia, Spain, and the United Kingdom	83
Matsuno et al, 2021 [50]	LBAL	ADA	NCT02746380	LG Life Sciences<br><br>Mochida Pharmaceutical Company, Ltd.	Japan and the Republic of Korea	2
Kay et al, 2021 [51, 52]	CT-P17	ADA	NCT03789292	Celltrion, Inc. (Incheon, Republic of Korea)	Bulgaria, Hungary, Lithuania, Peru, Poland, and Ukraine	52
Emery et al, 2015 [53-55]	SB4	ETN	NCT01895309	Samsung Bioepis	Ukraine, United Kingdom, Bulgaria, Colombia, Czech Republic, Hungary, Republic of Korea, Lithuania, Mexico, and Poland	73
Bae et al, 2016 [56]	HD203	ETN	NCT01270997<br><br>(The HERA study)	Hanwha Chemical Biologics Co	Republic of Korea	37
Odell et al, 2016 [57, 58]	CHS-0214	ETN	NCT02115750	Boehringer Ingelheim	South Africa, Spain, United Kingdom, United States, Belarus, France, Germany, Hungary, Israel, Italy, Japan, Poland, and Russian Federation	NA
Matsuno et al, 2017 [59, 60]	LBEC010 1	ETN	NCT02357069 and NCT02715908	CinnaGen Co	Korea and Japan	78
Matucci-Cerinic et al, 2018 [61, 62]	GP2015	ETN	NCT02638259 and EUDRA CT 2012-002009-23<br><br>(EQUIRA Study)	Amgen	United States, Bulgaria, Czechia, Estonia, Germany, Hungary, Italy, Latvia, Lithuania, Mexico, Poland, Russian Federation, Serbia, Slovakia, Spain, and United Kingdom	113

© 2023 de Oliveira Ascef B et al. *JAMA Network Open.*

Author, year	Bios	Ref	Trial register numbers (Sponsor name)	Financial study support from commercial bodies	Settings	No of investigator centres
Yamanaka et al, 2020 [63]	YLB113	ETN	2015-002809-12	Nichi-Iko Pharmaceutical Co., Ltd.	Romania, Spain, Bulgaria, Czech Republic, Hungary, Latvia, Ukraine, Japan, and India	101
Strusberg et al, 2021 [64]	Enercepta n	ETN	NCT03332719 and NCT03403140 (GEMENE and GEMENE002)	Gema Biotech SAU.	Argentina	9
Yoo et al, 2013 [65-67]	CT-P13	IFX	NCT01217086 and NCT01571219 (PLANETRA)	Fresenius Kabi SwissBioSim GmbH (Merck KGaA)	Poland, Portugal, Romania, Slovakia, Spain, United Kingdom, Austria, Bulgaria, Italy, Latvia, Lithuania, Philippines, Bosnia and Herzegovina, Jordan, Ukraine, Chile, Colombia, Mexico, and Peru	100
Kay et al, 2014 [68, 69]	BOW015	IFX	NCT02683564 (The UNIFORM Study)	JSC BIOCAD	NA	NA
Choe et al, 2015 [70-72]	SB2	IFX	NCT01936181	EPIRUS Biopharmaceuticals Inc	Ukraine, United Kingdom, Bosnia and Herzegovina, Bulgaria, Czech Republic, Republic of Korea, Latvia, Lithuania, Philippines, Poland, and Romania	73
Takeuchi et al, 2015 [73]	CT-P13	IFX	JapicCTI-111620	Pfizer Inc.	Japan	20
Matsuno et al, 2018 [74]	NI071	IFX	NCT01927263	Coherus Biosciences, Inc. / Daiichi Sankyo Co., Ltd.	Japan	66
Lila et al, 2019 [75]	BCD-055	IFX	NCT02762838 (LIRA Study)	Amgen Inc.	Russia, Belarus, and India	39
Genovese et al, 2020 [76]	ABP710	IFX	NCT02937701	Samsung Bioepis Co.	United States, Australia, Bulgaria, Canada, Czechia, Germany, Hungary, Poland, and Spain	75

Abbreviations: ADA, adalimumab; ETN, etanercept; IFX, Infliximab; NA, not available.

© 2023 de Oliveira Ascef B et al. *JAMA Network Open.*

eTable 4. Margins of equivalence, non-inferiority, or superiority in trials of biosimilars and reference biologics drugs, and other statistical information for their calculation.

Author, year	Bios	Ref	Primary outcome	Study power	Unit of Measure	Upper margin	Lower margin	Reason for margins choice	CI (%)	Analysis set	Imputation techniques
Jani et al, 2015 [29]	ZRC-3197	ADA	ACR20	80%	Proportion of patients	28.5	28.5	Did not attempt to justification	95	Both mITT and PP	NA
Alten et al, 2017 [30-36]	FKB327	ADA	ACR20	80%	Proportion of patients	15	12	A general comment that margins were decided according to regulatory guidance	90	mITT only	NA
Cohen et al, 2017 [37, 38]	ABP-501	ADA	ACR20	90%	Relative risk	1.36	0.74	According to regulatory guidance and previous trials of the control arm	90	mITT only	Single imputation - LOCF
Jamshidi et al, 2017 [39]	CinnoRA	ADA	EULAR response based on DAS28-ESR	90%	Proportion of patients	NA	0.18	Clinical basis. No evidence for consultation with an external expert group. and no reference to previous trials of the control arm	97.5	Both ITT and PP	NA
Fleishmann et al, 2018 [40-42]	PF-06410293	ADA	ACR20	80%	Proportion of patients	15	15	According to regulatory guidance and previous trials of the control arm	95	Both mITT and PP	Non-responder imputation
Cohen et al, 2018 [43, 44]	BI-695501	ADA	ACR20	90%	Proportion of patients	15	12	A general comment that margins were decided according to regulatory guidance	90	Both mITT and PP	Multi methods for single imputation
Weinblatt et al, 2018 [45, 46]	SB5	ADA	ACR20	85%	Proportion of patients	14	14	According to regulatory guidance and previous trials of the control arm	95	Both mITT and PP	Non-responder imputation
Edwards et al, 2019 [47]	MSB11022	ADA	Hypersensitivity	NA	NA	NA	NA	Did not attempt to justification	95	Safety analysis set	NA

© 2023 de Oliveira Ascef B et al. *JAMA Network Open.*

Author, year	Bios	Ref	Primary outcome	Study power	Unit of Measure	Upper margin	Lower margin	Reason for margins choice	CI (%)	Analysis set	Imputation techniques
Willand et al, 2019 [48, 49]	GP2017	ADA	DAS28-CRP	90%	Mean changes difference	0.6	0.6	Did not attempt to justification	95	Both mITT and PP	NA
Matsuno et al, 2021 [50]	LBAL	ADA	DAS28-ESR	NA	Mean changes difference	0.6	0.6	NA	95	NA	NA
Kay et al, 2021 [51, 52]	CT-P17	ADA	ACR20	80%	Proportion of patients	15	15	A general comment that margins were decided according to regulatory guidance	95	Both ITT and PP	NA
Emery et al, 2015 [53-55]	SB4	ETN	ACR20	80%	Proportion of patients	15	15	According to regulatory guidance and previous trials of the control arm	95	Both mITT and PP	Non-responder imputation
Bae et al, 2016 [56]	HD203	ETN	ACR20	80%	Proportion of patients	20	20	Preservation of treatment effect based on estimates of control arm effect from previous trials;	95	Both mITT and PP	Single imputation – LOCF
Odell et al, 2016 [57, 58]	CHS-0214	ETN	ACR20	NA	Proportion of patients	15	15	Did not attempt to justification	95	PP only	NA
Matsuno et al, 2017 [59, 60]	LBEC010 1	ETN	DAS28-ESR	90%	Mean changes difference	0.6	0.6	According to regulatory guidance and previous trials of the control arm	95	Both mITT and PP	Single imputation - LOCF
Matucci-Cerinic et al, 2018 [61, 62]	GP2015	ETN	DAS28-CRP	90%	Mean changes difference	0.6	0.6	Did not attempt to justification	95	Both mITT and PP	Mixed models
Yamanaka et al, 2020 [63]	YLB113	ETN	ACR20	NA	Proportion of patients	15	15	According to regulatory guidance and previous trials of the control arm	95	Both mITT and PP	Multi methods for imputation
Strusberg et al, 2021 [64]	Enercepta n	ETN	ACR20	80%	Proportion of patients	NA	12	Preservation of treatment effect based on estimates	95	Both ITT and PP	Single imputation – LOCF

© 2023 de Oliveira Ascef B et al. *JAMA Network Open.*

Author, year	Bios	Ref	Primary outcome	Study power	Unit of Measure	Upper margin	Lower margin	Reason for margins choice	CI (%)	Analysis set	Imputation techniques
								of control arm effect from previous trials;			
Yoo et al, 2013 [65-67]	CT-P13	IFX	ACR20	80%	Proportion of patients	15	15	According to regulatory guidance and previous trials of the control arm	95	Both ITT and PP	NA
Kay et al, 2014 [68, 69]	BOW015	IFX	ACR20	NA	Proportion of patients	23	23	Did not attempt to justification	95	Both ITT and PP	NA
Choe et al, 2015 [70-72]	SB2	IFX	ACR20	80%	Proportion of patients	15	15	According to regulatory guidance and previous trials of the control arm	95	Both mITT and PP	Non-responder imputation
Takeuchi et al, 2015 [73]	CT-P13	IFX	PK equivalence	80%	Mean Area Under the Curve	125	80	Did not attempt to justification	90	mITT only	Non-responder imputation
Matsuno et al, 2018 [74]	NI071	IFX	DAS28-ESR	NA	Mean changes difference	0.6	0.6	The same margin as was used in other similar trials	95	mITT only	Single imputation - LOCF
Lila et al, 2019 [75]	BCD-055	IFX	ACR20	80%	Proportion of patients	15	15	Did not attempt to justification	95	Both mITT and PP	NA
Genovese et al, 2020 [76]	ABP710	IFX	ACR20	90%	Proportion of patients	15	15	Did not attempt to justification	90	Both ITT and PP	Non-responder imputation

Abbreviations: ADA, adalimumab; ETN, etanercept; IFX, infliximab; CI, confidence interval; ACR, the American College of Rheumatology; DAS28-ESR, Disease Activity Score in 28 joints based on the erythrocyte sedimentation rate; DAS28-CRP, Disease Activity Score in 28 joints, four components based on C-reactive protein; PK, pharmacokinetics; PP, per protocol analysis ; mITT: ,modified Intention-to-treat analysis; ITT, intention-to-treat analysis; LOCF, Last Observation Carried Forward; NA, not available.

© 2023 de Oliveira Ascef B et al. *JAMA Network Open.*

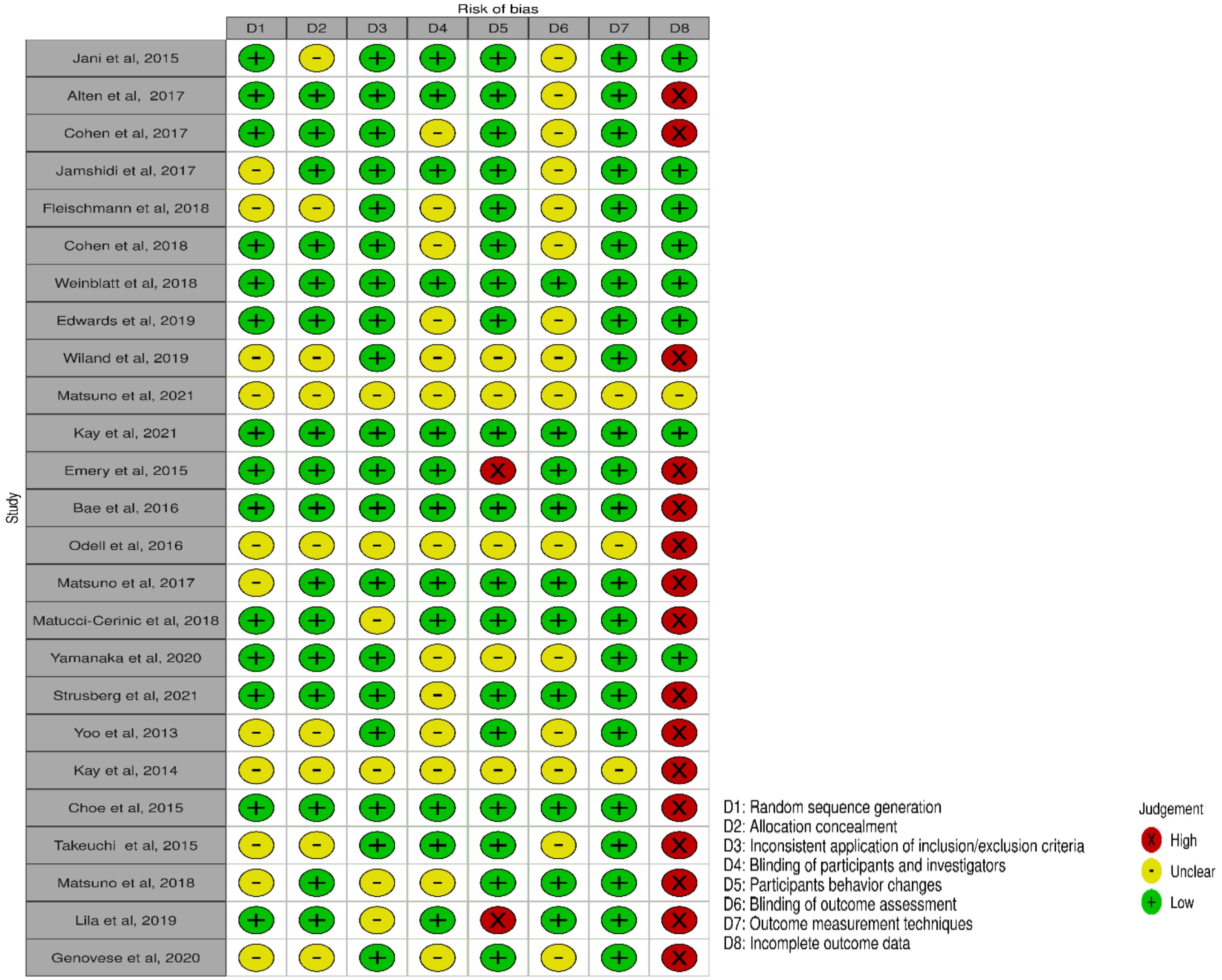

eFigure 3. The risk of bias of included studies.

Traffic light plot based on the judgement of risk of bias in 25 trials comparing biosimilars versus reference biologic drugs. Each domain was judged by two independent reviewers as low, unclear, or high risk of bias.

© 2023 de Oliveira Ascef B et al. *JAMA Network Open.*

eTable 5. Risk of Bias Assessment Domains: random sequence generation, allocation concealment, and inconsistent application of eligibility criteria.

Author, year	Random sequence generation				Allocation concealment		Inconsistent application of eligibility criteria		
	**Method**	**Properly conducted**	**Tools**	**Overall judgement domain 1**	**Method**	**Overall judgement domain 2**	**Clearly stated and implemented**	**Participants selected for no/positive response**	**Overall judgement domain 3**
Jani et al, 2015 [29]	Permuted block randomization	Yes	Using a computer random number generator	Low risk	Unclear	Unclear risk	Yes	No	Low risk
Alten et al, 2017 [30-36]	Randomization with stratification	Yes	Using a computer random number generator	Low risk	Central allocation*	Low risk	Yes	No	Low risk
Cohen et al, 2017 [37, 38]	Randomization with stratification	Yes	Using a computer random number generator	Low risk	Central allocation*	Low risk	Yes	No	Low risk
Jamshidi et al, 2017 [39]	Permuted block randomization	Unclear	Unclear	Unclear risk	Central allocation*	Low risk	Yes	No	Low risk
Fleishmann et al, 2018 [40-42]	Randomization with stratification	Unclear	Unclear	Unclear risk	Unclear	Unclear risk	Yes	No	Low risk
Cohen et al, 2018 [43, 44]	Permuted block randomization with stratification	Yes	Using a computer random number generator	Low risk	Central allocation*	Low risk	Yes	No	Low risk
Weinblatt et al, 2018 [45, 46]	Randomization with stratification	Yes	Using a computer random	Low risk	Central allocation*	Low risk	Yes	No	Low risk

© 2023 de Oliveira Ascef B et al. *JAMA Network Open.*

Author, year	Random sequence generation				Allocation concealment		Inconsistent application of eligibility criteria		
	Method	Properly conducted	Tools	Overall judgement domain 1	Method	Overall judgement domain 2	Clearly stated and implemented	Participants selected for no/positive response	Overall judgement domain 3
			number generator						
Edwards et al, 2019 [47]	Permuted block randomization with stratification	Yes	Using a computer random number generator	Low risk	Central allocation*	Low risk	Yes	No	Low risk
Willand et al, 2019 [48, 49]	Simple randomization	Unclear	Unclear	Unclear risk	Unclear	Unclear risk	Yes	No	Low risk
Matsuno et al, 2021 [50]	Unclear	Unclear	Unclear	Unclear risk	Unclear	Unclear risk	Unclear	Unclear	Unclear risk
Kay et al, 2021 [51, 52]	Permuted block randomization with stratification	Yes	Using a computer random number generator	Low risk	Central allocation*	Low risk	Yes	No	Low risk
Emery et al, 2015 [53-55]	Permuted block randomization	Yes	Using a computer random number generator	Low risk	Central allocation*	Low risk	Yes	No	Low risk
Bae et al, 2016 [56]	Randomization with stratification	Yes	Using a computer random number generator	Low risk	Central allocation*	Low risk	Yes	No	Low risk
Odell et al, 2016 [57, 58]	Unclear	Unclear	Unclear	Unclear risk	Unclear	Unclear risk	Unclear	Unclear	Unclear risk
Matsuno et al, 2017 [59, 60]	Randomization with stratification	Unclear	Unclear	Unclear risk	Central allocation*	Low risk	Yes	No	Low risk

© 2023 de Oliveira Ascef B et al. *JAMA Network Open.*

Author, year	Random sequence generation				Allocation concealment		Inconsistent application of eligibility criteria		
	Method	Properly conducted	Tools	Overall judgement domain 1	Method	Overall judgement domain 2	Clearly stated and implemented	Participants selected for no/positive response	Overall judgement domain 3
Matucci-Cerinic et al, 2018 [61, 62]	Randomization with stratification	Yes	Using a computer random number generator	Low risk	Central allocation*	Low risk	Unclear	Unclear	Unclear risk
Yamanaka et al, 2020 [63]	Randomization with stratification	Yes	Using a computer random number generator	Low risk	Central allocation*	Low risk	Yes	No	Low risk
Strusberg et al, 2021 [64]	Permuted block randomization with stratification	Yes	Using a computer random number generator	Low risk	Central allocation*	Low risk	Yes	No	Low risk
Yoo et al, 2013 [65-67]	Simple randomization	Unclear	Unclear	Unclear risk	Unclear	Unclear risk	Yes	No	Low risk
Kay et al, 2014 [68, 69]	Unclear	Unclear	Unclear	Unclear risk	Unclear	Unclear risk	Unclear	Unclear	Unclear risk
Choe et al, 2015 [70-72]	Permuted block randomization	Yes	Using a computer random number generator	Low risk	Central allocation*	Low risk	Yes	No	Low risk
Takeuchi et al, 2015 [73]	Simple randomization	Unclear	Unclear	Unclear risk	Unclear	Unclear risk	Yes	No	Low risk
Matsuno et al, 2018 [74]	Randomization with stratification	Unclear	Unclear	Unclear risk	Central allocation*	Low risk	Yes	Unclear	Unclear risk
Lila et al, 2019 [75]	Permuted block randomization	Yes	Using a computer random	Low risk	Central allocation*	Low risk	Yes	Unclear	Unclear risk

© 2023 de Oliveira Ascef B et al. *JAMA Network Open.*

Author, year	Random sequence generation				Allocation concealment		Inconsistent application of eligibility criteria		
	**Method**	**Properly conducted**	**Tools**	**Overall judgement domain 1**	**Method**	**Overall judgement domain 2**	**Clearly stated and implemented**	**Participants selected for no/positive response**	**Overall judgement domain 3**
	with stratification		number generator						
Genovese et al, 2020 [76]	Randomization with stratification	Unclear	Unclear	Unclear risk	Unclear	Unclear risk	Yes	No	Low risk

*Central allocation (including telephone, web-based, and pharmacy-controlled randomization).

© 2023 de Oliveira Ascef B et al. *JAMA Network Open.*

eTable 6. Risk of Bias Assessment Domains: blinding of participants and investigator, and participant's behaviour changes.

	Blinding of participants and investigators					Participant's behaviour changes			
**Author, year**	**Participants were blinded**	**Staff were blinded**	**Outcome could be influenced by a lack of blinding**	**Blinding was broken during the trial**	**Overall judgement domain 4**	**Adequate treatment adherence**	**Potential confounders**	**Variation to the trial protocol**	**Overall judgement domain 5**
Jani et al, 2015 [29]	Yes	Yes	Yes	No	Low risk	Yes	No	No	Low risk
Alten et al, 2017 [30-36]	Yes	Yes	Yes	No	Low risk	Yes	No	No	Low risk
Cohen et al, 2017 [37, 38]	Unclear	Unclear	Yes	Unclear	Unclear risk	Yes	No	No	Low risk
Jamshidi et al, 2017 [39]	Yes	Yes	Yes	No	Low risk	Yes	No	No	Low risk
Fleishmann et al, 2018 [40-42]	Unclear	Unclear	Yes	Unclear	Unclear risk	Yes	No	No	Low risk
Cohen et al, 2018 [43, 44]	Unclear	Yes	Yes	Unclear	Unclear risk	Yes	No	No	Low risk
Weinblatt et al, 2018 [45, 46]	Yes	Yes	Yes	No	Low risk	Yes	No	No	Low risk
Edwards et al, 2019 [47]	Unclear	Unclear	Yes	No	Unclear risk	Yes	No	No	Low risk
Willand, et al 2019 [48, 49]	Yes	Unclear	Yes	Unclear	Unclear risk	Yes	No	Unclear	Unclear risk
Matsuno et al, 2021 [50]	Unclear	Unclear	Yes	Unclear	Unclear risk	Unclear	Unclear	Unclear	Unclear risk
Kay et al, 2021 [51, 52]	Yes	Yes	Yes	No	Low risk	Yes	No	No	Low risk
Emery et al, 2015 [53-55]	Yes	Yes	Yes	No	Low risk	No	No	No	High risk
Bae et al, 2016 [56]	Yes	Yes	Yes	No	Low risk	Yes	No	No	Low risk
Odell et al, 2016 [57, 58]	Unclear	Unclear	Yes	Unclear	Unclear risk	Unclear	Unclear	Unclear	Unclear risk
Matsuno et al, 2017 [59, 60]	Yes	Yes	Yes	No	Low risk	Yes	No	No	Low risk

© 2023 de Oliveira Ascef B et al. *JAMA Network Open.*

	Blinding of participants and investigators					Participant’s behaviour changes			
**Author, year**	**Participants were blinded**	**Staff were blinded**	**Outcome could be influenced by a lack of blinding**	**Blinding was broken during the trial**	**Overall judgement domain 4**	**Adequate treatment adherence**	**Potential confounders**	**Variation to the trial protocol**	**Overall judgement domain 5**
Matucci-Cerinic et al, 2018 [61, 62]	Yes	Yes	Yes	No	Low risk	Yes	No	No	Low risk
Yamanaka et al, 2020 [63]	Unclear	Unclear	Yes	Unclear	Unclear risk	Yes	No	No	Low risk
Strusberg et al, 2021 [64]	Unclear	Unclear	Yes	Unclear	Unclear risk	Yes	No	No	Low risk
Yoo et al, 2013 [65-67]	Unclear	Unclear	Yes	Unclear	Unclear risk	Yes	No	No	Low risk
Kay et al, 2014 [68, 69]	Unclear	Unclear	Yes	Unclear	Unclear risk	Unclear	Unclear	Unclear	Unclear risk
Choe et al, 2015 [70-72]	Yes	Yes	Yes	No	Low risk	Yes	No	No	Low risk
Takeuchi, et al 2015 [73]	Yes	Yes	Yes	No	Low risk	Yes	No	No	Low risk
Matsuno et al, 2018 [74]	Unclear	Unclear	Yes	Unclear	Unclear risk	Yes	No	No	Low risk
Lila et al, 2019 [75]	Yes	Yes	Yes	No	Low risk	No	No	No	High risk
Genovese et al, 2020 [76]	Unclear	Unclear	Yes	Unclear	Unclear risk	Yes	No	No	Low risk

© 2023 de Oliveira Ascef B et al. *JAMA Network Open.*

eTable 7. Risk of Bias Assessment Domains: blinding of outcome assessors, and outcomes measures.

Author, year	Blinding of outcome assessors		Outcomes measures		
	Outcome assessors were blinded (ACR/HAQ-DI)	Overall judgement domain 6	Validated instrument	Data collection methods were appropriate	Overall judgement domain 7
Jani et al, 2015 [29]	Unclear	Unclear risk	Yes	Yes	Low risk
Alten et al, 2017 [30-36]	Unclear	Unclear risk	Yes	Yes	Low risk
Cohen et al, 2017 [37, 38]	Unclear	Unclear risk	Yes	Yes	Low risk
Jamshidi et al, 2017 [39]	Unclear	Unclear risk	Yes	Yes	Low risk
Fleishmann et al, 2018 [40-42]	Unclear	Unclear risk	Yes	Yes	Low risk
Cohen et al, 2018 [43, 44]	Unclear	Unclear risk	Yes	Yes	Low risk
Weinblatt et al, 2018 [45, 46]	Yes	Low risk	Yes	Yes	Low risk
Edwards et al, 2019 [47]	Unclear	Unclear risk	Yes	Yes	Low risk
Willand et al, 2019 [48, 49]	Unclear	Unclear risk	Yes	Yes	Low risk
Matsuno et al, 2021 [50]	Unclear	Unclear risk	Unclear	Unclear	Unclear risk
Kay et al, 2021 [51, 52]	Yes	Low risk	Yes	Yes	Low risk
Emery et al, 2015 [53-55]	Yes	Low risk	Yes	Yes	Low risk
Bae et al, 2016 [56]	Yes	Low risk	Yes	Yes	Low risk
Odell et al, 2016 [57, 58]	Unclear	Unclear risk	Unclear	Unclear	Unclear risk
Matsuno et al, 2017 [59, 60]	Yes	Low risk	Yes	Yes	Low risk
Matucci-Cerinic et al, 2018 [61, 62]	Yes	Low risk	Yes	Yes	Low risk
Yamanaka et al, 2020 [63]	Unclear	Unclear risk	Yes	Unclear	Low risk

© 2023 de Oliveira Ascef B et al. *JAMA Network Open.*

Author, year	Blinding of outcome assessors		Outcomes measures		
	Outcome assessors were blinded (ACR/HAQ-DI)	Overall judgement domain 6	Validated instrument	Data collection methods were appropriate	Overall judgement domain 7
Strusberg et al, 2021 [64]	Yes	Low risk	Yes	Yes	Low risk
Yoo et al, 2013 [65-67]	Unclear	Unclear risk	Yes	Yes	Low risk
Kay et al, 2014 [68, 69]	Unclear	Unclear risk	Unclear	Unclear	Unclear risk
Choe et al, 2015 [70-72]	Yes	Low risk	Yes	Yes	Low risk
Takeuchi et al, 2015 [73]	Yes	Low risk	Yes	Yes	Low risk
Matsuno et al, 2018 [74]	Unclear	Unclear risk	Yes	Yes	Low risk
Lila et al, 2019 [75]	Yes	Low risk	Yes	Yes	Low risk
Genovese et al, 2020 [76]	Unclear	Unclear risk	Yes	Yes	Low risk

Abbreviations: ACR, the American College of Rheumatology; SDAI, Simplified Disease Activity Score; CDAI, Clinical Disease Activity Score; HAQ-DI, Health Assessment Questionnaire - Disability Index.

© 2023 de Oliveira Ascef B et al. *JAMA Network Open.*

eTable 8. Risk of Bias Assessment Domain: incomplete outcome data.

	ACR20			HAQ-DI			Overall judgement domain 8 [b]
	Overall attrition [a]		Analysis set	Overall attrition [a]		Analysis set	
Author, year	PP	mITT/ITT		PP	mITT/ITT		
Jani et al, 2015 [29]	Yes	No	Both mITT and PP	Yes	No	Both ITT and PP	Low risk
Alten et al, 2017 [30-36]	NA	No	mITT only	NA	No	mITT only	High risk
Cohen et al, 2017 [37, 38]	NA	No	mITT only	NA	NA	NA	High risk
Jamshidi et al, 2017 [39]	No	NA	PP only	Yes	No	PP only	Low risk
Fleishmann et al, 2018 [40-42]	Yes	No	Both mITT and PP	Yes	No	Both mITT and PP	Low risk
Cohen et al, 2018 [43, 44]	Yes	No	Both mITT and PP	NA	NA	NA	Low risk
Weinblatt et al, 2018 [45, 46]	Yes	No	Both mITT and PP	NA	NA	NA	Low risk
Edwards et al, 2019 [47]	Yes	No	Both ITT and PP	Yes	No	Both ITT and PP	Low risk
Willand et al, 2019 [48, 49]	Yes	NA	PP only	Yes	NA	PP only	High risk
Matsuno et al, 2021 [50]	NA	NA	NA	NA	NA	NA	Unclear risk
Kay et al, 2021 [51, 52]	Yes	No	Both ITT and PP	NA	NA	NA	Low risk
Emery et al, 2015 [53-55]	Yes	No	Both mITT and PP	NA	No	ITT only	High risk
Bae et al, 2016 [56]	Yes	Yes	Both mITT and PP	Yes	NA	PP only	High risk
Odell et al, 2016 [57, 58]	Yes	NA	PP only	Yes	NA	PP only	High risk
Matsuno et al, 2017 [59, 60]	NA	No	mITT only	NA	NA	NA	High risk
Matucci-Cerinic et al, 2018 [61, 62]	Yes	No	PP only	Yes	NA	PP only	High risk
Yamanaka et al, 2020 [63]	No	No	Both mITT and PP	NA	NA	NA	Low risk
Strusberg et al, 2021 [64]	Yes	No	Both ITT and PP	NA	Yes	mITT only	High risk
Yoo et al, 2013 [65-67]	Yes	No	Both ITT and PP	Yes	NA	PP only	High risk
Kay et al, 2014 [68, 69]	NA	No	ITT only	NA	NA	NA	High risk
Choe et al, 2015 [70-72]	Yes	No	Both mITT and PP	NA	No	ITT only	High risk
Takeuchi et al, 2015 [73]	NA	No	mITT only	NA	No	mITT only	High risk
Matsuno et al, 2018 [74]	Yes	NA	PP only	NA	NA	NA	High risk
Lila et al, 2019 [75]	NA	No	mITT only	NA	NA	NA	High risk
Genovese et al, 2020 [76]	NA	No	ITT only	NA	NA	NA	High risk

[a] Overall attrition: A loss greater than 5% in any of the groups and/or an absolute difference greater than 5% between groups was considered a substantial risk of attrition bias.

[b] If the study reported their results with ITT/mITT and PP; and mITT missing data was greater than 5% in any of the groups and/or an absolute difference greater than 5% between groups, we considered it to be at high risk of bias; or If the study reported their results with only ITT/mITT; and mITT missing data was greater than 5% in any of the groups and/or an absolute difference greater than 5% between groups, we considered it to be at high risk of bias; or If the study reported their results with only ITT/mITT independently of the percentage of missing data, we considered it to be at high risk of bias; or If the study reported their results with only PP; and PP missing data was greater than 5% in any of the groups and/or an absolute difference greater than 5% between groups, we considered it to be at high risk of bias; and If any of the assumptions described previously are valid for the primary outcome (ACR20) and/or the co-primary-outcome (HAQ-DI), we considered it to be at high risk of bias.

Abbreviations: ACR20, the American College of Rheumatology 20 criteria; HAQ-DI, Health Assessment Questionnaire - Disability Index; NA: not available; PP, per protocol analysis (PP); mITT, modified Intention-to-treat analysis; ITT, intention-to-treat analysis.

© 2023 de Oliveira Ascef B et al. *JAMA Network Open.*

eTable 9. Effects of biosimilars and biologics on ACR20 response: Bayesian random-effects meta-analysis.

	No. Trials	No. Patients	RR	95% CrI	95% PI	Pr (E) (%)	τ^2
**ACR20 1M**							
Bios vs. Ref	15	7430	1.034	0.980, 1.089	0.913, 1.167	83.7	0.001
ADA	8	4135	1.025	0.945, 1.121	0.851, 1.249	77.2	0.003
ETN	4	1965	1.025	0.793, 1.328	0.617, 1.724	65.5	0.003
IFX	3	1330	1.012	0.616, 1.604	0.383, 2.570	44.7	0.018
**ACR20 3M**							
Bios vs. Ref	23	9332	1.027	0.999, 1.054	0.969, 1.090	99.0	<0.001
ADA	10	4243	1.021	0.973, 1.076	0.910, 1.155	92.7	0.001
ETN	6	2527	1.030	0.976, 1.090	0.931, 1.145	85.9	0.001
IFX	7	2562	1.034	0.965, 1.111	0.965, 1.111	76.7	0.002
**ACR20 6M**							
Bios vs. Ref	24	9225	1.006	0.985, 1.040	0.949, 1.072	100	0.000
ADA	10	4215	1.003	0.969, 1.040	0.933, 1.082	99.6	<0.001
ETN	7	2533	0.992	0.946, 1.042	0.885, 1.114	97.4	0.001
IFX	7	2477	1.055	0.988,1.134	0.914, 1.229	55.5	0.001
**ACR20 12M**							
Bios vs. Ref	8	2727	1.014	0.964, 1.072	0.908, 1.141	94.6	0.001
ADA	1	242	0.948	0.104, 8.771	NA	NA	NA
ETN	3	943	1.025	0.793, 1.328	0.617, 1.724	65.5	0.003
IFX	4	1543	1.034	0.847, 1.341	0.653, 1.742	56.1	0.008

RR comparing Bios and Ref on ACR20 response by timepoint and type of reference molecule.

Abbreviations: ACR20, the American College of Rheumatology 20 criteria; RR, relative risk; Bios, biosimilars; Ref, reference biologics drugs; ADA, adalimumab; ETN, etanercept; IFX, infliximab; M, months; CrI, credible interval; PI, predictive interval; Pr (E), the posterior probability of equivalence based on the pre-specified margins of equivalence [0.94, 1.06], τ^2: Tau squared estimates between-study heterogeneity; NA, not applicable.

© 2023 de Oliveira Ascef B et al. *JAMA Network Open.*

eTable 10. Effects of biosimilars and biologics on ACR20 response: Frequentist fixed-effects meta-analysis.

	No. Trials	No. Patients	RR	95% CI	*P* values	I^2 (%)	*P* values
**ACR20 1M**							
Bios vs. Ref	15	7430	1.039	0.994, 1.087	0.09	0.0	0.46
ADA	8	4135	1.026	0.964, 1.092	0.42	2.8	0.41
ETN	4	1965	1.065	0.979, 1.159	0.14	31.5	0.22
IFX	3	1330	1.038	0.940, 1.147	0.42	0.0	0.46
**ACR20 3M**							
Bios vs. Ref	23	9332	1.029	**1.004, 1.055**	**0.02**	0.0	0.84
ADA	10	4243	1.019	0.982, 1.058	0.31	13.1	0.32
ETN	6	2527	1.030	0.987, 1.076	0.18	0.0	0.93
IFX	7	2562	1.046	0.992, 1.103	0.09	0.0	0.77
**ACR20 6M**							
Bios vs. Ref	24	9225	1.005	0.986, 1.024	0.61	10.5	0.31
ADA	10	4215	1.003	0.975, 1.032	0.83	0.0	0.69
ETN	7	2533	0.991	0.964, 1.019	0.53	33.9	0.17
IFX	7	2477	1.055	**1.004, 1.109**	**0.04**	0.0	0.47
**ACR20 12M**							
Bios vs. Ref	8	2727	1.015	0.976, 1.055	0.45	0.0	0.44
ADA	1	242	0.947	0.849, 1.056	0.33	NA	NA
ETN	3	943	1.027	0.975, 1.082	0.32	0.0	0.64
IFX	4	1543	1.023	0.954, 1.096	0.53	28.6	0.24

RR comparing Bios and Ref on ACR20 response by timepoint and type of reference molecule.

Abbreviations: ACR20, the American College of Rheumatology 20 criteria; RR, relative risk; Bios, biosimilars; Ref, reference biologics drugs; ADA, adalimumab; ETN, etanercept; IFX, infliximab; M, months; CI, confidence interval; I2 metric estimates within-study variance; NA, not applicable.

© 2023 de Oliveira Ascef B et al. *JAMA Network Open.*

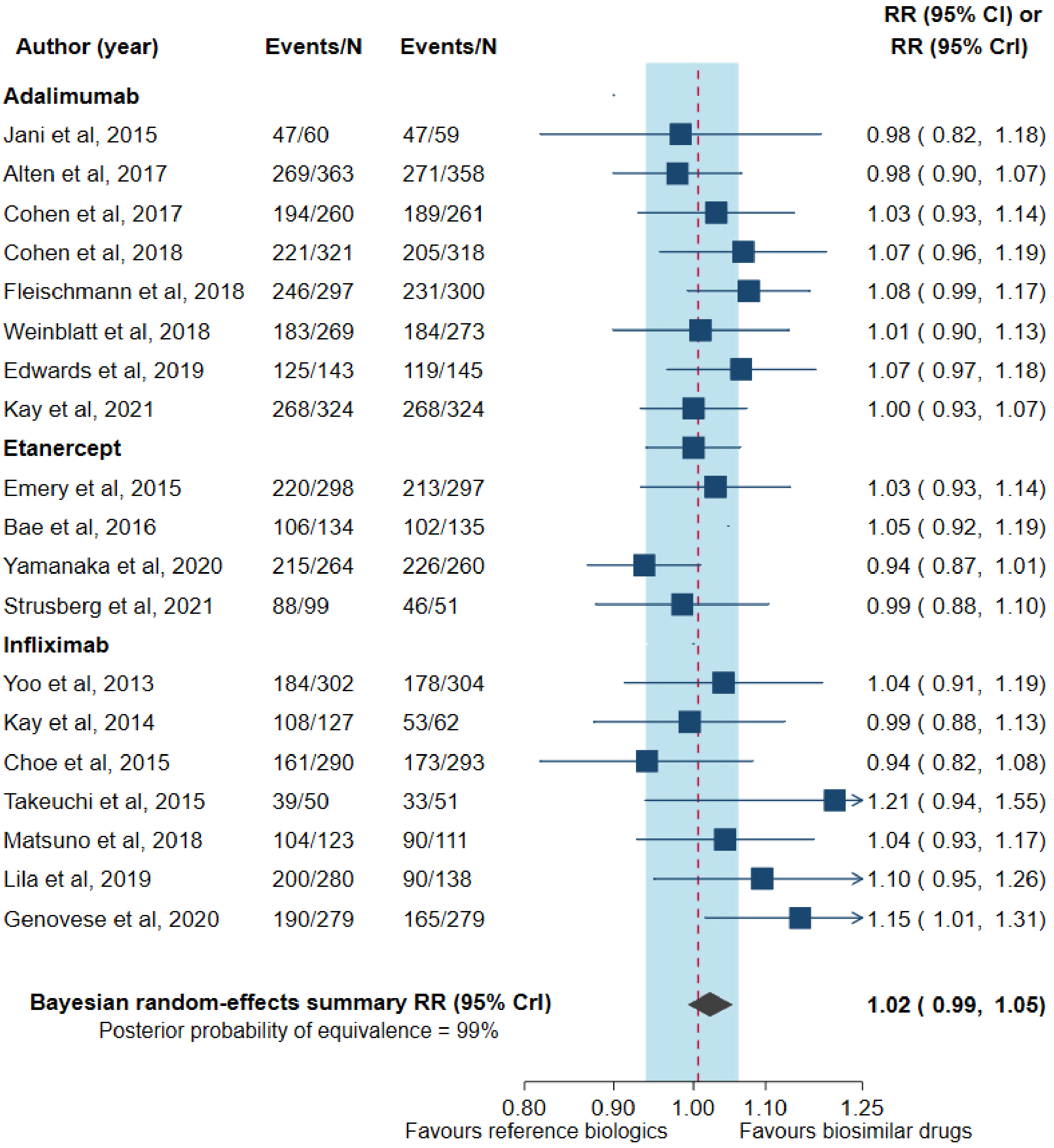

eFigure 4. Non-prespecified and exploratory analyses of ACR20 including only studies reporting modified intention-to-treat or intention-to-treat analyses.

Abbreviations: ACR20, the American College of Rheumatology 20 criteria; RR, relative risk; Bios, biosimilars; Ref, reference biologics drugs; ADA, adalimumab; ETN, etanercept; IFX, infliximab; M, months; CrI, credible interval; PI, predictive interval; Pr (E), the posterior probability of equivalence based on the pre-specified margins of equivalence [0.94, 1.06].

© 2023 de Oliveira Ascef B et al. *JAMA Network Open.*

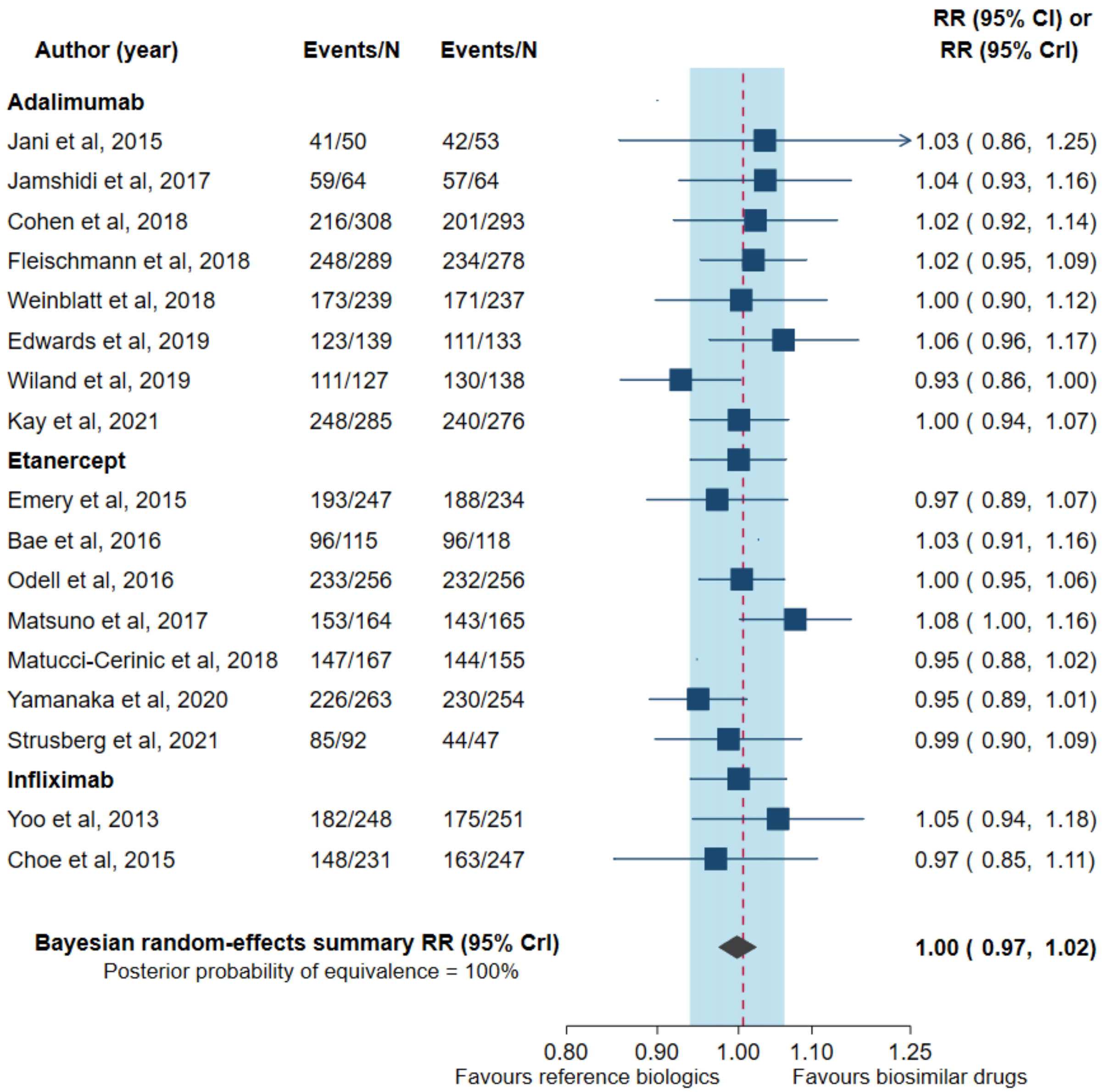

eFigure 5. Non-prespecified and exploratory analyses of ACR20 including only studies reporting per-protocol analyses.

Abbreviations: ACR20, the American College of Rheumatology 20 criteria; RR, relative risk; Bios, biosimilars; Ref, reference biologics drugs; ADA, adalimumab; ETN, etanercept; IFX, infliximab; M, months; CrI, credible interval; PI, predictive interval; Pr (E), the posterior probability of equivalence based on the pre-specified margins of equivalence [0.94, 1.06].

© 2023 de Oliveira Ascef B et al. *JAMA Network Open.*

eTable 11. Effects of biosimilars and biologics on HAQ-DI: Bayesian random-effects meta-analysis.

	No. Trials	No. Patients	SMD	95% CrI	95% PI	Pr (E) (%)	τ^2
**HAQ-DI 1M**							
Bios vs. Ref	6	2384	0.045	-0.081, 0.168	-0.221, 0.308	99.8	0.005
ADA	3	956	0.041	-0.626, 0.677	-1.269, 1.329	92.0	0.020
ETN	3	1428	0.049	-0.730, 0.835	-1.496, 1.628	91.1	0.039
IFX	NR	NR	NR	NR	NA	NA	NA
**HAQ-DI 3M**							
Bios vs. Ref	10	3394	-0.031	-0.111, 0.047	-0.180, 0.116	100.0	0.001
ADA	5	1364	-0.011	-0.182, 0.148	-0.343, 0.307	98.6	0.005
ETN	3	1430	-0.011	-0.548, 0.539	-1.082, 1.087	92.7	0.012
IFX	2	600	-0.119	-2.930, 2.732	-5.022, 4.801	63.7	0.401
**HAQ-DI 6M**							
Bios vs. Ref	14	5042	-0.041	-0.109, 0.024	-0.190, 0.101	100.0	0.002
ADA	6	2055	-0.017	-0.139, 0,107	-0.256, 0.225	99.6	0.003
ETN	5	1803	-0.049	-0.332, 0.199	-0.690, 0.556	92.9	0.024
IFX	3	1184	-0.098	-0.900, 0.661	-1.664, 1.451	79.1	0.036
**HAQ-DI 12M**							
Bios vs. Ref	6	2199	-0.043	-0.278, 0.140	-0.579, 0.437	95.1	0.019
ADA	1	239	-0.000	-6.284, 6.282	NA	NA	NA
ETN	2	829	0.079	-2.615, 2.770	-4.568, 4.722	78.7	0.266
IFX	3	1131	-0.174	-1.559, 1.111	-2.892, 2.415	57.9	0.175

SMD in the change in HAQ-DI scores between Bios and Ref by timepoint and type of reference molecule.

Abbreviations: SMD, Standardised Mean Differences; HAQ-DI, Health Assessment Questionnaire-Disability Index; Bios, biosimilars drugs; Ref, reference biologics drugs; ADA, adalimumab; ETN, etanercept; IFX, infliximab; M, months; CrI, credible interval; PI, predictive interval; Pr (E), the posterior probability of equivalence based on the pre-specified margins of equivalence (SMD scale: [-0.22, 0.22] standard deviations); τ^2: Tau squared estimates between-study heterogeneity; NR, not reported; NA, not applicable.

© 2023 de Oliveira Ascef B et al. *JAMA Network Open.*

eTable 12. Effects of biosimilars and biologics on HAQ-DI: Frequentist fixed-effects meta-analysis.

	No. Trials	No. Patients	SMD	95% CI	*P* values	I^2 (%)	*P* values
**HAQ-DI 1M**							
Bios vs. Ref	6	2384	0.046	-0.035, 0.126	0.26	0.0	0.54
ADA	3	956	0.049	-0.078, 0.176	0.45	0.0	0.84
ETN	3	1428	0.044	-0.060, 0.410	0.51	45.6	0.16
IFX	NR	NR	NR	NR	NA	NA	NA
**HAQ-DI 3M**							
Bios vs. Ref	10	3394	-0.032	-0.099, 0.035	0.35	0.0	0.93
ADA	5	1364	-0.013	-0.119, 0.093	0.81	0.0	0.92
ETN	3	1430	-0.015	-0.119, 0.088	0.77	0.0	0.69
IFX	2	600	-0.130	-0.291, 0.030	0.11	0.0	0.73
**HAQ-DI 6M**							
Bios vs. Ref	14	5042	-0.041	-0.096, 0.015	0.15	1.3	0.43
ADA	6	2055	-0.025	-0.111, 0.062	0.57	0.0	0.94
ETN	5	1803	-0.040	-0.133, 0.052	0.39	50.8	0.09
IFX	3	1184	-0.084	-0.198, 0.030	0.15	6.4	0.34
**HAQ-DI 12M**							
Bios vs. Ref	6	2199	-0.023	-0.107, 0.060	0.59	52.8	0.06
ADA	1	239	0.000	-0.254, 0.254	NA	NA	NA
ETN	2	829	0.081	-0.055, 0.217	0.24	0.0	0.91
IFX	3	1131	-0.105	-0.222, 0.012	0.08	68.8	0.04

SMD in the change in HAQ-DI scores between Bios and Ref by timepoint and type of reference molecule.

Abbreviations: SMD, Standardised Mean Differences; HAQ-DI, Health Assessment Questionnaire-Disability Index; Bios, biosimilars drugs; Ref, reference biologics drugs; ADA, adalimumab; ETN, etanercept; IFX, infliximab; M, months; CI, confidence interval; I^2 metric estimates within-study variance; NR, not reported; NA, not applicable.

© 2023 de Oliveira Ascef B et al. *JAMA Network Open.*

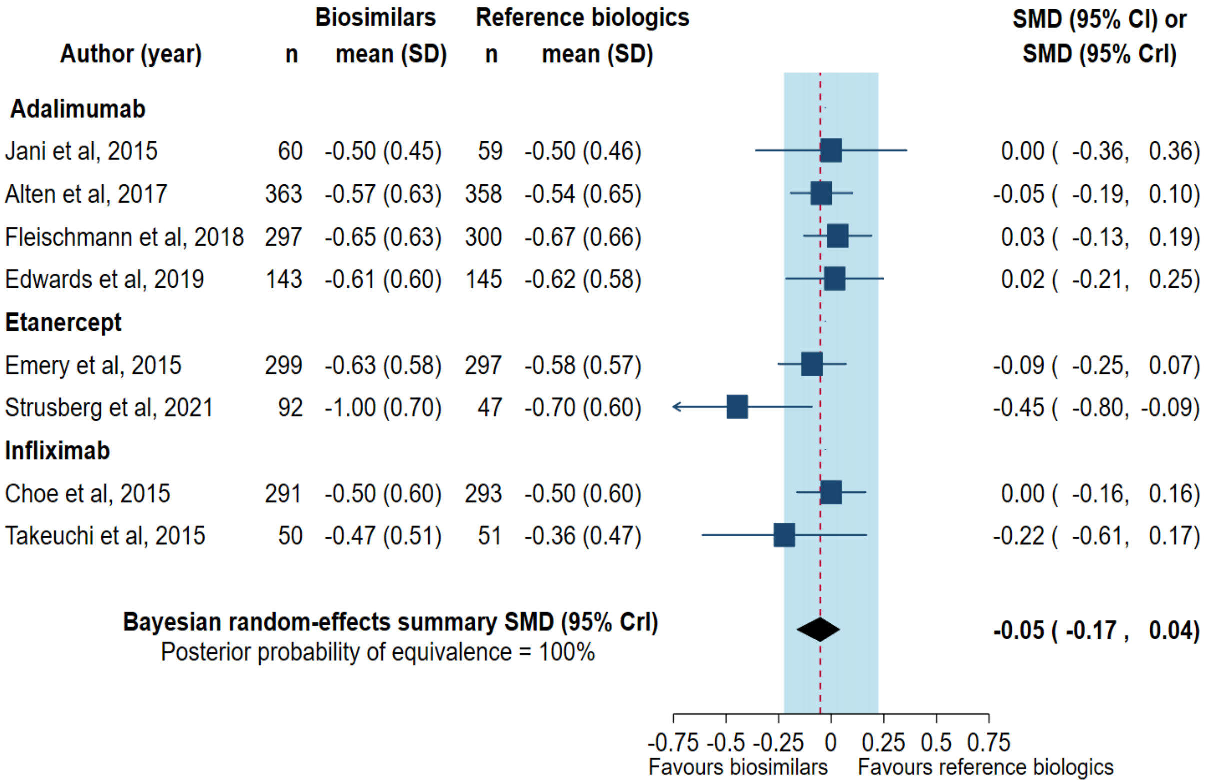

eFigure 6. Non-prespecified and exploratory analyses of HAQ-DI including only studies reporting modified intention-to-treat or intention-to-treat analyses.

Abbreviations: SMD, Standardised Mean Differences; HAQ-DI, Health Assessment Questionnaire-Disability Index; Bios, biosimilars drugs; Ref, reference biologics drugs; ADA, adalimumab; ETN, etanercept; IFX, infliximab; M, months; CrI, credible interval; PI, predictive interval; Pr (E), the posterior probability of equivalence based on the pre-specified margins of equivalence (SMD scale: [-0.22, 0.22] standard deviations).

© 2023 de Oliveira Ascef B et al. *JAMA Network Open.*

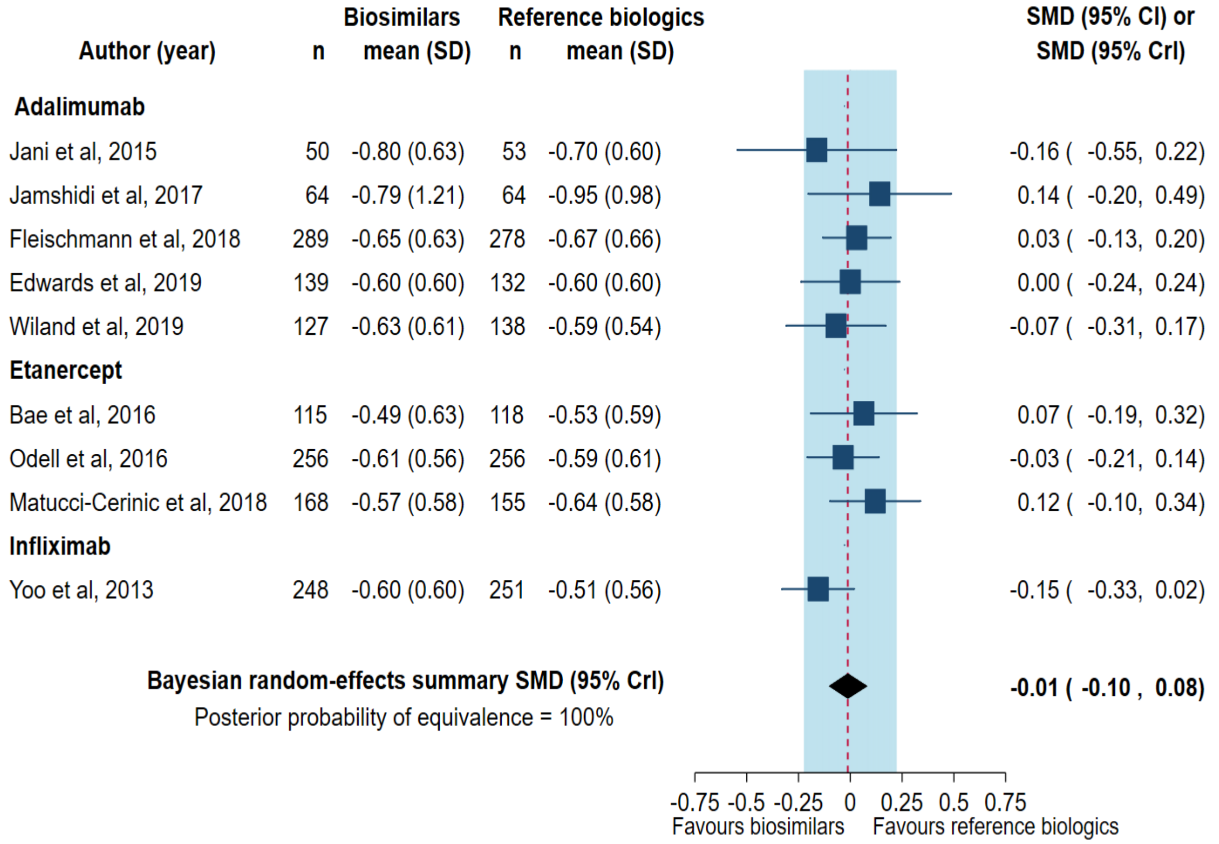

eFigure 7. Non-prespecified and exploratory analyses of ACR20 including only studies reporting per-protocol analyses.

Abbreviations: SMD, Standardised Mean Differences; HAQ-DI, Health Assessment Questionnaire-Disability Index; Bios, biosimilars drugs; Ref, reference biologics drugs; ADA, adalimumab; ETN, etanercept; IFX, infliximab; M, months; CrI, credible interval; PI, predictive interval; Pr (E), the posterior probability of equivalence based on the pre-specified margins of equivalence (SMD scale: [-0.22, 0.22] standard deviations).

© 2023 de Oliveira Ascef B et al. *JAMA Network Open.*

eTable 13. Effects of biosimilars and biologics on ACR50 and ACR70: Bayesian random-effects meta-analysis.

	No. Trials	No. Patients	RR	95% CrI	95% PI	$τ^2$
**ACR50 1M**						
Bios vs. Ref	12	6118	0.975	0.758, 1.257	0.758, 1.257	0.005
ADA	7	3659	1.019	0.859, 1.219	0.738, 1.425	0.006
ETN	3	1318	0.943	0.444, 1.952	0.213, 4.035	0.079
IFX	2	1141	0.873	0.213, 3.459	0.072, 10.380	0.408
**ACR50 3M**						
Bios vs. Ref	20	8142	1.001	0.946, 1.059	0.885, 1.132	0.001
ADA	9	3869	0.993	0.909, 1.085	0.836, 1.184	0.002
ETN	5	1180	1.006	0.789, 1.268	0.581, 1.727	0.019
IFX	6	2393	1.010	0.853, 1.177	0.710, 1.406	0.007
**ACR50 6M**						
Bios vs. Ref	21	8872	1.011	0.964, 1.065	0.867, 1.185	0.004
ADA	9	4190	0.985	0.920, 1.052	0.859, 1.127	0.001
ETN	6	2394	1.031	0.862, 1.247	0.651, 1.653	0.025
IFX	6	2288	1.060	0.929, 1.210	0.808, 1.388	0.004
**ACR50 12M**						
Bios vs. Ref	8	2742	1.081	0.988, 1.191	0.889, 1.328	0.003
ADA	1	241	0.972	0.106, 8.974	NA	NA
ETN	3	958	1.146	0.764, 1.737	0.508, 2.591	0.011
IFX	4	1543	1.058	0.802, 1.523	0.558, 2.186	0.014
**ACR70 1M**						
Bios vs. Ref	12	6118	0.907	0.731, 1.138	0.607, 1.361	0.011
ADA	7	3659	0.864	0.593, 1.269	0.418, 1.830	0.033
ETN	3	1318	1.110	0.464, 2.542	0.211, 5.597	0.097
IFX	2	1141	0.815	0.218, 3.019	0.080, 8.142	0.247
**ACR70 3M**						
Bios vs. Ref	20	8142	1.013	0.914, 1.118	0.800, 1.270	0.004
ADA	9	3869	0.979	0.819, 1.160	0.666, 1.404	0.010
ETN	5	1880	0.996	0.728, 1.322	0.518, 1.853	0.019
IFX	6	2393	1.079	0.784, 1.439	0.537, 2.093	0.032
**ACR70 6M**						
Bios vs. Ref	21	8856	0.975	0.906, 1.048	0.813, 1.169	0.003
ADA	9	4174	0.909	0.811, 1.013	0.731, 1.127	0.003
ETN	6	2394	1.013	0.831, 1.229	0.641, 1.593	0.017
IFX	6	2288	1.070	0.859, 1.338	0.674, 1.711	0.013
**ACR70 12M**						
Bios vs. Ref	6	2393	1.091	0.934, 1.315	0.734, 1.697	0.012
ADA	1	241	0.930	0.102, 8.725	NA	NA
ETN	3	958	1.155	0.701, 1.883	0.435, 3.052	0.019
IFX	4	1543	1.146	0.565, 2.708	0.216, 7.001	0.236

RR comparing Bios and Ref on ACR50 and ACR70 responses by timepoint and type of reference molecule.

Abbreviations: ACR50, The American College of Rheumatology 50 criteria; ACR70, The American College of Rheumatology 70 criteria; RR, relative risk; Bios, biosimilars; Ref, reference biologics drugs; ADA, adalimumab; ETN, etanercept; IFX, Infliximab; M, months; CrI, credible interval; PI, predictive interval; $τ^2$, Tau squared estimates between-study heterogeneity; NA, not applicable.

© 2023 de Oliveira Ascef B et al. *JAMA Network Open.*

eTable 14. Effects of biosimilars and biologics on ACR50 and ACR70: Frequentist fixed-effects meta-analysis.

	No. Trials	No. Patients	RR	95% CI	*P* values	I^2 (%)	*P* values
**ACR50 1M**							
Bios vs. Ref	12	6118	0.996	0.897, 1.106	0.94	0.0	0.70
ADA	7	3659	1.037	0.899, 1.197	0.62	0.0	0.90
ETN	3	1318	0.966	0.778, 1.199	0.75	2.8	0.36
IFX	2	1141	0.937	0.753, 1.165	0.56	68.6	0.07
**ACR50 3M**							
Bios vs. Ref	20	8142	1.009	0.958, 1.063	0.74	0.0	0.65
ADA	9	3869	0.998	0.926, 1.075	0.95	0.0	0.76
ETN	5	1180	1.016	0.922, 1.121	0.75	42.9	0.13
IFX	6	2393	1.024	0.920, 1.140	0.66	0.0	0.58
**ACR50 6M**							
Bios vs. Ref	21	8872	1.010	0.973, 1.049	0.60	29.8	0.10
ADA	9	4190	0.988	0.935, 1.043	0.65	0.0	0.75
ETN	6	2394	1.018	0.956, 1.084	0.57	72.2	0.003
IFX	6	2288	1.060	0.965, 1.165	0.22	0.0	0.60
**ACR50 12M**							
Bios vs. Ref	8	2742	1.084	1.011, 1.162	**0.02**	0.0	0.43
ADA	1	241	0.975	0.812, 1.171	0.79	NA	NA
ETN	3	958	1.146	1.039, 1.265	**0.007**	0.0	0.63
IFX	4	1543	1.047	0.932, 1.176	0.44	4.9	0.37
**ACR70 1M**							
Bios vs. Ref	12	6118	0.957	0.789, 1.160	0.65	0.0	0.91
ADA	7	3659	0.919	0.682, 1.239	0.77	0.0	0.58
ETN	3	1318	1.189	0.813, 1.741	0.37	0.0	0.92
IFX	2	1141	0.848	0.605, 1.189	0.34	0.0	0.80
**ACR70 3M**							
Bios vs. Ref	20	8142	1.039	0.948, 1.138	0.42	0.0	0.68
ADA	9	3869	1.009	0.885, 1.150	0.89	0.0	0.59
ETN	5	1880	1.022	0.860, 1.215	0.80	0.0	0.60
IFX	6	2393	1.122	0.931, 1.353	0.23	9.1	0.36
**ACR70 6M**							
Bios vs. Ref	21	8856	0.984	0.922, 1.049	0.61	0.0	0.47
ADA	9	4174	0.918	0.837, 1.008	0.07	0.0	0.81
ETN	6	2394	1.026	0.921, 1.143	0.65	31.1	0.20
IFX	6	2288	1.092	0.935, 1.275	0.27	0.0	0.61
**ACR70 12M**							
Bios vs. Ref	6	2393	1.122	0.931, 1.353	0.23	9.1	0.39
ADA	1	241	0.936	0.690, 1.270	0.67	NA	NA
ETN	3	958	1.169	1.008, 1.357	**0.04**	0.0	0.94
IFX	4	1543	1.036	0.874, 1.228	0.68	65.3	0.03

RR comparing Bios and Ref on ACR50 and ACR70 responses by timepoint and type of reference molecule.

Abbreviations: ACR50, The American College of Rheumatology 50 criteria; ACR70, The American College of Rheumatology 70 criteria; RR, relative risk; Bios, biosimilars; Ref, reference biologics drugs; ADA, adalimumab; ETN, etanercept; IFX, infliximab; M, months; CI, confidence interval; I^2 metric estimates within-study variance; NA, not applicable.

© 2023 de Oliveira Ascef B et al. *JAMA Network Open.*

eTable 15. Frequency of safety outcomes with uncommon events (meta-analysis not performed).

Outcome	No. Trials	no. events/No. total (%) biosimilars	no. events/No. total (%) reference biologics
Mortality	21	8/4906 (0.16)	11/4751 (0.23)
Mortality related to treatment	19	1/4548 (0.02)	1/4390 (0.02)
Serious infections	10	21/2310 (0.90)	26/2160 (1.20)
Active tuberculosis	13	7/2992 (0.23)	5/2803 (0.17)
Malignancies	15	18/3907 (0.46)	22/3755 (0.58)

© 2023 de Oliveira Ascef B et al. *JAMA Network Open.*

eTable 16. Effects of biosimilars and reference biologics on safety and immunogenicity outcomes (Bayesian random-effects meta-analysis).

	No. Trials	No. Patients	RR	95% CrI	95% PI	$τ^2$
**SAFETY OUTCOMES**						
**Overall TEAE**						
Bios vs. Ref	23	9586	0.900	**0.836, 0.958**	0.717, 1.111	0.007
ADA	10	4532	0.878	**0.753, 0.990**	0.603, 1.230	0.012
ETN	6	2360	0.791	**0.652, 0.962**	0.514, 1.222	0.013
IFX	7	2694	0.986	0.893, 1.091	0.806, 1.213	0.002
**Serious TEAE**						
Bios vs. Ref	21	9021	0.949	0.767, 1.171	0.612, 1.455	0.024
ADA	9	3936	0.684	0.454, 1.008	0.312, 1.449	0.034
ETN	6	2580	0.995	0.593, 1.636	0.359, 2.704	0.048
IFX	6	2505	1.029	0.651, 1.654	0.380, 2.855	0.063
**Hypersensitivity**						
Bios vs. Ref	4	2058	0.726	0.388, 1.317	0.254, 1.958	0.082
**IRRs**						
Bios vs. Ref	6	2505	0.806	0.559, 1.192	0.405, 1.634	0.052
IFX	6	2505	0.806	0.559, 1.192	0.405, 1.634	0.052
**ISRs**						
Bios vs. Ref	15	6772	0.460	**0.326, 0.650**	0.141, 1.374	0.305
ADA	8	3816	0.631	**0.415, 0.953**	0.234, 1.695	0.102
ETN	7	2956	0.329	**0.162, 0.641**	0.050, 2.054	0.521
**Overall discontinuation**						
Bios vs. Ref	23	10069	0.876	0.765, 1.005	0.611, 1.248	0.017
ADA	10	4587	0.928	0.696, 1.285	0.437, 2.067	0.063
ETN	7	2962	0.781	0.440, 1.264	0.194, 2.848	0.194
IFN	6	2520	0.839	0.661, 1.059	0.515, 1.354	0.012
**IMMUNOGENICITY OUTCOMES**						
**ADAs**						
Bios vs. Ref	24	10151	0.684	0.467, 0.972	0.124, 3.624	0.620
ADA	10	4532	0.969	0.864, 1.076	0.732, 1.266	0.009
ETN	7	2955	0.205	**0.072, 0.609**	0.013, 3.442	1.407
IFN	7	2633	1.005	0.817, 1.174	0.636, 1.491	0.012
**NAbs**						
Bios vs. Ref	18	NA*	1.036	0.984, 1.093	0.933, 1.146	0.001
ADA	8	NA*	1.001	0.905, 1.099	0.830, 1.195	0.002
ETN	5	NA*	0.457	0.043, 2.312	0.015, 6.080	0.713
IFN	5	NA*	1.075	0.914, 1.717	0.662, 2.379	0.005

* The denominators of RR were based on the number of patients with ADAs.

RR comparing Bios and Ref on safety and immunogenicity outcomes by type of reference molecule.

Abbreviations: TEAE, treatment-emergent adverse events; IRRs, infusion-related reactions; ISRs, injection site reactions; ADAs, positive anti-drug antibodies; NAbs, positive neutralizing antibodies; RR, relative risk; Bios, biosimilars; Ref, reference biologics drugs; ADA, adalimumab; ETN, etanercept; IFX, infliximab; M, months; CrI, credible interval; PI, predictive interval; $τ^2$, Tau squared estimates between-study heterogeneity; NA, not applicable.

© 2023 de Oliveira Ascef B et al. *JAMA Network Open.*

eTable 17. Effects of biosimilars and reference biologics on safety and immunogenicity outcomes (Frequentist fixed-effects meta-analysis).

	No. Trials	No. Patients	RR	95% CI	*P* values	I^2 (%)	*P* values
**SAFETY OUTCOMES**							
**Overall TEAE**							
Bios vs. Ref	23	9586	0.900	**0.857, 0.945**	**<0.001**	33.8	0.06
ADA	10	4532	0.891	**0.828, 0.958**	**0.002**	34.6	0.13
ETN	6	2360	0.799	**0.788, 0.887**	**<0.001**	5.4	0.38
IFX	7	2694	0.988	0.913, 1.070	0.77	0.0	0.74
**Serious TEAE**							
Bios vs. Ref	21	9021	0.987	0.818, 1.191	0.89	0.0	0.84
ADA	9	3936	0.768	0.552, 1.069	0.12	0.0	0.87
ETN	6	2580	1.142	0.800, 1.631	0.46	0.0	0.75
IFX	6	2505	1.107	0.819, 1.496	0.51	0.0	0.49
**Hypersensitivity**							
Bios vs. Ref	4	2058	0.747	0.487, 1.145	0.18	19.3	0.29
**IRRs**							
Bios vs. Ref	6	2505	0.792	0.608, 1.032	0.08	0.0	0.45
IFN	6	2505	0.792	0.608, 1.032	0.08	0.0	0.45
**ISRs**							
Bios vs. Ref	15	6772	0.472	**0.404, 0.553**	**<0.001**	81.5	<0.001
ADA	8	3816	0.679	**0.518, 0.890**	**0.005**	22.1	0.25
ETN	7	2956	0.384	**0.316, 0.467**	**<0.001**	91.9	<0.001
**Overall discontinuation**							
Bios vs. Ref	23	10069	0.889	**0.798, 0.990**	**0.033**	22.5	0.16
ADA	10	4587	0.966	0.790, 1.180	0.73	30.8	0.16
ETN	7	2962	0.848	0.683, 1.053	0.13	48.3	0.07
IFX	6	2520	0.861	0.734, 1.009	0.06	0.0	0.71
**IMMUNOGENICITY OUTCOMES**							
**ADAs**							
Bios vs. Ref	24	10151	0.915	**0.869, 0.963**	**0.001**	77.3	<0.001
ADA	10	4532	0.964	0.903, 1.028	0.27	35.2	0.13
ETN	7	2955	0.176	**0.117, 0.265**	**0.001**	74.5	<0.001
IFX	7	2633	1.028	0.945, 1.118	0.52	30.4	0.20
**NAbs**							
Bios vs. Ref	18	NA*	1.014	0.959, 1.071	0.64	0.0	0.99
ADA	8	NA*	1.005	0.919, 1.099	0.90	0.0	0.99
ETN	8	NA*	1.643	0.484, 5.574	0.43	0.0	0.91
IFX	5	NA*	1.028	0.942, 1.122	0.54	0.0	0.48

* The denominators of RR were based on the number of patients with ADAs.

RR comparing Bios and Ref on safety and immunogenicity outcomes by type of reference molecule.

Abbreviations: TEAE, treatment-emergent adverse events; IRRs, infusion-related reactions; ISRs, injection site reactions; ADAs, positive anti-drug antibodies; NAbs, positive neutralizing antibodies; RR, relative risk; Bios, biosimilars; Ref, reference biologics drugs; ADA, adalimumab; ETN, etanercept; IFX, infliximab; M, months; CI, confidence interval; I^2 metric estimates within-study variance; NA, not applicable.

© 2023 de Oliveira Ascef B et al. *JAMA Network Open.*

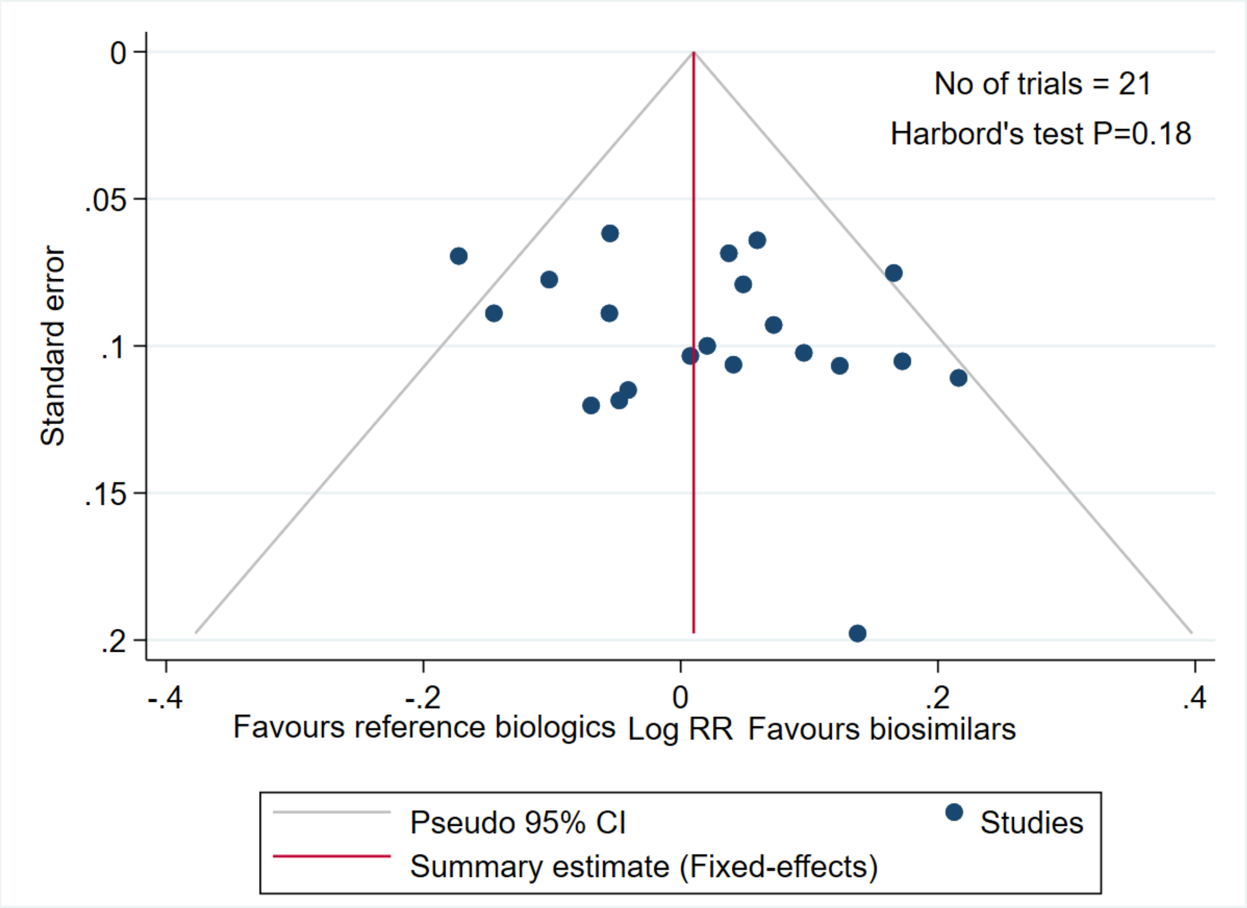

eFigure 8. Funnel plot for the effects of biosimilars group vs. references group on ACR50 at 6 months of follow-up.

Funnel plot of 21 studies. The vertical and diagonal dashed lines represent the overall estimated effect size and its 95% confidence limits, respectively, based on the fixed-effect model. The approximately symmetric shape of funnel plots does not reveal any evidence of publication bias. Harbord's test (*P* = 0.18).
Abbreviations: RR, risk ratio; ACR50, the American College of Rheumatology 50 criteria.

© 2023 de Oliveira Ascef B et al. *JAMA Network Open.*

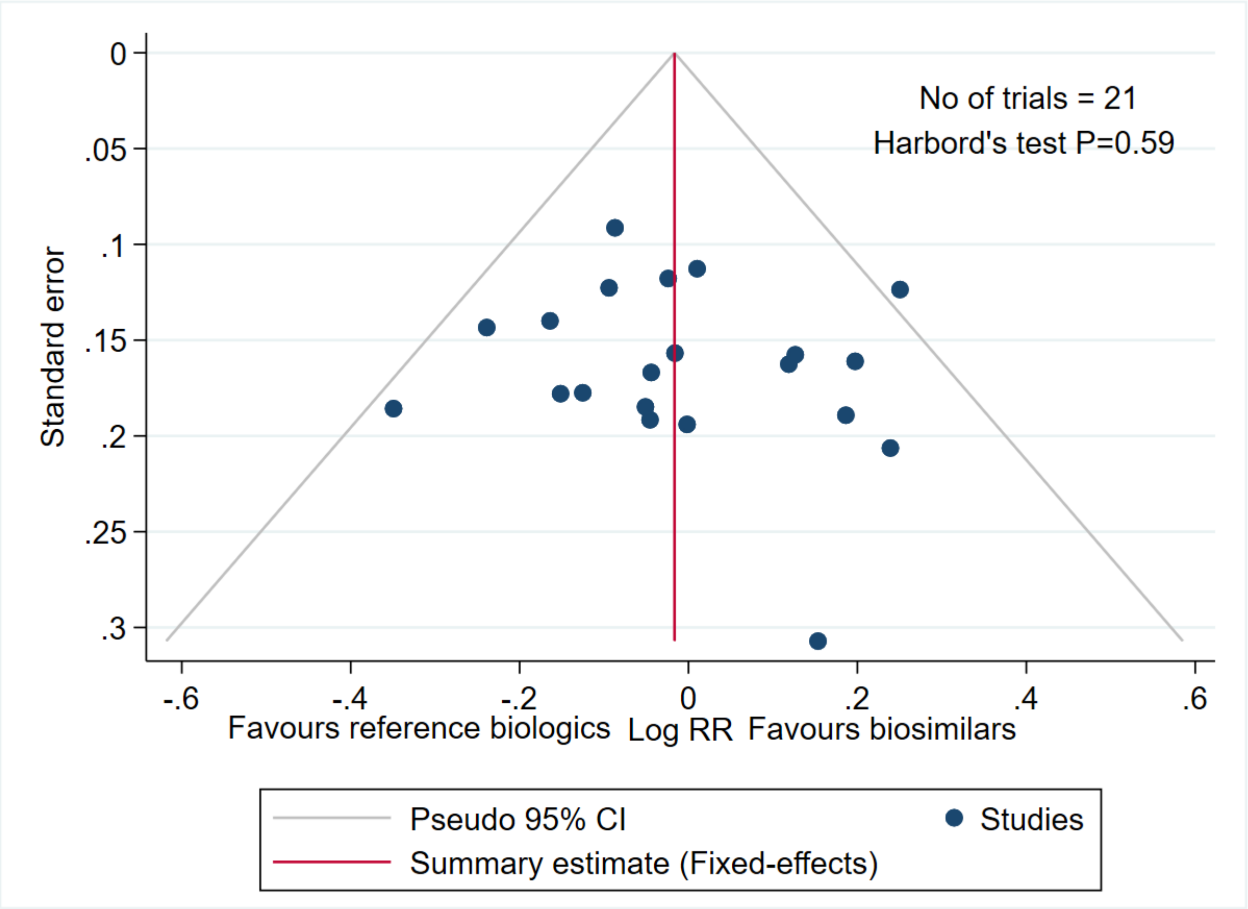

eFigure 9. Funnel plot for the effects of biosimilars group vs. references group on ACR70 at 6 months of follow-up.

Funnel plot of 21 studies. The vertical and diagonal dashed lines represent the overall estimated effect size and its 95% confidence limits, respectively, based on the fixed-effect model. The approximately symmetric shape of funnel plots does not reveal any evidence of publication bias. Harbord's test (*P* = 0.59).
Abbreviations: RR, risk ratio; ACR70, the American College of Rheumatology 70 criteria.

© 2023 de Oliveira Ascef B et al. *JAMA Network Open.*

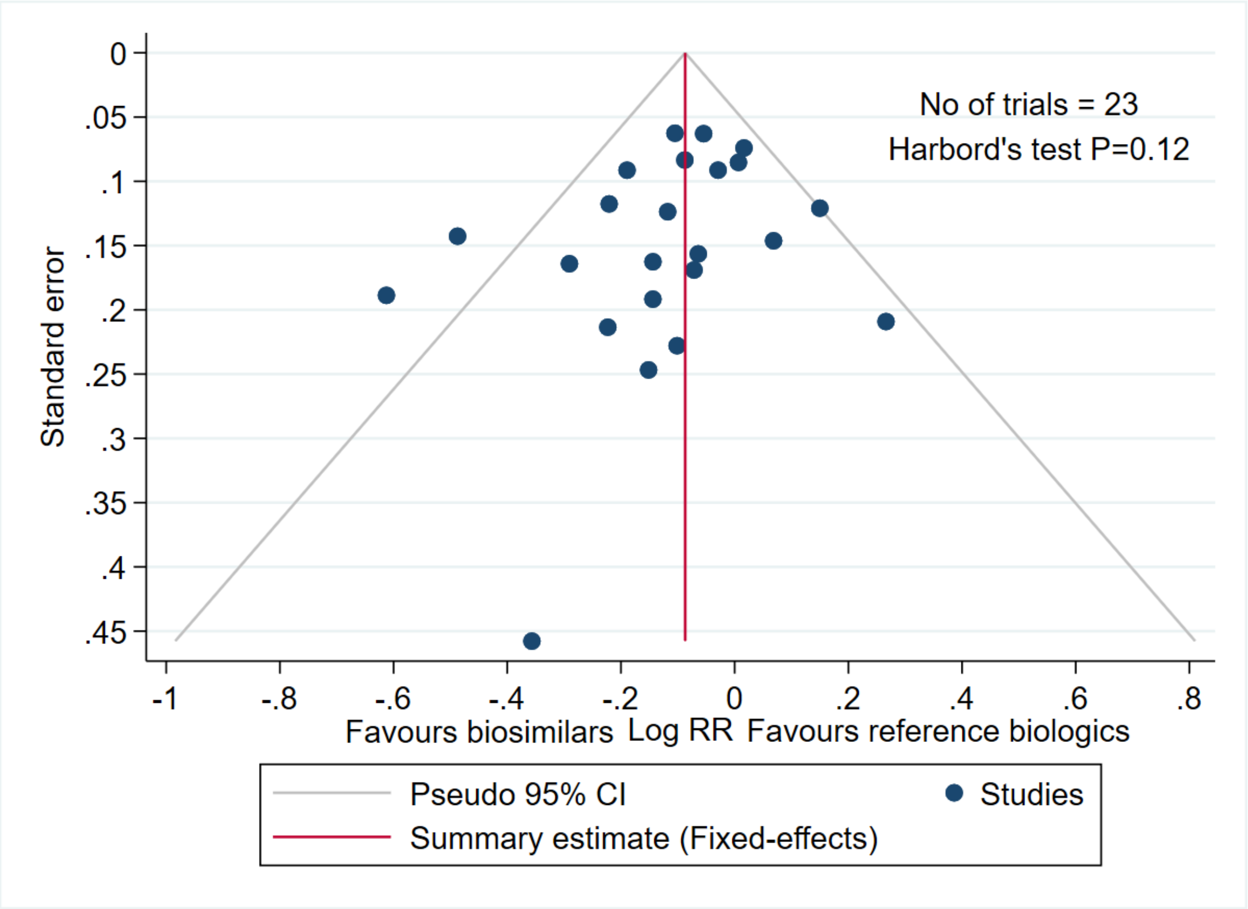

eFigure 10. Funnel plot for the effects of biosimilars group vs. references group on the risk of overall treatment-emergent adverse effects.

Funnel plot of 23 studies. The vertical and diagonal dashed lines represent the overall estimated effect size and its 95% confidence limits, respectively, based on the fixed-effect model. The approximately symmetric shape of funnel plots does not reveal any evidence of publication bias. Harbord's test (P = 0.12).
Abbreviations: RR, risk ratio.

© 2023 de Oliveira Ascef B et al. *JAMA Network Open.*

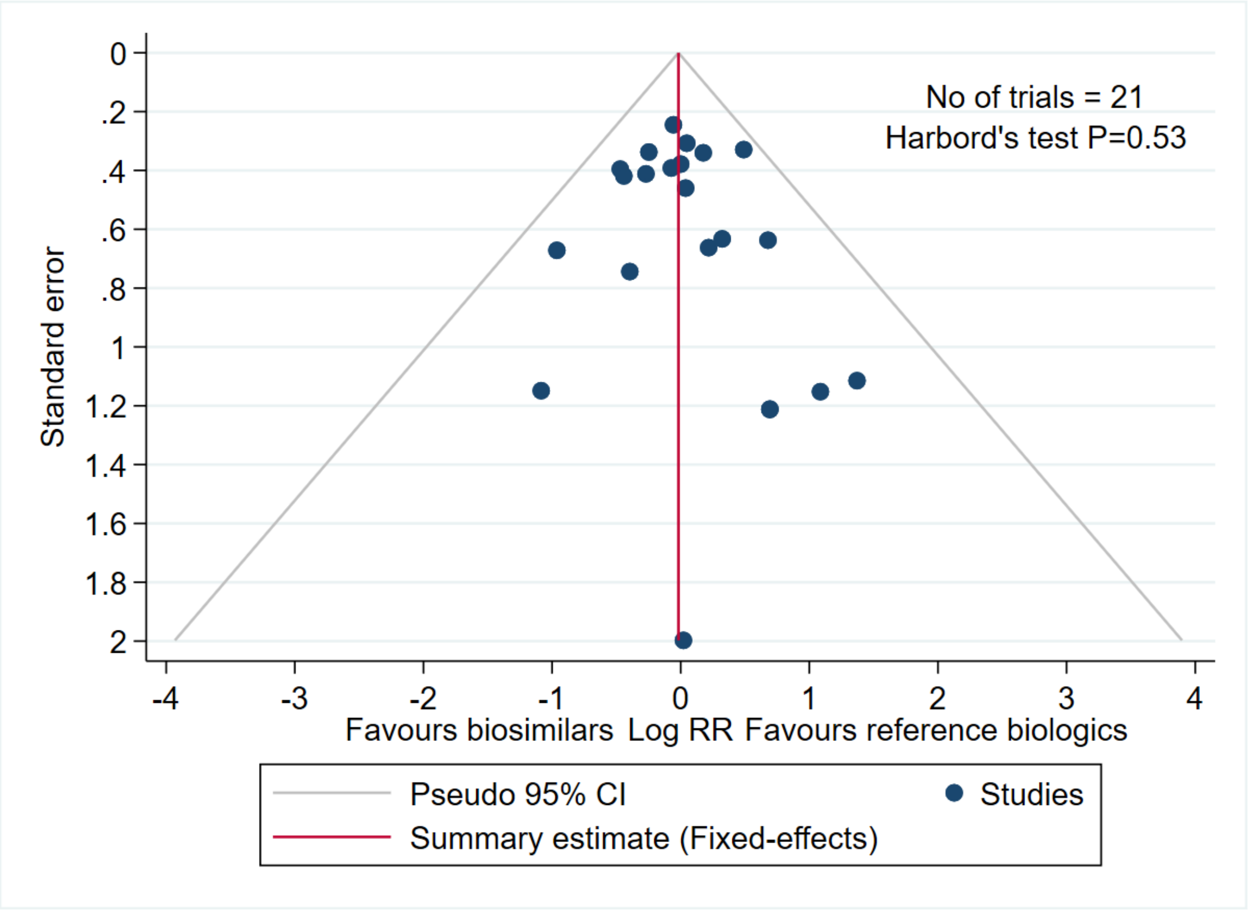

eFigure 11. Funnel plot for the effects of biosimilars group vs. references group on the risk of serious treatment-emergent adverse effects.

Funnel plot of 21 studies. The vertical and diagonal dashed lines represent the overall estimated effect size and its 95% confidence limits, respectively, based on the fixed-effect model. The approximately symmetric shape of funnel plots does not reveal any evidence of publication bias. Harbord's test (*P* = 0.53).
Abbreviations: RR, risk ratio.

© 2023 de Oliveira Ascef B et al. *JAMA Network Open.*

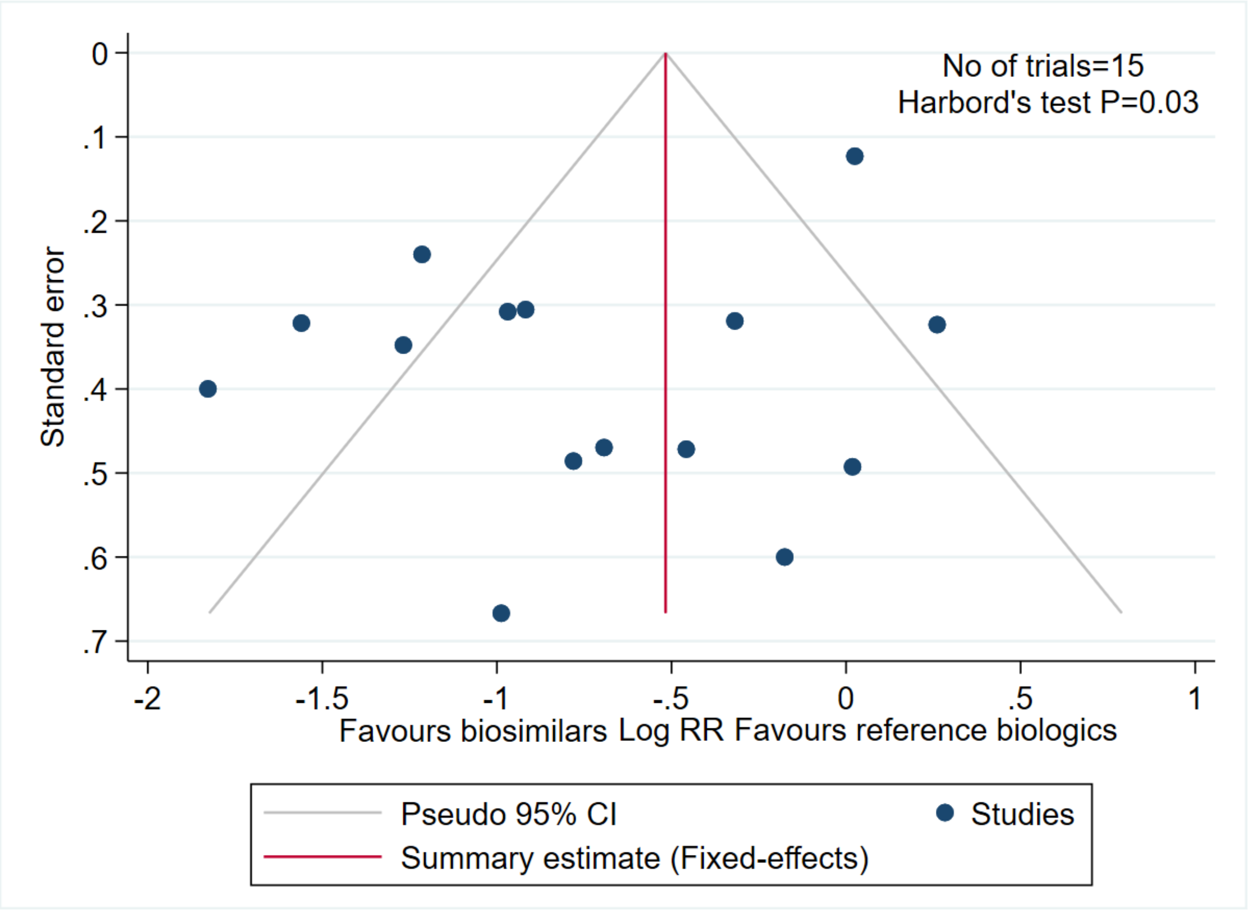

eFigure 12. Funnel plot for the effects of biosimilars group vs. references group on the risk of injection site reactions.

Funnel plot of 15 studies. The vertical and diagonal dashed lines represent the overall estimated effect size and its 95% confidence limits, respectively, based on the fixed-effect model. The approximately symmetric shape of funnel plots revealed evidence of publication bias. Harbord's test (*P* = 0.03).
Abbreviations: RR, risk ratio.

© 2023 de Oliveira Ascef B et al. *JAMA Network Open.*

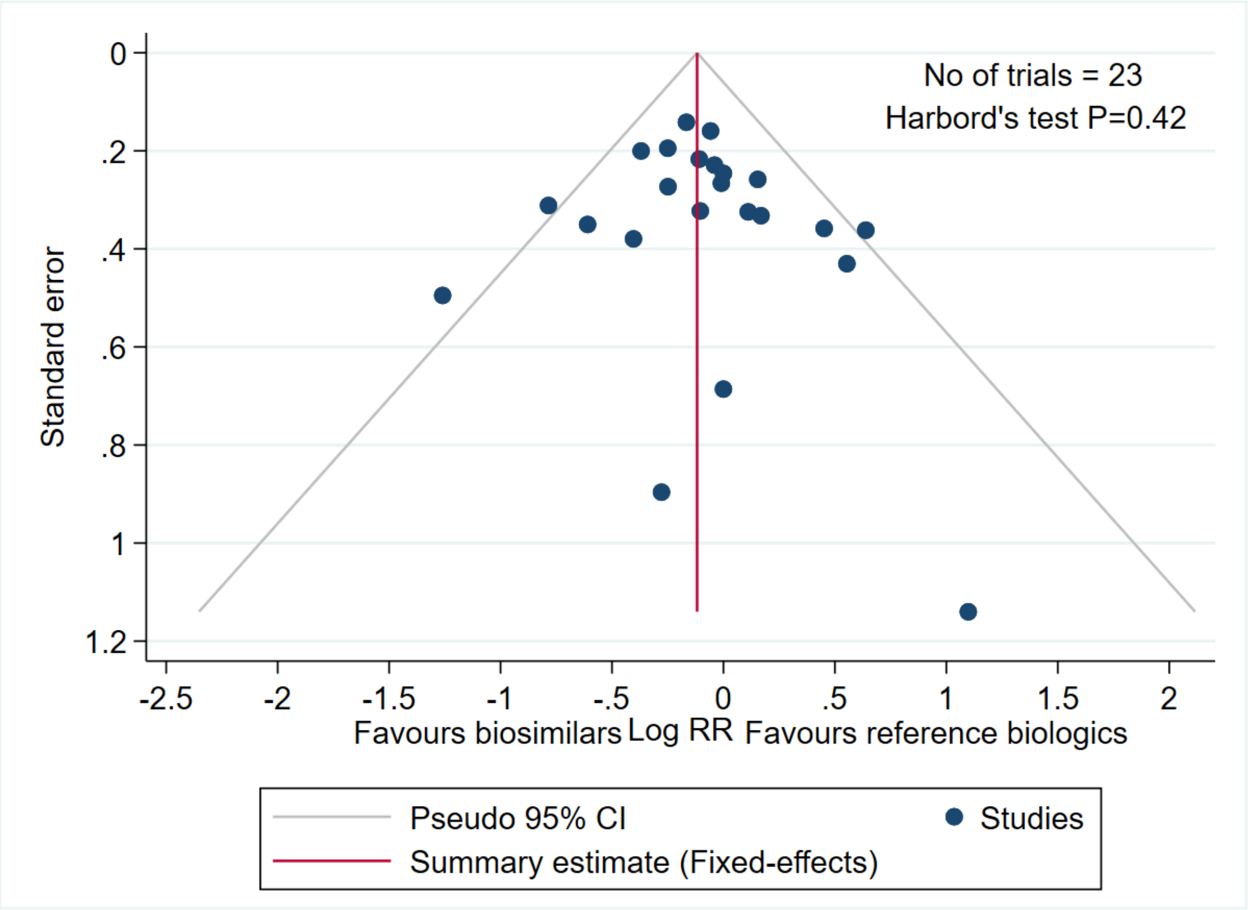

eFigure 13. Funnel plot for the effects of biosimilars group vs. references group on the risk of overall discontinuation rates.

Funnel plot of 23 studies. The vertical and diagonal dashed lines represent the overall estimated effect size and its 95% confidence limits, respectively, based on the fixed-effect model. The approximately symmetric shape of funnel plots does not reveal any evidence of publication bias. Harbord's test (*P* = 0.42).
Abbreviations: RR, risk ratio.

© 2023 de Oliveira Ascef B et al. *JAMA Network Open.*

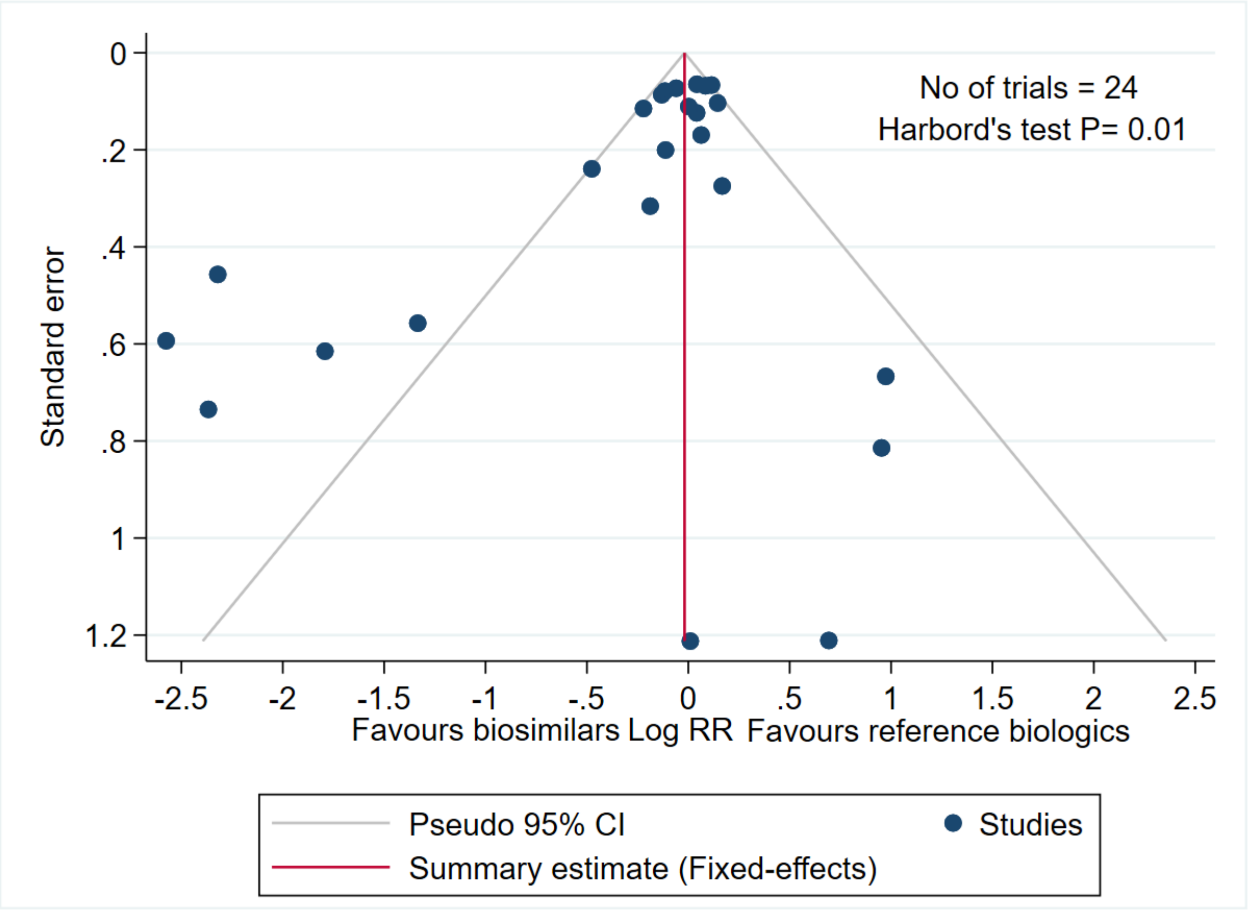

eFigure 14. Funnel plot for the effects of biosimilars group vs. references group on the risk of positive anti-drug antibodies.

Funnel plot of 24 studies. RR denotes risk-ratio. The vertical and diagonal dashed lines represent the overall estimated effect size and its 95% confidence limits, respectively, based on the fixed-effect model. The approximately symmetric shape of funnel plots revealed evidence of publication bias. Harbord's test (*P* =0.01).
Abbreviations: RR, risk ratio.

© 2023 de Oliveira Ascef B et al. *JAMA Network Open.*

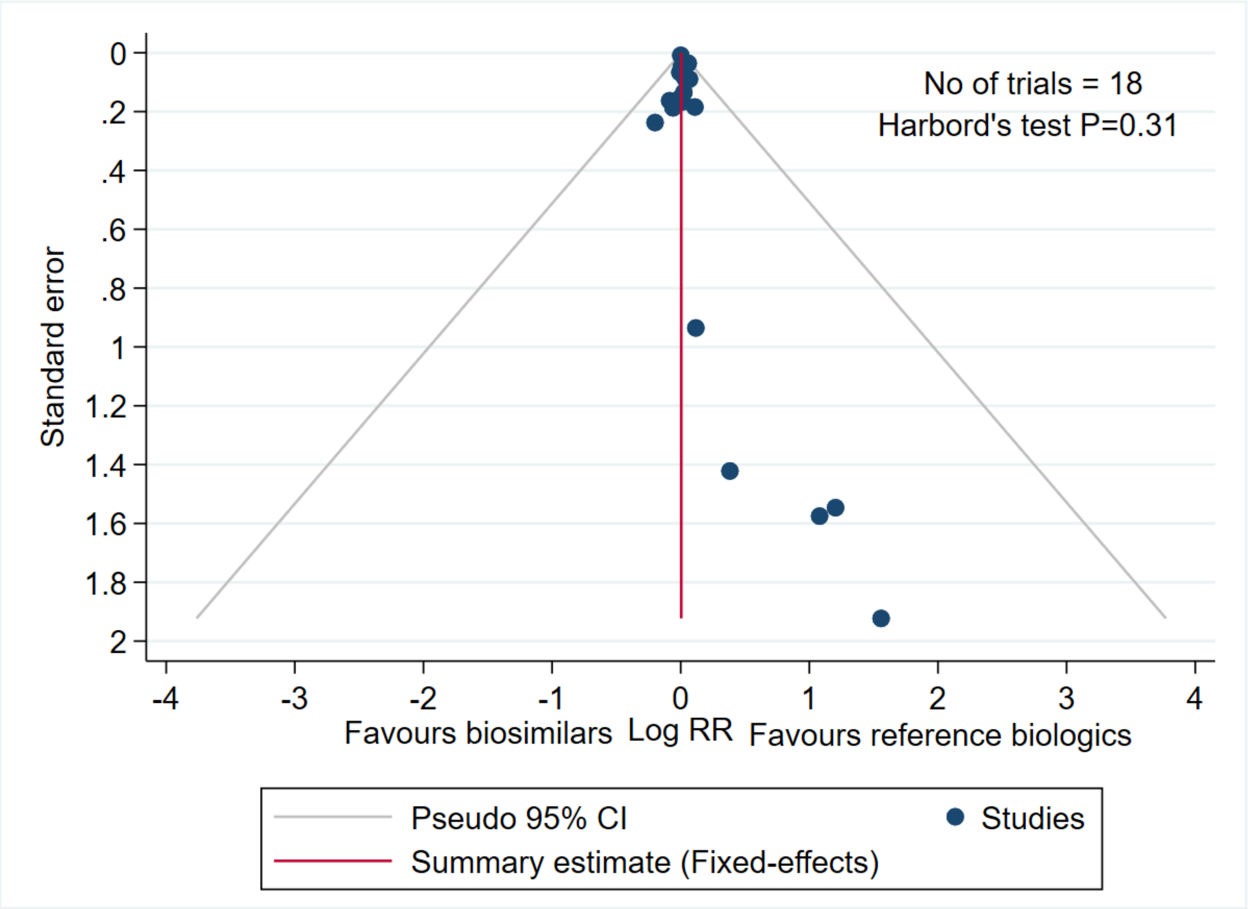

eFigure 15. Funnel plot for the effects of biosimilars group vs. references group on the risk of positive neutralizing antibodies.

Funnel plot of 18 studies. The vertical and diagonal dashed lines represent the overall estimated effect size and its 95% confidence limits, respectively, based on the fixed-effect model. The approximately symmetric shape of funnel plots does not reveal any evidence of publication bias. Harbord's test (*P* =0.31).
Abbreviations: RR, risk ratio.

© 2023 de Oliveira Ascef B et al. *JAMA Network Open.*

eTable 18. Certainty of evidence assessment of trials comparing biosimilars versus their reference biologic drugs in patients with arthritis rheumatoid (GRADE evidence profile).

Outcome	N of studies (n of patients)	Study design	Risk of bias	Inconsistency	Indirect evidence	Imprecision	Publication bias	Relative Effects (95% CrI)	Certainty of Evidence	Importance of outcome
**Primary and coprimary outcomes - Efficacy**										
**ACR 20**	24 studies (9225 patients)	RCT	Serious [a]	No serious	No serious	No serious	No serious [e]	RR: 1.006 (0.985 to 1.030)	⊕⊕⊕◯ Moderate	Critical
**HAQ-DI**	14 studies (5042 patients)	RCT	Serious [a]	No serious	No serious	No serious	No serious	RR: -0.041 (-0.109 to 0.024)	⊕⊕⊕◯ Moderate	Critical
**Secondary outcomes - Efficacy**										
**ACR50**	21 studies (8872 patients)	RCT	Serious [a]	No serious	No serious	No serious	No serious	RR: 1.011 (0.964 to 1.065)	⊕⊕⊕◯ Moderate	Critical
**ACR70**	21 studies (8856 patients)	RCT	Serious [a]	No serious	No serious	No serious	No serious	RR: 0.975 (0.906 to 1.048)	⊕⊕⊕◯ Moderate	Important
**Secondary outcomes - Safety outcomes**										
**Overall TEAE**	23 studies (9586 patients)	RCT	Serious [a]	No serious	Serious [c]	No serious	No serious	RR: 0.900 (0.836 to 0.958)	⊕⊕◯◯ Low	Critical
**Serious TEAE**	21 studies (9021 patients)	RCT	Serious [a]	No serious	No serious	No serious	No serious	RR: 0.949 (0.767 to 1.171)	⊕⊕⊕◯ Moderate	Critical
**Hypersensitivity**	4 studies (2058 patients)	RCT	Serious [a]	No serious	No serious	Serious [d]	NA	RR: 0.726 (0.388 to 1.317)	⊕⊕⊕◯ Moderate	Critical
**IRRs**	6 studies (2505 patients)	RCT	Serious [a]	No serious	No serious	No serious	NA	RR: 0.806 (0.559 to 1.192)	⊕⊕⊕◯ Moderate	Critical
**ISRs**	15 studies (6772 patients)	RCT	Serious [a]	Serious [b]	Serious [c]	Serious [d]	No serious [e]	RR: 0.460 (0.326 to 0.650)	⊕⊕◯◯ Low	Critical
**Overall discontinuation**	23 studies (10151 patients)	RCT	Serious [a]	No serious	No serious	No serious	No serious	RR: 0.876 (0.765 to 1.005)	⊕⊕⊕◯ Moderate	Critical
**Secondary outcomes - Immunogenicity outcomes**										

© 2023 de Oliveira Ascef B et al. *JAMA Network Open.*

Outcome	N of studies (n of patients)	Study design	Risk of bias	Inconsistency	Indirect evidence	Imprecision	Publication bias	Relative Effects (95% CrI)	Certainty of Evidence	Importance of outcome
**ADAs**	24 studies (10151 patients)	RCT	Serious [a]	Serious [b]	Serious [c]	Serious [d]	No serious [e]	RR: 0.684 (0.467 to 0.972)	⊕⊕◯◯ Low	Important
**NAbs**	18 studies (2982 events of ADAs)	RCT	Serious [a]	No serious	Serious [c]	No serious	No serious	RR: 1.036 (0.984 to 1.093)	⊕⊕◯◯ Low	Important

[a] We downgraded by one level because of crucial limitations for incomplete outcome data, or some limitations (unclear risk of bias) for multiple criteria in most of the trials, which would be sufficient to lower confidence in the estimate of effect.

[b] There is a variance of points estimates across studies and high heterogeneity within and between-study (I^2 ISRs = 81.5%, *P*<0.001; Tau^2: 0.305) ((I^2 ADAs= 75.2%, *P*<0.001; Tau^2: 0.620).

[c] We observed inconsistencies in how the outcomes were measured between studies, including different definitions, methods of measurement, and follow-up time.

[d] We downgraded it by one level due to a small number of events (<400) or larger magnitude of the effects estimates, and a wider %95 CrI crossing the prespecified margins of equivalence.

[e] Although it was observed the asymmetry of the funnel plot and *P* values of Harbor's test were statistically significant (*P*<0.10) for these outcomes, we did not consider them as serious risk. First, we did not find any trials that were registered in the trial register databases, but the results were not available. Second, currently there are no validated methods to assess publication bias in equivalence or non-inferiority trials. Specifically for the outcomes ACR20 and HAQ-DI, we performed a subgroup and exploratory analyses restricted to large trials (500 or more randomised participants) to mitigate the possibility of small-study bias, observing similar conclusion to the main analyses.

High certainty: We are very confident that the true effect lies close to that of the estimate of the effect. Moderate certainty: We are moderately confident in the effect estimate: the true effect is likely to be close to the estimate of the effect, but there is a possibility that it is substantially different. Low certainty: Our confidence in the effect estimate is limited: the true effect may be substantially different from the estimate of the effect. Very low certainty: We have very little confidence in the effect estimate: the true effect is likely to be substantially different from the estimate of effect.

Abbreviations: NA, not applicable; RCT, Randomized Clinical Trials; RR, relative risk based on Bayesian random-effects model; SMD, standardized mean differences based on Bayesian random-effects model; ACR0, the American College of Rheumatology 20 criteria; HAQ-DI, Health Assessment Questionnaire-Disability Index; ACR50, the American College of Rheumatology 50 criteria; ACR70, the American College of Rheumatology 70 criteria; TEAE, Overall Treatment-Emergent Adverse Event; IRRs, infusion-related reactions; ISRs, injection site reactions; ADAs, positive anti-drug antibodies; NAbs, positive neutralizing antibodies.

© 2023 de Oliveira Ascef B et al. *JAMA Network Open.*

Supplementary online References.

1. Sutton AJ, Higgins JP. Recent developments in meta-analysis. Stat Med. 2008;27(5):625-50, https://doi.org/10.1002/sim.2934.
2. Wetterslev J, Thorlund K, Brok J, Gluud C. Trial sequential analysis may establish when firm evidence is reached in cumulative meta-analysis. J Clin Epidemiol. 2008;61(1):64-75, https://doi.org/10.1016/j.jclinepi.2007.03.013.
3. Jones CW, Handler L, Crowell KE, Keil LG, Weaver MA, Platts-Mills TF. Non-publication of large randomized clinical trials: cross sectional analysis. BMJ. 2013;347:f6104, https://doi.org/10.1136/bmj.f6104.
4. Palmer TM, Sutton AJ, Peters JL, Moreno SG. Contour-Enhanced Funnel Plots for Meta-Analysis. The Stata Journal: Promoting communications on statistics and Stata. 2018;8(2):242-54, https://doi.org/10.1177/1536867x0800800206.
5. Page MJ, McKenzie JE, Bossuyt PM, Boutron I, Hoffmann TC, Mulrow CD, et al. The PRISMA 2020 statement: an updated guideline for reporting systematic reviews. BMJ. 2021;372(n160):n71, https://doi.org/10.1136/bmj.n71.
6. Smolen JS, Landewe RBM, Bijlsma JWJ, Burmester GR, Dougados M, Kerschbaumer A, et al. EULAR recommendations for the management of rheumatoid arthritis with synthetic and biological disease-modifying antirheumatic drugs: 2019 update. Ann Rheum Dis. 2020;79(6):685-99, https://doi.org/10.1136/annrheumdis-2019-216655.
7. Kay J, Schoels MM, Dorner T, Emery P, Kvien TK, Smolen JS, et al. Consensus-based recommendations for the use of biosimilars to treat rheumatological diseases. Ann Rheum Dis. 2018;77(2):165-74, https://doi.org/10.1136/annrheumdis-2017-211937.
8. Allocati E, Bertele V, Gerardi C, Garattini S, Banzi R. Clinical evidence supporting the marketing authorization of biosimilars in Europe. Eur J Clin Pharmacol. 2020;76(4):557-66, https://doi.org/10.1007/s00228-019-02805-y.
9. Ascef BO, Almeida MO, de Medeiros Ribeiro AC, Andrade DCO, de Oliveira Junior HA, Pereira TV, et al. Equivalence and switching between biosimilars and reference molecules in rheumatoid arthritis: protocol for a systematic review and meta-analysis. Syst Rev. 2021;10(1):205, https://doi.org/10.1186/s13643-021-01754-x.
10. US Food and Drug Administration. Considerations in demonstrating interchangeability with a reference product: guidance for industry. In: U.S. Department of Health and Human Services, editor. Silver Spring: US Food and Drug Administration; 2019.
11. Treadwell JR, Uhl S, Tipton K, Shamliyan T, Viswanathan M, Berkman ND, et al. Assessing equivalence and noninferiority. In: Methods Research Report, editor. (Prepared by the EPC Workgroup under Contract No 290-2007-10063). Agency for Healthcare Research and Quality: Agency for Healthcare Research and Quality; 2012. p. 1144-9.
12. World Health Organization. Guidelines on evaluation of similar biotherapeutic products (SBPs). Geneva, Switzerland: World Health Organization; 2009.
13. Felson DT, Anderson JJ, Boers M, Bombardier C, Furst D, Goldsmith C, et al. American College of Rheumatology. Preliminary definition of improvement in rheumatoid arthritis. Arthritis Rheum. 1995;38(6):727-35, https://doi.org/10.1002/art.1780380602.
14. Bruce B, Fries JF. The Stanford Health Assessment Questionnaire: a review of its history, issues, progress, and documentation. J Rheumatol. 2003;30(1):167-78.
15. Horta-Baas G. Patient-Reported Outcomes in Rheumatoid Arthritis: A Key Consideration for Evaluating Biosimilar Uptake? Patient Relat Outcome Meas. 2022;13:79-95, https://doi.org/10.2147/PROM.S256715.

© 2023 de Oliveira Ascef B et al. *JAMA Network Open.*

16. Gossec L, Dougados M, Dixon W. Patient-reported outcomes as end points in clinical trials in rheumatoid arthritis. RMD Open. 2015;1(1):e000019, https://doi.org/10.1136/rmdopen-2014-000019.
17. Kluzek S, Dean B, Wartolowska KA. Patient-reported outcome measures (PROMs) as proof of treatment efficacy. BMJ Evid Based Med. 2022;27(3):153-5, https://doi.org/10.1136/bmjebm-2020-111573.
18. Higgins JPT GSe. Cochrane Handbook for Systematic Reviews of Interventions Version 5.1.0: The Cochrane Collaboration; 2011 [Available from: https://training.cochrane.org/handbook/archive/v5.1/.
19. McGuinness LA, Higgins JPT. Risk-of-bias VISualization (robvis): An R package and Shiny web app for visualizing risk-of-bias assessments. Res Synth Methods. 2021;12(1):55-61, https://doi.org/10.1002/jrsm.1411.
20. Kwon D, Reis IM. Simulation-based estimation of mean and standard deviation for meta-analysis via Approximate Bayesian Computation (ABC). BMC Med Res Methodol. 2015;15:61, https://doi.org/10.1186/s12874-015-0055-5.
21. Walker E, Nowacki AS. Understanding equivalence and noninferiority testing. J Gen Intern Med. 2011;26(2):192-6, https://doi.org/10.1007/s11606-010-1513-8.
22. Jones B, Jarvis P, Lewis JA, Ebbutt AF. Trials to assess equivalence: the importance of rigorous methods. BMJ. 1996;313(7048):36-9, https://doi.org/10.1136/bmj.313.7048.36.
23. Peters JL, Sutton AJ, Jones DR, Abrams KR, Rushton L. Contour-enhanced meta-analysis funnel plots help distinguish publication bias from other causes of asymmetry. J Clin Epidemiol. 2008;61(10):991-6, https://doi.org/10.1016/j.jclinepi.2007.11.010.
24. Wetterslev J, Jakobsen JC, Gluud C. Trial Sequential Analysis in systematic reviews with meta-analysis. BMC Med Res Methodol. 2017;17(1):39, https://doi.org/10.1186/s12874-017-0315-7.
25. O'Brien PC, Fleming TR. A Multiple Testing Procedure for Clinical Trials. Biometrics. 1979;35(3), https://doi.org/10.2307/2530245.
26. Thorlund K EJ, Wetterslev J, Brok J, Imberger G, Gluud C,. Trial Sequential Analysis (TSA) Copenhagen Copenhagen Trials Unit; 2011.
27. Schünemann H, Brożek J, Guyatt G, Oxman A, editors. GRADE handbook for grading quality of evidence and strength of recommendations: The GRADE Working Group 2013 [updated October 2013. Available from: https://gdt.gradepro.org/app/handbook/handbook.html#h.1i2bwkm8zpjo.
28. Acuna SA, Dossa F, Baxter N. Meta-analysis of noninferiority and equivalence trials: ignoring trial design leads to differing and possibly misleading conclusions. J Clin Epidemiol. 2020;127:134-41, https://doi.org/10.1016/j.jclinepi.2020.05.034.
29. Jani RH, Gupta R, Bhatia G, Rathi G, Ashok Kumar P, Sharma R, et al. A prospective, randomized, double-blind, multicentre, parallel-group, active controlled study to compare efficacy and safety of biosimilar adalimumab (Exemptia; ZRC-3197) and adalimumab (Humira) in patients with rheumatoid arthritis. Int J Rheum Dis. 2015;19(11):1157-68, https://doi.org/10.1111/1756-185X.12711.
30. Alten R, Glover J, Matsunaga N, Chisholm D, Genovese M. OP0021 Efficacy and safety results of a phase iii study comparing fkb327, an adalimumab biosimilar, with the adalimumab reference product in patients with active rheumatoid arthritis. Oral Presentations: Ann Rheum Dis; 2017. p. 59-, https://doi.org/10.1136/annrheumdis-2017-eular.2220

© 2023 de Oliveira Ascef B et al. *JAMA Network Open.*

31. Genovese MC, Glover J, Matsunaga N, Chisholm D, Alten R. 2799 Efficacy, Safety and Immunogenicity in Randomized, Double-Blind (DB) and OpenLabel Extension (OLE) Studies Comparing FKB327, an Adalimumab Biosimilar, with the Adalimumab Reference Product (Humira (R); RP) in Patients (pts) with Active Rheumatoid Arthritis (RA). In: Wiley, editor. 2017 ACR ARHP Annual Meeting; NJ USA: Arthritis Reumatol; 2017,
32. Alten R, Genovese MC, Muniz R, Kellner H. Sat0132 Long-Term Safety, Immunogenicity and Efficacy in Randomized, Double-Blind, and Open-Label Extension Studies Comparing Fkb327, an Adalimumab Biosimilar, with the Adalimumab Reference Product in Patients with Active Rheumatoid Arthritis. Ann Rheum Dis; 2019. p. 1135.1-, https://doi.org/10.1136/annrheumdis-2019-eular.972
33. Alten R, Markland C, Kawakami K, Boyce M CF, Muniz R, Genovese MC. P422 Immunogenicity of a proposed adalimumab biosimilar, FKB327, and the reference product in patients with rheumatoid arthritis. Journal of Crohn's and Colitis; 2019. p. S320-S, https://doi.org/10.1093/ECCO-JCC/JJY222.546
34. Genovese MC, Glover J, Greenwald M, Porawska W, El Khouri EC, Dokoupilova E, et al. FKB327, an adalimumab biosimilar, versus the reference product: results of a randomized, Phase III, double-blind study, and its open-label extension. Arthritis Res Ther. 2019;21(1):281, https://doi.org/10.1186/s13075-019-2046-0.
35. Alten R, Markland C, Boyce M, Kawakami K, Muniz R, Genovese MC. Immunogenicity of an adalimumab biosimilar, FKB327, and its reference product in patients with rheumatoid arthritis. Int J Rheum Dis. 2020;23(11):1514-25, https://doi.org/10.1111/1756-185X.13951.
36. Genovese MC, Kellner H, Arai Y, Muniz R, Alten R. Long-term safety, immunogenicity and efficacy comparing FKB327 with the adalimumab reference product in patients with active rheumatoid arthritis: data from randomised double-blind and open-label extension studies. RMD Open. 2020;6(1), https://doi.org/10.1136/rmdopen-2019-000987.
37. Cohen S, Genovese MC, Choy E, Perez-Ruiz F, Matsumoto A, Pavelka K, et al. Efficacy and safety of the biosimilar ABP 501 compared with adalimumab in patients with moderate to severe rheumatoid arthritis: a randomised, double-blind, phase III equivalence study. Ann Rheum Dis. 2017;76(10):1679-87, https://doi.org/10.1136/annrheumdis-2016-210459.
38. Cohen S, Pablos JL, Pavelka K, Muller GA, Matsumoto A, Kivitz A, et al. An open-label extension study to demonstrate long-term safety and efficacy of ABP 501 in patients with rheumatoid arthritis. Arthritis Res Ther. 2019;21(1):84, https://doi.org/10.1186/s13075-019-1857-3.
39. Jamshidi A, Gharibdoost F, Vojdanian M, Soroosh SG, Soroush M, Ahmadzadeh A, et al. A phase III, randomized, two-armed, double-blind, parallel, active controlled, and non-inferiority clinical trial to compare efficacy and safety of biosimilar adalimumab (CinnoRA(R)) to the reference product (Humira(R)) in patients with active rheumatoid arthritis. Arthritis Res Ther. 2017;19(1):168, https://doi.org/10.1186/s13075-017-1371-4.
40. Fleischmann RM, Alten R, Pileckyte M, Lobello K, Hua SY, Cronenberger C, et al. A comparative clinical study of PF-06410293, a candidate adalimumab biosimilar, and adalimumab reference product (Humira(R)) in the treatment of active rheumatoid arthritis. Arthritis Res Ther. 2018;20(1):178, https://doi.org/10.1186/s13075-018-1676-y.
41. Fleischmann RM, Alvarez DF, Bock AE, Cronenberger C, Vranic I, Zhang W, et al. Randomised study of PF-06410293, an adalimumab (ADL) biosimilar, compared with reference ADL for the treatment of active rheumatoid arthritis: results from weeks 26-52, including a

© 2023 de Oliveira Ascef B et al. *JAMA Network Open.*

treatment switch from reference ADL to PF-06410293. RMD Open. 2021;7(2), https://doi.org/10.1136/rmdopen-2021-001578.
42. Fleischmann R, Alvarez D, Bock A, Cronenberger C, Vranic I, Zhang W, et al. A Randomized, Double-blind Phase 3 Study Comparing the Efficacy, Safety and Immunogenicity of PF-06410293 (Abrilada™), an Adalimumab (ADL) Biosimilar, and Reference ADL (Humira®) in Patients with Moderate to Severe Active RA: Results from Weeks 52-92. ACR Convergence 2020; Berlin, Germany: Arthritis Rheumatol; 2020,
43. Cohen SB, Alonso-Ruiz A, Klimiuk PA, Lee EC, Peter N, Sonderegger I, et al. Similar efficacy, safety and immunogenicity of adalimumab biosimilar BI 695501 and Humira reference product in patients with moderately to severely active rheumatoid arthritis: results from the phase III randomised VOLTAIRE-RA equivalence study. Ann Rheum Dis. 2018;77(6):914-21, https://doi.org/10.1136/annrheumdis-2017-212245.
44. Cohen SB, Czeloth N, Lee E, Klimiuk PA, Peter N, Jayadeva G. Long-term safety, efficacy, and immunogenicity of adalimumab biosimilar BI 695501 and adalimumab reference product in patients with moderately-to-severely active rheumatoid arthritis: results from a phase 3b extension study (VOLTAIRE-RAext). Expert Opin Biol Ther. 2019;19(10):1097-105, https://doi.org/10.1080/14712598.2019.1645114.
45. Weinblatt ME, Baranauskaite A, Niebrzydowski J, Dokoupilova E, Zielinska A, Jaworski J, et al. Phase III Randomized Study of SB5, an Adalimumab Biosimilar, Versus Reference Adalimumab in Patients With Moderate-to-Severe Rheumatoid Arthritis. Arthritis Rheumatol. 2018;70(1):40-8, https://doi.org/10.1002/art.40336.
46. Weinblatt ME, Baranauskaite A, Dokoupilova E, Zielinska A, Jaworski J, Racewicz A, et al. Switching From Reference Adalimumab to SB5 (Adalimumab Biosimilar) in Patients With Rheumatoid Arthritis: Fifty-Two-Week Phase III Randomized Study Results. Arthritis Rheumatol. 2018;70(6):832-40, https://doi.org/10.1002/art.40444.
47. Edwards CJ, Monnet J, Ullmann M, Vlachos P, Chyrok V, Ghori V. Safety of adalimumab biosimilar MSB11022 (acetate-buffered formulation) in patients with moderately-to-severely active rheumatoid arthritis. Clin Rheumatol. 2019;38(12):3381-90, https://doi.org/10.1007/s10067-019-04679-y.
48. Wiland P, Jeka S, Dokoupilova E, Miranda Limon JM, Jauch-Lembach J, Thakur A, et al. Fri0087 Efficacy, Safety, and Immunogenicity Results of the Switch from Reference Adalimumab (Refadl) to Sandoz Biosimilar Adalimumab (Gp2017, Sdz-Adl) from Admyra Phase 3 Study in Patients with Moderate-to-Severe Rheumatoid Arthritis (Ra). Poster Presentations: Arthritis Rheumatol 2019. p. 706.2-7, https://doi.org/10.1136/annrheumdis-2019-eular.960
49. Wiland P, Jeka S, Dokoupilova E, Brandt-Jurgens J, Miranda Limon JM, Cantalejo Moreira M, et al. Switching to Biosimilar SDZ-ADL in Patients with Moderate-to-Severe Active Rheumatoid Arthritis: 48-Week Efficacy, Safety and Immunogenicity Results From the Phase III, Randomized, Double-Blind ADMYRA Study. BioDrugs. 2020;34(6):809-23, https://doi.org/10.1007/s40259-020-00447-6.
50. Matsuno H, Kang YM, Okada M, Lee SI, Park SH, Sheen DH, et al. Comparison of the efficacy and safety of LBAL, a candidate adalimumab biosimilar, and adalimumab reference product in patients with active rheumatoid arthritis inadequately responding to methotrexate: a 52-week phase III randomised study. Clinical and Experimental Rheumatology; 2021, https://doi.org/10.55563/clinexprheumatol/cyudn8
51. Kay J, Jaworski J, Wojciechowski R, Wiland P, Dudek A, Krogulec M, et al. Efficacy and safety of biosimilar CT-P17 versus reference adalimumab in subjects with rheumatoid arthritis:

© 2023 de Oliveira Ascef B et al. *JAMA Network Open.*

24-week results from a randomized study. Arthritis Res Ther. 2021;23(1):51, https://doi.org/10.1186/s13075-020-02394-7.
52. Furst DE, Jaworski J, Wojciechowski R, Wiland P, Dudek A, Krogulec M, et al. Efficacy and safety of switching from reference adalimumab to CT-P17 (100 mg/ml): 52-week randomized, double-blind study in rheumatoid arthritis. Rheumatology. 2021;0:1–11, https://doi.org/10.1093/rheumatology/keab46017.
53. Emery P, Vencovsky J, Sylwestrzak A, Leszczynski P, Porawska W, Baranauskaite A, et al. A phase III randomised, double-blind, parallel-group study comparing SB4 with etanercept reference product in patients with active rheumatoid arthritis despite methotrexate therapy. Ann Rheum Dis. 2015;76(1):51-7, https://doi.org/10.1136/annrheumdis-2015-207588.
54. Emery P, Vencovsky J, Sylwestrzak A, Leszczynski P, Porawska W, Baranauskaite A, et al. 52-week results of the phase 3 randomized study comparing SB4 with reference etanercept in patients with active rheumatoid arthritis. Rheumatology (Oxford). 2017;56(12):2093-101, https://doi.org/10.1093/rheumatology/kex269.
55. Emery P, Vencovsky J, Sylwestrzak A, Leszczynski P, Porawska W, Stasiuk B, et al. Long-term efficacy and safety in patients with rheumatoid arthritis continuing on SB4 or switching from reference etanercept to SB4. Ann Rheum Dis. 2017, https://doi.org/10.1136/annrheumdis-2017-211591.
56. Bae SC, Kim J, Choe JY, Park W, Lee SH, Park YB, et al. A phase III, multicentre, randomised, double-blind, active-controlled, parallel-group trial comparing safety and efficacy of HD203, with innovator etanercept, in combination with methotrexate, in patients with rheumatoid arthritis: the HERA study. Ann Rheum Dis. 2016;76(1):65-71, https://doi.org/10.1136/annrheumdis-2015-207613.
57. O'Dell J, Takeuchi T, Tanaka Y, Louw I, Tiabut T, Kai M, et al. OP0226 randomized, double-blind study comparing CHS-0214 with etanercept in patients with active rheumatoid arthritis (RA) despite methotrexate (MTX) therapy.: Annals of the Rheumatic Diseases 2016. p. 143-, https://doi.org/10.1136/annrheumdis-2016-eular.1800
58. O'Dell J, Kivitz A, Takeuchi T, Tanaka Y, Louw I, Tiabut T, et al. SAT0162 Switching from etanercept to CHS-0214: a one year, randomized, double-blind study in patients with rheumatoid arthritis. Poster Presentations: Ann Rheum Dis; 2017. p. 831.2-, https://doi.org/10.1136/annrheumdis-2017-eular.2480
59. Matsuno H, Tomomitsu M, Hagino A, Shin S, Lee J, Song YW. Phase III, multicentre, double-blind, randomised, parallel-group study to evaluate the similarities between LBEC0101 and etanercept reference product in terms of efficacy and safety in patients with active rheumatoid arthritis inadequately responding to methotrexate. Ann Rheum Dis. 2017;77(4):488-94, https://doi.org/10.1136/annrheumdis-2017-212172.
60. Park MC, Matsuno H, Kim J, Park SH, Lee SH, Park YB, et al. Long-term efficacy, safety and immunogenicity in patients with rheumatoid arthritis continuing on an etanercept biosimilar (LBEC0101) or switching from reference etanercept to LBEC0101: an open-label extension of a phase III multicentre, randomised, double-blind, parallel-group study. Arthritis Res Ther. 2019;21(1):122, https://doi.org/10.1186/s13075-019-1910-2.
61. Matucci-Cerinic M, Allanore Y, Kavanaugh A, Buch MH, Schulze-Koops H, Kucharz EJ, et al. Efficacy, safety and immunogenicity of GP2015, an etanercept biosimilar, compared with the reference etanercept in patients with moderate-to-severe rheumatoid arthritis: 24-week results from the comparative phase III, randomised, double-blind EQUIRA study. RMD Open. 2018;4(2):e000757, https://doi.org/10.1136/rmdopen-2018-000757.

© 2023 de Oliveira Ascef B et al. *JAMA Network Open.*

62. Jaworski J, Matucci-Cerinic M, Schulze-Koops H, Buch MH, Kucharz EJ, Allanore Y, et al. Switch from reference etanercept to SDZ ETN, an etanercept biosimilar, does not impact efficacy, safety, and immunogenicity of etanercept in patients with moderate-to-severe rheumatoid arthritis: 48-week results from the phase III, randomized, double-blind EQUIRA study. Arthritis Res Ther. 2019;21(1):130, https://doi.org/10.1186/s13075-019-1907-x.
63. Yamanaka H, Kamatani N, Tanaka Y, Hibino T, Drescher E, Sanchez-Burson J, et al. A Comparative Study to Assess the Efficacy, Safety, and Immunogenicity of YLB113 and the Etanercept Reference Product for the Treatment of Patients with Rheumatoid Arthritis. Rheumatol Ther. 2020;7(1):149-63, https://doi.org/10.1007/s40744-019-00186-3.
64. Strusberg I, Mysler E, Citera G, Siri D, de Los Angeles Correa M, Lazaro MA, et al. Efficacy, Safety, and Immunogenicity of Biosimilar Etanercept (Enerceptan) Versus Its Original Form in Combination With Methotrexate in Patients With Rheumatoid Arthritis: A Randomized, Multicenter, Evaluator-Blinded, Noninferiority Study. J Clin Rheumatol. 2021;27(6S):S173-S9, https://doi.org/10.1097/RHU.0000000000001616.
65. Yoo DH, Hrycaj P, Miranda P, Ramiterre E, Piotrowski M, Shevchuk S, et al. A randomised, double-blind, parallel-group study to demonstrate equivalence in efficacy and safety of CT-P13 compared with innovator infliximab when coadministered with methotrexate in patients with active rheumatoid arthritis: the PLANETRA study. Ann Rheum Dis. 2013;72(10):1613-20, https://doi.org/10.1136/annrheumdis-2012-203090.
66. Yoo DH, Racewicz A, Brzezicki J, Yatsyshyn R, Arteaga ET, Baranauskaite A, et al. A phase III randomized study to evaluate the efficacy and safety of CT-P13 compared with reference infliximab in patients with active rheumatoid arthritis: 54-week results from the PLANETRA study. Arthritis Res Ther. 2016;18:82, https://doi.org/10.1186/s13075-016-0981-6.
67. Yoo DH, Prodanovic N, Jaworski J, Miranda P, Ramiterre E, Lanzon A, et al. Efficacy and safety of CT-P13 (biosimilar infliximab) in patients with rheumatoid arthritis: comparison between switching from reference infliximab to CT-P13 and continuing CT-P13 in the PLANETRA extension study. Ann Rheum Dis. 2017;76(2):355-63, https://doi.org/10.1136/annrheumdis-2015-208786.
68. Kay J, Chopra A, Chandrashekara S, Olakkengil DJ, Bhojani KS, Bhatia G, et al. OP0012 A Phase 3, Randomized, Double-Blind, Active Comparator Study of the Efficacy and Safety of Bow015, A Biosimilar Infliximab, in Patients with Active Rheumatoid Arthritis on Stable Methotrexate Doses. Annals of the Rheumatic Diseases2014. p. 64.1-, https://doi.org/10.1136/annrheumdis-2014-eular.1595
69. Taylor P, Wyand M, Knight A, Costantino C, Lassen C. FRI0163 Efficacy of The Biosimilar BOW015, Compared To Originator Infliximab, Initiated at Moderate and Severe Disease Activity Thresholds in Rheumatoid Arthritis. Annals of the Rheumatic Diseases2016. p. 488.2-9, https://doi.org/10.1136/annrheumdis-2016-eular.4143
70. Choe JY, Prodanovic N, Niebrzydowski J, Staykov I, Dokoupilova E, Baranauskaite A, et al. A randomised, double-blind, phase III study comparing SB2, an infliximab biosimilar, to the infliximab reference product Remicade in patients with moderate to severe rheumatoid arthritis despite methotrexate therapy. Ann Rheum Dis. 2017;76(1):58-64, https://doi.org/10.1136/annrheumdis-2015-207764.
71. Smolen JS, Choe JY, Prodanovic N, Niebrzydowski J, Staykov I, Dokoupilova E, et al. Comparing biosimilar SB2 with reference infliximab after 54 weeks of a double-blind trial: clinical, structural and safety results. Rheumatology (Oxford). 2017;56(10):1771-9, https://doi.org/10.1093/rheumatology/kex254.

© 2023 de Oliveira Ascef B et al. *JAMA Network Open.*

72. Smolen JS, Choe JY, Prodanovic N, Niebrzydowski J, Staykov I, Dokoupilova E, et al. Safety, immunogenicity and efficacy after switching from reference infliximab to biosimilar SB2 compared with continuing reference infliximab and SB2 in patients with rheumatoid arthritis: results of a randomised, double-blind, phase III transition study. Ann Rheum Dis. 2018;77(2):234-40, https://doi.org/10.1136/annrheumdis-2017-211741.
73. Takeuchi T, Yamanaka H, Tanaka Y, Sakurai T, Saito K, Ohtsubo H, et al. Evaluation of the pharmacokinetic equivalence and 54-week efficacy and safety of CT-P13 and innovator infliximab in Japanese patients with rheumatoid arthritis. Mod Rheumatol. 2015;25(6):817-24, https://doi.org/10.3109/14397595.2015.1022297.
74. Matsuno H, Matsubara T. A randomized double-blind parallel-group phase III study to compare the efficacy and safety of NI-071 and infliximab reference product in Japanese patients with active rheumatoid arthritis refractory to methotrexate. Mod Rheumatol. 2018;29(6):919-27, https://doi.org/10.1080/14397595.2018.1533063.
75. Lila AM, Mazurov VI, Denisov LN, Nesmeyanova OB, Ilivanova EP, Eremeeva AV, et al. A phase III study of BCD-055 compared with innovator infliximab in patients with active rheumatoid arthritis: 54-week results from the LIRA study. Rheumatol Int. 2019;39(9):1537-46, https://doi.org/10.1007/s00296-019-04359-9.
76. Genovese MC, Sanchez-Burson J, Oh M, Balazs E, Neal J, Everding A, et al. Comparative clinical efficacy and safety of the proposed biosimilar ABP 710 with infliximab reference product in patients with rheumatoid arthritis. Arthritis Res Ther. 2020;22(1):60, https://doi.org/10.1186/s13075-020-2142-1.

© 2023 de Oliveira Ascef B et al. *JAMA Network Open.*
