# Supplementary material for: Therapeutic Equivalence of Biosimilar and Reference Biologic Drugs in Rheumatoid Arthritis: A Systematic Review and Meta-analysis
Source: JAMA Netw Open. 2023 May 26;6(5):e2315872. doi: 10.1001/jamanetworkopen.2023.15872 (PMC10220520; doi:10.1001/jamanetworkopen.2023.15872)
Supplement: Supplement 2. — Data Sharing Statement [file jamanetwopen-e2315872-s002.pdf]

Data Sharing Statement

Ascef. Therapeutic Equivalence of Biosimilar and Reference Biologic Drugs in Rheumatoid Arthritis. *JAMA Netw Open*. Published May 26, 2023.
doi:10.1001/jamanetworkopen.2023.15872

Data
Data available: No

Additional Information
Explanation for why data not available: Data available: No Additional Information The datasets used and/or analysed during the current study are available from the corresponding author upon reasonable request.
